# Carbapenem-Nonsusceptible *Enterobacteriaceae* in Taiwan

**DOI:** 10.1371/journal.pone.0121668

**Published:** 2015-03-20

**Authors:** Jann-Tay Wang, Un-In Wu, Tsai-Ling Yang Lauderdale, Mei-Chen Chen, Shu-Ying Li, Le-Yin Hsu, Shan-Chwen Chang

**Affiliations:** 1 Department of Internal Medicine, National Taiwan University Hospital, Taipei, Taiwan; 2 Department of Medical Research, National Taiwan University Hospital, Taipei, Taiwan; 3 National Institute of Infectious Diseases and Vaccinology, National Health Research Institutes, Zhunan, Taiwan; 4 Centers for Disease Control, Taipei, Taiwan; 5 Graduate Institute of Epidemiology and Preventive Medicine, College of Public Health, National Taiwan University, Taipei, Taiwan; 6 Graduate Institute of Clinical Pharmacy, College of Medicine, National Taiwan University, Taipei, Taiwan; The University of Tokyo, JAPAN

## Abstract

A total of 1135 carbapenem-resistant (nonsusceptible) *Enterobacteriaceae* (CRE) isolates were recovered between November 2010 and July 2012 (517 from 2010-2011 and 618 from 2012) from 4 hospitals in Taiwan. Carbapenemase-producing *Enterobacteriaceae* (CPE) comprised 5.0% (57 isolates), including 17 KPC-2 (16 *Klebsiella pneumoniae* and 1 *Escherichia coli*), 1 NDM-1 (*K*. *oxytoca*), 37 IMP-8 (26 *Enterobacter cloacae*, 4 *Citrobacter freundii*, 4 *Raoultella planticola*, 1 *K*. *pneumoniae*, 1 *E*. *coli* and 1 *K*. *oxytoca*), and 2 VIM-1 (1 *E*. *cloacae*, 1 *E*. *coli*). The KPC-2-positive *K*. *pneumoniae* were highly clonal even in isolates from different hospitals, and all were ST11. IMP-8 positive *E*. *cloacae* from the same hospitals showed higher similarity in PFGE pattern than those from different hospitals. A total of 518 CRE isolates (45.6%) were positive for *bla*_ESBL_, while 704 (62.0%) isolates were *bla*_AmpC_-positive, 382 (33.6% overall) of which carried both *bla*_ESBL_ and *bla*_AmpC_. CTX-M (414, 80.0%) was the most common *bla*_ESBL_, while DHA (497, 70.6%) and CMY (157, 22.3%) were the most common *bla*_AmpC_. Co-carriage of *bla*_ESBL_ and *bla*_AmpC_ was detected in 31 (54.4%) and 15 (26.3%) of the 57 CPE, respectively. KPC-2 was the most common carbapenemase detected in *K*. *pneumoniae* (2.8%), while IMP-8 was the most common in *E*. *cloacae* (9.7%). All KPC-2-positive CRE were resistant to all three tested carbapenems. However, fourteen of the 37 IMP-8-positive CRE were susceptible to both imipenem and meropenem in vitro. Intra- and inter-hospital spread of KPC-2-producing *K*. *pneumoniae* and IMP-8-producing *E*. *cloacae* likely occurred. Although the prevalence of CPE is still low, careful monitoring is urgently needed. Non-susceptibility to ertapenem might need to be considered as one criterion of definition for CRE in areas where IMP type carbapenemase is prevalent.

## Introduction

Bacteria belonging to *Enterobacteriaceae*, such as *Escherichia coli*, *Klebsiella pneumoniae*, *Enterobacter* spp., *Citrobacter* spp, *Serratia* spp, *Proteus* spp, and *Morganella*, are all important human pathogens [1]. They cause a wide array of diseases including urinary tract, respiratory tract, bloodstream, intra-abdominal, and skin and soft tissue infections [1]. Treatment of infections caused by these bacteria has become challenging particularly those with increasing resistance to extended spectrum β-lactams due to expression of extended-spectrum β-lactamase (ESBL) and/or AmpC β-lactamase [2,3]. In addition to being resistant to commonly used extended spectrum β-lactams [4], these isolates are usually resistant to other classes of antibiotics including fluoroquinolones and aminoglycosides at the same time [5]. Therefore, carbapenems have been the major last agent of choice for treating infections caused by these multidrug-resistant isolates [2,5,6].

However, carbapenem resistance among *Enterobacteriaceae* has increased gradually over the years in different regions [3,7–14]. The emergence of carbapenem-resistant *Enterobacteriaceae* (CRE) is worrisome because treatment options are very limited [3,9,15]. The mechanisms of carbapenem resistance among *Enterobacteriaceae* include production of ESBL and/or AmpC enzymes in combination with loss of outer membrane protein or up-regulation of efflux pump, and secretion of carbapenemases. Among the carbapenemases found in *Enterobacteriaceae*, *K*. *pneumoniae* carbapenemase (KPC) and New-Delhi metallo-β-lactamase1 (NDM-1) have been most noteworthy because they can confer high-level carbapenem resistance and because genes encoding these enzymes are mostly plasmid-borne and have spread between different species of *Enterobacteriaceae* worldwide [3,9,15].

The prevalence of CRE in Taiwan, although remained low, has increased in recent years [16–20]. These CRE isolates may be resistant to one or all of the three carbapenem agents, ertapenem, imipenem, and/or meropenem depending on the agents tested by the clinical microbiology laboratories in Taiwan. Prior studies from Taiwan limited their scope in single bacterial species or single infection syndrome, which might not demonstrate the whole picture of CRE in Taiwan. The present study aimed to increase our understanding on the epidemiology of CRE in Taiwan by studying different species of CRE isolated from various clinical specimens over a 2-year period from 4 hospitals. The objectives of the study were to investigate the drug susceptibilities of CRE to commonly used broad-spectrum antibiotics and to determine the distribution of carbapenemases as well as ESBLs and AmpC β-lactamases in these CREs. Since carbapenemase-producing *Enterobacteriaceae* (CPE) is the most worrisome threat, we performed further molecular characterizations on the CPE isolates to study their clonal relatedness and genetic background. The possible effects of ESBLs and/or AmpC β-lactamase co-carriage on the carbapenem minimum inhibitory concentrations (MICs) of CPE were also determined.

## Materials and Methods

### Isolates

Between November 2010 and July 2012, non-duplicate *Enterobacteriaceae* (CRE) isolates nonsusceptible to ertapenem, imipenem, and/or meropenem recovered from adult patients at 2 major medical centers [National Taiwan University Hospital (NTUH) and Far Eastern Memorial Hospital (FEMH)] and 2 regional hospitals (NTUH Hsin-Chu Branch and NTUH Yun-Lin Branch) were collected. The 2 medical centers are located in northern Taiwan; while the 2 regional hospitals are located in central and southern Taiwan, respectively. Isolates determined to be nonsusceptible to ertapenem, imipenem, and/or meropenem by the participating hospitals were collected and then subjected to antimicrobial susceptibility test described below. Only those confirmed to be carbapenem-nonsusceptible (either of ertapenem, imipenem, or meropenem) based on the 2012 Clinical and Laboratory Standard Institutes (CLSI) criteria [ertapenem (S, ≤ 0.5; I,1; R, ≥ 2 μg/mL), imipenem (S ≤ 1; I, 2; R, ≥ 4 μg/mL), and/or meropenem (S, ≤ 1; I, 2; R, ≥ 4 μg/mL) were considered CRE [21]. Isolates were stored at -70°C in 20% glycerol containing Trypticase soy broth. The study was approved by the NTUH Institute Review Boards (IRB) (IRB_201009053R, for NTUH and its 2 branch hospitals) and FEMH IRB (FEMH-IRB-10000-E1, for FEMH). The IRBs waived the need for informed consents (both written and oral) from source patients of the enrolled bacterial isolates because this was an observational study, involved very minimal risk to the source patients, did not include intentional deception, and did not involve sensitive populations or topics; this waiver does not adversely affect the rights and welfare of the source patients.

### Antimicrobial susceptibility testing (AST)

All isolates were subcultured twice prior to AST testing. Minimum inhibitory concentrations (MICs) were determined by agar dilution method following the CLSI guidelines [22]. The β-lactam agents tested included cefepime, cefotaxime, flomoxef, and three carbapenems ertapenem, imipenem, and meropenem. Non β-lactam agents tested included amikacin, ciprofloxacin, colistin and tigecycline. All agents were tested at 0.03 to 128 μg/mL using in-house prepared panels. Quality control strains included *Escherichia coli* ATCC 25922 and *Pseudomonas aeruginosa* ATCC 27853. Interpretive criteria were based on CLIS breakpoints where available [21]. The breakpoints proposed by European Committee on Antimicrobial Susceptibilities Testing (EUCAST) were used for colistin and tigecycline [23]. The flomoxef breakpoint was based on Liao et al (S, ≤ 8; I,16–32; R, ≥ 64 μg/mL) [24].

### DNA extraction

For each test isolate, 3 to 5 colonies were lightly picked from fresh overnight culture plate to suspend in 150 μl AE buffer [50 mM sodium acetate (pH 5.2) and 10 mM EDTA (pH 8.0)]. The suspension was heated at 95°C for 15 min, then centrifuged at 1000 g for 10 min to remove cellular debris, after which 100 μl of the supernatant was transferred to a new vial The DNA preparation was stored at -20°C and used as template for subsequent amplifications.

### Detection of β-lactamase

Multiplex PCR was used to determine the presence of the genes encoding AmpC, ESBL, and carbapenemases following previously published protocols [7,25–27]. All carbapenemase PCR positive amplicons were sequenced to check for amplicon specificity.

### Molecular typing

Isolates positive for carbapenemases were subject to pulsed field gel electrophoresis (PFGE) and multi-locus sequence typing (MLST) following previously published protocols and information from the MLST website (http://www.pasteur.fr/recherche/genopole/PF8/mlst/EColi.html) [28]. For interpretation of the PFGE banding patterns, unweighted-pair group method using average linkages (UPGMA) dendrograms were constructed from the original data. Isolates that exhibited similarity of ≥ 80% of their banding patterns were considered to belong to the same strains (pulsotypes). Replicon typing was performed using primers and protocols previously published [29].

### Data analysis

For analysis of susceptibility rates by different stratification, the WHONET software was used [30]. Duplicate isolates were excluded from analysis. The definition of duplicate isolate was isolation of the same species from the same patient within 30 days. Statistical analysis was performed using Epi Info 6.04 (CDC, Atlanta, GA). The χ^2^ test was used to determine significant differences in frequencies of susceptibility. A *p* < 0.05 was considered to be statistically significant.

## Results

### Isolates

Among the 1295 isolates collected with their initial reports as carbapenem-nonsusceptible from the four hospitals during the study period, 160 were excluded from subsequent analysis because the MICs of the three carbapenem agents tested by agar dilution were in the susceptible range based on the 2012 CLSI criteria [21]. The remaining 1135 CRE isolates included 517 from 2010–2011 and 618 from 2012. The most common species were *K*. *pneumoniae* (CR-kpn) (n = 577, 50.8%), followed by *E*. *cloacae* complex (CR-ecl) (n = 267, 23.5%), *E*. *coli* (CR-eco) (n = 145, 12.8%), *E*. *aerogenes* (CR-eae) (n = 88, 7.8%), and *C*. *freundii* (CR-cfr) (20, 1.8%), together they comprised 94.9% of the isolates studied. The majority of the isolates were from respiratory (407, 35.9%) and urine (405, 35.7%) specimens. However, the specimen distribution varied among different species (Table 1).

**Table 1:** Species and specimen distribution of 1135 carbapenem-nonsusceptible *Enterobacteriaceae* recovered from 4 hospitals in Taiwan during November 2010 and July 2012.

	Specimen type [n (%)]^a^						
Species	Abscess & Drainage	Blood	Respiratory	Urine	CVP	Other^c^	Total
*Klebsiella pneumoniae*	49 (8.5)	41 (7.1)	259 (44.9)	190 (32.9)	19 (3.3)	19 (3.3)	577
*Enterobacter cloacae* complex^b^	58 (21.7)	16 (6.0)	85 (31.8)	85 (31.8)	5 (1.9)	18 (6.7)	267
*Escherichia coli*	25 (17.2)	14 (9.7)	19 (13.1)	77 (53.1)	0	10 (6.9)	145
*Enterobacter aerogenes*	16 (18.2)	13 (14.8)	28 (32.0)	24 (27.3)	1 (1.1)	6 (6.8)	88
*Citrobacter freundii*	4 (20.0)	1 (5.0)	7 (35.0)	7 (35.0)	1 (5.0)	0	20
*Serratia marcescens*	0	1	6	6	0	1	14
*Morganella morgannii*	1	1	0	2	0	1	5
*Providencia stuartii*	0	0	0	5	0	0	5
*Klebsiella oxytoca*	0	0	1	1	0	0	2
*Raoultella (Klebsiella) planticola*	0	1	2	1	0	0	4
*Providencia rettgeri*	0	0	0	3	0	0	3
*Citrobacter diversus*	1	0	0	2	0	0	3
*Citrobacter koseri*	0	0	0	2	0	0	2
Total	154 (13.6)	88 (7.8)	407 (35.9)	405 (35.7)	26 (2.3)	55 (4.8)	1135

^*a*^ % calculated for species with > = 20 isolates only.

^*b*^ *Enterobacter cloacae* complex included 265 *E*. *cloacae* and 2 *E*. *asburiae*.

^c^ Including 2 anal swabs, 20 ascites, 10 bile, 2 body fluids (source unknown), 1 CSF, 5 double lumen, 2 each of pleural effusion and throat swab, 7 tissue, and 1 each of endometrium, H-V tip, prostate, and nasal specimen.

### Antimicrobial susceptibility of CRE and the predominant species of CRE isolates

The activities of the 10 broad-spectrum antimicrobial agents against all 1135 CRE isolates as well as against the five predominant species of CRE are presented in Table 2 and S1 file. Carbapenem-resistance was observed mostly in ertapenem (98.5%), with 72.8% and 89.2% of the isolates remaining susceptible (S) to imipenem and meropenem, respectively.

Nearly all isolates were resistant to cefotaxime (1.3% S overall). Similar high rates of resistance were observed for cephamycin flomoxef. Overall, 50.7% of the isolates were susceptible to cefepime but differed significantly among the 5 predominant species, ranging from 31.2% in CR-kpn to 88.6% in CR-eae (Table 2). Susceptibility to amikacin was 81.5% overall, but was significantly lower in CR-kpn (67.4%) and higher in CR-eco (96.6%) and CR-ecl (97.8%) and CR-eae (100%) (*p* < 0.01). In contrast, susceptibility to ciprofloxacin was low at 34.4% over all, and was again significantly lower in CR-kpn (11.3%) and CR-eco (21.4%) compared to those of CR-eae (71.6%) and CR-ecl (74.9%). Resistance to colistin was 8.6% overall and 7.5% (84/1123) if the isolates with intrinsic resistance (*Morganella* and *Providencia*) were excluded. Of note, resistance to tigecycline was 9.2% overall, but was higher in CR-ecl (15.7%) and CR-eae (18.2%) and lower in CR-eco (0%) and CR-kpn (6.8%) (Table 2).

**Table 2:** In vitro activities of 10 antimicrobial agents against all carbapenem-nonsusceptible *Enterobacteriaceae* (all species combined), *K*. *pneumoniae*, *E*. *coli*, *E*. *cloacae*, *E*. *aerogenes*, and *C*. *freundii*.

Antimicrobial agents	Susceptibilities			Detailed MIC		
	%R	%I	%S	MIC_50_	MIC_90_	Range
All isolates (n = 1135)^a^						
Ertapenem	70.2	28.3	1.5	2	16	0.5 - > 128
Imipenem	14.4	12.9	72.8	1	4	0.06–128
Meropenem	8.5	2.4	89.2	0.125	2	≤ 0.03 - > 128
Cefotaxime	97.2	1.5	1.3	128	> 128	≤ 0.03 - > 128
Flomoxef	90.8	5.2	4.1	> 128	> 128	0.25 - > 128
Cefepime	43.7	5.7	50.7	8	128	≤ 0.03 - > 128
Amikacin	17.4	1.1	81.5	2	> 128	0.5 - > 128
Ciprofloxacin	61.9	3.8	34.4	32	> 128	≤ 0.03 - > 128
Colistin^b^	8.6	-	91.4	0.5	1	0.25 - > 128
Tigecycline	9.2	-	90.8	1	1	0.06–16
*K*. *pneumoniae* (n = 577)						
Ertapenem	76.8	22.4	0.9	2	64	0.5 - > 128
Imipenem	14.9	11.4	73.7	1	8	0.125–128
Meropenem	10.6	1.9	87.5	0.125	4	≤ 0.03 - > 128
Cefotaxime	98.3	1.6	0.2	> 128	> 128	1 - > 128
Flomoxef	89.3	7.3	3.5	128	> 128	0.25 - > 128
Cefepime	63.3	5.6	31.2	32	> 128	0.06 - > 128
Amikacin	31.1	1.4	67.4	2	> 128	0.5 - > 128
Ciprofloxacin	86.7	2.1	11.3	128	> 128	≤ 0.03 - > 128
Colistin	4.0	-	96.0	0.5	1	0.25 - > 128
Tigecycline	6.8	-	93.2	1	1	0.125–16
*E*. *cloacae* complex (n = 267)						
Ertapenem	59.6	38.2	2.3	2	8	0.5–64
Imipenem	7.5	13.9	78.7	0.5	2	0.25–64
Meropenem	3.0	1.9	95.1	0.125	0.5	≤ 0.03 - > 128
Cefotaxime	96.3	1.5	2.3	128	> 128	≤ 0.03 - > 128
Flomoxef	95.9	2.6	1.5	> 128	> 128	2 - > 128
Cefepime	15.4	7.1	77.5	2	32	≤ 0.03 - > 128
Amikacin	1.5	0.8	97.8	1	4	0.5 - > 128
Ciprofloxacin	19.5	5.6	74.9	0.125	16	≤ 0.03 - > 128
Colistin	18.0	-	82.0	1	128	0.25 - > 128
Tigecycline	15.7	-	84.3	1	4	0.125–16
*E*. *coli* (n = 145)						
Ertapenem	78.6	21.4	0	2	16	1 - > 128
Imipenem	13.1	8.3	78.6	0.5	4	0.06–64
Meropenem	6.9	2.1	91.0	0.125	1	≤ 0.03–32
Cefotaxime	100	0	0	> 128	> 128	4 - > 128
Flomoxef	86.9	2.1	11	> 128	> 128	1 - > 128
Cefepime	44.8	4.8	50.3	8	> 128	0.06 - > 128
Amikacin	3.5	0	96.6	2	8	1 - > 128
Ciprofloxacin	75.9	2.8	21.4	64	> 128	≤ 0.03 - > 128
Colistin	0.7	-	99.3	0.5	1	0.25–32
Tigecycline	0	-	100	0.5	1	0.06–1
*E*. *aerogenes* (n = 88)						
Ertapenem	44.3	53.4	2.3	1	32	0.5–128
Imipenem	17.1	20.5	62.5	1	8	0.25–16
Meropenem	9.1	2.3	88.6	0.125	2	0.03–8
Cefotaxime	92.1	2.3	5.7	64	> 128	0.125 - > 128
Flomoxef	97.7	1.1	1.1	128	> 128	8 - > 128
Cefepime	10.2	1.1	88.6	1	32	0.06 - > 128
Amikacin	0	0	100	2	4	1–16
Ciprofloxacin	19.3	9.1	71.6	0.125	8	0.03 - > 128
Colistin	3.4	-	96.6	1	1	0.25–8
Tigecycline	18.2	-	81.8	1	4	0.25–16
*C*. *freundii* (n = 20)						
Ertapenem	50	50	0	1	8	1–64
Imipenem	20	25	55	1	8	0.25–16
Meropenem	10	0	90	0.125	1	0.03–8
Cefotaxime	100	0	0	128	> 128	16 - > 128
Flomoxef	90	10	0	> 128	> 128	16 - > 128
Cefepime	20	5	75	2	32	0.125–128
Amikacin	15	5	80	2	64	1 - > 128
Ciprofloxacin	25	5	70	0.5	8	0.03–64
Colistin	10	-	90	1	2	0.25 - > 128
Tigecycline	10	-	90	0.5	2	0.125–16

^a^ Including all species listed in Table 1.

^b^Including *Morganella* and *Providencia* isolates, which are intrinsically resistant to colistin

### Prevalence of ESBL and AmpC β-lactamase

All 1135 CRE isolates were tested for the presence of genes encoding the most common ESBL and AmpC β-lactamases (*bla*_ESBL_ and *bla*_AmpC_) regardless of carbapenemase status. Overall, 518 (45.6%) and 704 (62.0%) isolates were positive for *bla*_ESBL_ and *bla*_AmpC_, respectively. Of alarming is that 382 (33.6%) isolates carried both ESBL and AmpC, and carriage of multiple ESBL and/or AmpC was quite common. Genes encoding CTX-M (414), SHV (148, including 45 positive for both) accounted for 71.2% and 28.6% of the *bla*_ESBL_ detected, respectively. Genes encoding DHA (497) and CMY (157, including 13 carrying both) accounted for 70.6% and 22.3% of the *bla*_AmpC_ detected, respectively. Two other *bla*_AmpC_ not as commonly reported, MIR (36) and ACT (26), also accounted for 5.1% and 3.7% of the *bla*_AmpC_, respectively (Table 3).

**Table 3:** ESBL, AmpC β-lactamases, and carbapenemase genes detected in carbapenem-non-susceptible *Enterobacteriaceae*.

Species (no. of tested)	Gene type and no. of isolates positive		Combination of *bla*_ESBL_ / *bla*_AmpC_ (n, %)				
	*bla*_ESBL_	*bla*_AmpC_	+ / -	- / +	+ / +	- / -	Major combination
*K*. *pneumoniae* (577)	CTX-M (291), SHV (56), CTX-M+SHV (37)	DHA (458), CMY (17),CMY+ DHA (12), MIR (2)	64 (11.1)	169 (29.2)	320 (55.4)	24 (4.2)	CTX-M & DHA (85.6%, 274/320)^a^
*E*. *cloacae* (267)	SHV (43), CTX-M + SHV(3), CTX-M (2)	MIR (34), ACT (25), DHA (9), CMY (2), DHA+MIR (1), DHA+CMY (1)	39 (14.6)	63 (23.6)	9 (3.4)	156 (58.4)	SHV & ACT (77.8%, 7/9)
*E*. *coli* (145)	CTX-M (66), CTX-M+SHV (1)	CMY (109), DHA (6), ACT (1)	20 (13.8)	69 (47.6)	47 (32.4)	9 (6.2)	CTX-M & CMY (93.6%, 44/47)
*E*. *aerogenes* (88)	CTX-M (2), SHV (1)	DHA (2)	3 (3.4)	2 (2.3)	0	83 (94.3)	-
*C*. *freundii* (20)	CTX-M (2), SHV (2), both (1)	CMY (10), DHA (1)	0	6 (30.0)	5 (25.0)	9 (45.0)	SHV & CMY (2)
*S*. *marcescens* (14)	CTX-M (2), SHV (1)	DHA (2)	3 (21.4)	2 (14.3)	0	9 (64.3)	-
*M*. *morgannii* (5)	0	DHA (5)	0	5 (100)	0	0	
*P*. *stuartii* (5)	CTX-M (1)	CMY (2), DHA(1)	1 (20)	3 (60)	0	1 (20)	
*C*. *diversus* (3)	CTX-M (1), CTX-M+SHV (1)	CMY (2)	1 (33.3)	1 (33.3)	1 (33.3)	0	CTX-M & CMY (1)
*K*. *oxytoca* (2)	CTX-M+SHV (1), SHV+OXY-5 (1)	0	2 (100)	0	0	0	-
*P*. *rettgeri* (3)	CTX-M (1)	CMY (1)	1 (33.3)	1 (33.3)	0	1 (33.3)	-
*R*. *planticola* (4)	0	0	0	0	0	4 (100)	-
*C*. *koseri* (2)	CTX-M (1)	CMY (1)	1 (50)	1 (50)	0	0	
ALL	CTX-M (369), CTX-M+SHV (45), SHV (103), SHV+OXY-5 (1)	DHA (484), CMY (144), MIR (36), ACT (26), CMY+DHA (13), DHA+MIR (1)	135 (11.9)	322 (28.4)	382 (33.7)	296 (26.1)	CTX-M+DHA (254), CTX-M+CMY (49), SHV+DHA(45)

^a^ % is no. of isolates with the combination/no. of isolates positive for both *bla*_ESBL_ and *bla*_AmpC_.

Except *Raoultella* (*Klebsiella*) *planticola*, *bla*_ESBL_ and/or *bla*_AmpC_ were detected in the other 12 species studied (Table 3). In CR-kpn, 66.1% and 84.6% were *bla*_ESBL_ and *bla*_AmpC_ positive, respectively, with 55.4% positive for both. CR-eco also had high rates of *bla*_ESBL_ and *bla*_AmpC_ co-carriage (32.4%). Although CTX-M *bla*_ESBL_ predominated in both CR-kpn (84.1%) and CR-eco (100%), the predominant *bla*_AmpC_ was DHA (96.1%) for CR-kpn, while CMY (94.0%) predominated in CR-eco. SHV was the predominant *bla*_ESBL_ in CR-ecl (95.8%) but several types of *bla*_AmpC_ were detected, with MIR, ACT, DHA being most common. Of noteworthy is that compared to the aforementioned three species, 94.3% of the CR-eae were negative for either *bla*_ESBL_ or *bla*_AmpC_. In CR-cfr, CMY *bla*_AmpC_ was detected in half of the 20 isolates studies, and 20% of them carried both *bla*_ESBL_ and *bla*_AmpC_ genes.

There were 276 (24.3% overall) CRE isolates that were negative for the ESBL, AmpC and carbapenemase genes investigated in this study. The majority (72.4%) had ertapenem MICs ≤ 2 μg/mL, the old CLSI susceptible breakpoint [31], while 43 (3.8% overall) had ertapenem MICs ≥ 8 μg/mL. Most of the 276 isolates were *Enterobacter* spp. (227 isolates) with the rest being *Citrobacter* (8), *E*. *coli* (8), *Klebsiella* (22), and *Providencia* (2).

### Antimicrobial susceptibilities and carbapenemase distribution of carbapenemase-producing *Enterobacteriaceae* (CPE)

CPE comprised 5.0% (57 isolates) of the 1135 CRE isolates studied, including 37, 17, 1, and 2 isolates positive for IMP-8, KPC-2, NDM-1, and VIM-1, respectively (Table 4). Among the 577 CR-kpn isolates, 18 were carbapenemase-positive, which included 16 KPC-2, and 1 each of IMP-8 and VIM-1. As to the 267 CR-ecl isolates, carbapenemase was detected in 10.1%, with 26 isolates positive for metallo-β-lactamase (MBL) IMP-8 and another positive for VIM-1. Two CR-eco isolates were carbapenemase-positive (1 IMP-8 and 1 KPC-2). Four CR-cfr isolates were positive for IMP-8. Among the 2 carbapenem-resistant *K*. *oxytoca* isolates, 1 was NDM-1-positive, and the other was IMP-8 positive. All 4 *R*. *planticola* isolates were IMP-8 positive (Table 4).

**Table 4:** Distribution of *bla*_carbapenemases_ among the 1135 CRE isolates.

Species (n)	N (%) positive for *bla*_carbapenemases_				
	IMP-8	KPC-2	NDM-1	VIM-2	All
*K*. *pneumoniae* (577)	1 (0.2)	16 (27.7)	0	1 (0.2)	18 (3.1)
*E*. *cloacae* (267)	26 (9.7)	0	0	1 (0.4)	27 (10.1)
*E*. *coli* (145)	1 (0.7)	1 (0.7)	0	0	2 (1.4)
*E*. *aerogenes* (88)	0	0	0	0	0
*C*. *freundii* (20)	4 (20)	0	0	0	4 (20)
*S*. *marcescens* (14)	0	0	0	0	0
*M*. *morgannii* (5)	0	0	0	0	0
*P*. *stuartii* (5)	0	0	0	0	0
*C*. *diversus* (3)	0	0	0	0	0
*K*. *oxytoca* (2)	1 (50)	0	1 (50)	0	2 (100)
*P*. *rettgeri* (3)	0	0	0	0	0
*R*. *planticola* (4)	4 (100)	0	0	0	4 (100)
*C*. *koseri* (2)	0	0	0	0	0
Total (1135)	37 (3.3)	17 (1.5)	1 (0.1)	2 (0.2)	57 (5.0)

All 17 KPC-2 positive isolates were highly and 100% resistant to all of the three tested carbapenems (ertapenem, imipenem, and meropenem) and extended spectrum β-lactams (cefotaxime, flomoxef, and cefepime) with high MICs (Table 5). Resistance to colistin and tigecycline was each 5.9%. In contrast, IMP-8-producers showed varied non-susceptibilities (intermediate or resistant) to ertapenem, imipenem, and meropenem, at 94.6%, 62.1%, and 13.5%, respectively; but no isolate was susceptible to all three carbapenems. Resistance to tigecycline was 27.0% but that of colistin was 5.4% among the IMP-8-producers.

**Table 5:** In vitro activities of 10 antimicrobial agents against *bla*_KPC-2_ and *bla*_IMP-8_ positive *Enterobacteriaceae*.^a^

Antimicrobial agent	*bla*_KPC-2_ positive isolates (n = 17)^b^						*bla*_IMP-8_ positive isolates (n = 37)^c^					
	%R	%I	%S	MIC_50_	MIC_90_	MIC Range	%R	%I	%S	MIC_50_	MIC_90_	MIC Range
**Carbapenem:**												
Ertapenem	100	0	0	> 128	> 128	32 - > 128	81.1	13.5	5.4	4	8	0.5–64
Imipenem	100	0	0	64	64	16–64	37.8	24.3	37.8	2	16	0.5–64
Meropenem	100	0	0	128	> 128	16 - > 128	2.7	10.8	86.5	0.5	2	0.125–4
**Cephalosporins:**												
Cefotaxime	100	0	0	> 128	> 128	128 - > 128	100	0	0	128	> 128	8 - > 128
Flomoxef	100	0	0	> 128	> 128	64 - > 128	81.1	13.5	5.4	> 128	> 128	8 - > 128
Cefepime	100	0	0	128	> 128	32 - > 128	48.6	18.9	32.4	16	128	0.25 - > 128
**Non-β-lactams:**												
Amikacin	17.6	5.9	76.5	2	> 128	1 - > 128	8.1	0	91.9	2	8	1 - > 128
Ciprofloxacin	94.1	0	5.9	128	> 128	0.5 - > 128	67.6	10.8	21.6	8	128	≤ 0.03 - > 128
Colistin	5.9	0	94.1	0.5	1	0.5 - > 16	5.4	0	94.6	1	1	0.5 - > 128
Tigecycline	5.9	0	94.1	1	1	0.25–8	27.0	0	73.0	1	4	0.25–8

^a^ Interpretive breakpoints are based on the 2012 CLSI criteria except colistin and tigecycline breakpoints for which the EUCAST breakpoints are used, while flomoxef breakpoints are based on Liao et al (JMII, 2006).

b including 16 *K*. *pneumoniae* and 1 *E*. *coli*.

c including 1 *K*. *pneumoniae*, 26 *E*. *cloacae*, 1 *E*. *coli*, 1 *K*. *oxytoca*, 4 *C*. *freundii* and 4 *R*. *planticola*.

### Clonal relationship of CPE

**Fig 1:**
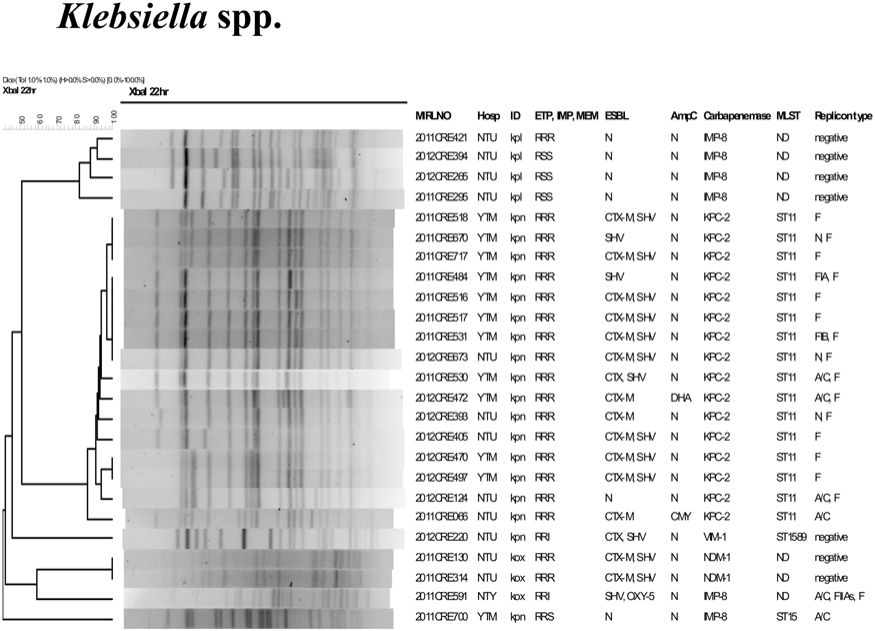
Dendrogram and molecular characterization of carbapenemase-positive *Klebsiella* spp. Abbreviation: kpn, *K*. *pneumoniae*, kox, *K*. *oxytoca*, kpl, *R*. *planticola*. Resistance to carbapenem: ETP, ertapenem; IMP, imipenem; MEM, meropenem. MLST: ST, sequence type; N, Negative; No PC No product; ND, not determined.

**Fig 2:**
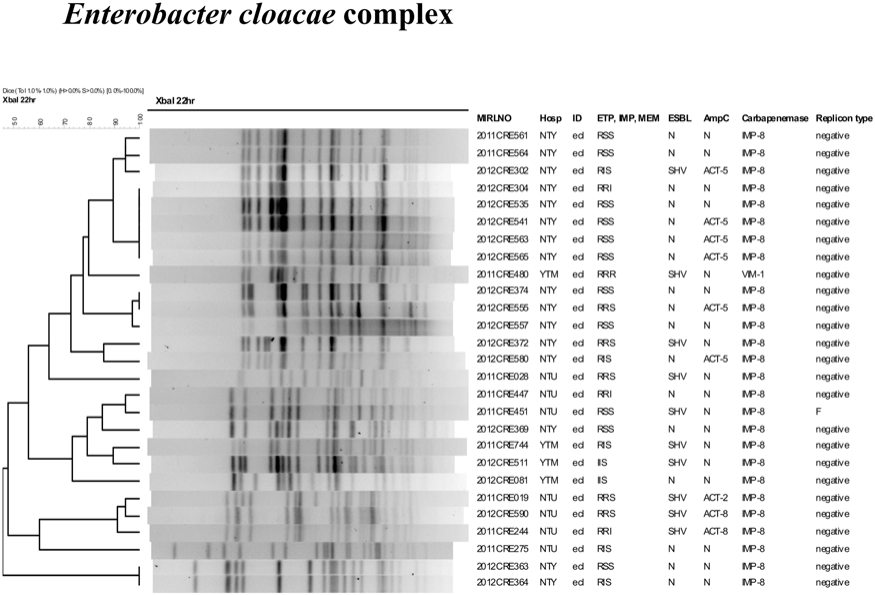
Dendrogram and molecular characterization of carbapenemase-positive *Enterobacter cloacae* complex (ecl) isolates.

**Fig 3:**
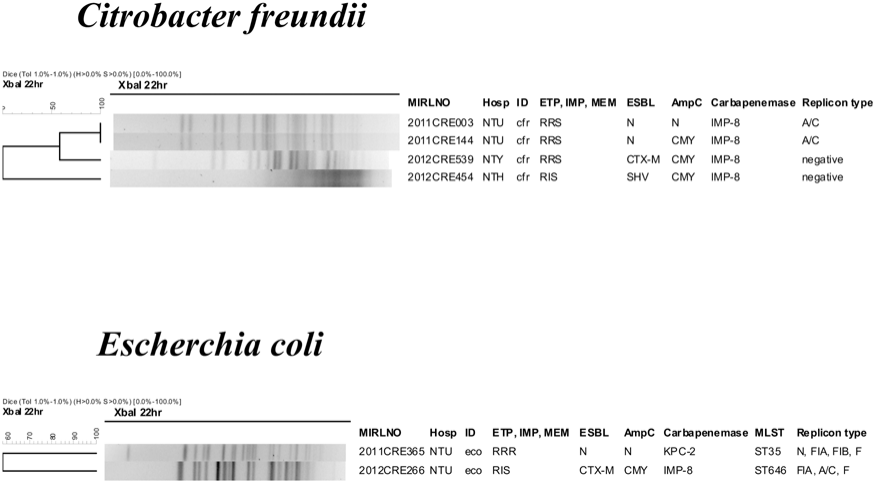
Dendrogram and molecular characterization of carbapenemase-positive *Citrobacter freundii* (A) and *Escherichia coli* (B).

The clonal relationships of the CPE are shown in Figs. 1–3. In Fig. 1, *K*. *pneumoniae*, *K*. *oxytoca*, and *R*. *planticola* are grouped together in the same dendrogram for comparison. The 16 KPC-2-producing *K*. *pneumoniae* are either indistinguishable or closely related and all are sequence type (ST) 11. However, some differences existed among these KPC-2 producers in the ESBL and AmpC they carried. In addition, although 7 of the 16 KPC-2-positive CRE-kpn isolates carried type F InC plasmid replicon group, other replicon types (A/C, FIA, FIB, and N) were also detected. Six isolates were also positive for more than one replicon types, indicating the existence of multiple plasmids. The IMP-8 and VIM-1 positive *K*. *pneumoniae* isolates are distinct from the KPC-2 isolates by PFGE. Their ST also differed from the KPC-2 isolates, with IMP-8 being ST15 while the VIM-1 isolate was ST1589, which is a new sequence type not previously reported.

The 4 IMP-8 positive *R*. *planticola* isolates shared >80% similarity in PFGE pattern to each other (Fig. 1). No plasmid replicon type could be determined. These 4 isolates were from 4 patients of the same hospital, 2 were recovered in 2011 and 2 in 2012, 2 were from sputum, 1 each from blood and urine.

The 26 IMP-8-positive CR-ecl isolates could be grouped into 4 main clusters (Fig. 2). For 3 of these clusters, isolates belonging to the same cluster came from the same hospital. In the largest cluster, eight isolates shared indistinguishable PFGE pattern but carried different combinations of ESBL and AmpC genes. Similar situation was noted in the other two clusters. For the other cluster (containing 3 isolates), isolates from the same hospital shared higher similarity than the isolate from another hospital.

Three of the 4 IMP-8 positive CR-cfr isolates had the same plasmid replicon type (A/C), two of which also had indistinguishable PFGE pattern (Fig. 3A). The two carbapenemase (KPC-2 and IMP-8)-positive *E*. *coli* had distinct PFGE pattern (Fig. 3B) and both also carried ESBL and AmpC genes. The sequence type of the KPC-2 and IMP-8 CR-eco isolates was ST35 and ST645, respectively. ST645 is a new ST not previously reported.

### ESBL and AmpC co-carriage, and carbapenem MIC in CPE

Co-carriage of *bla*_ESBL_ and *bla*_AmpC_ was detected in 31 (54.4%) and 15 (26.3%) of the 57 CPE isolates, respectively. Table 6 shows that the CPE isolates co-carrying *bla*_ESBL_ had the highest carbapenem MICs, followed by those positive for carbapenemase only. Co-carriage of *bla*_AmpC_ appeared to have no effect on carbapenem MICs in the CPE isolates. Of note, the MIC_50s_ of ertapenem, imipenem, meropenem were significantly lower in the CPE isolates co-carrying both *bla*_ESBL_ and *bla*_AmpC_ than those co-carrying *bla*_ESBL_ only. It needs to be pointed out that KPC-2 (13/22) predominated in the 22 CPE isolates co-carrying ESBL, while IMP-8 (7/9) predominated in the 9 CPE isolated co-carrying both *bla*_ESBL and_ *bla*_AmpC_ (Table 6 footnote).

**Table 6:** Carbapenem MICs (μg/mL) of 1135 carbapenem-nonsusceptible *Enterobacteriaceae* isolates with different ESBL, AmpC, and carbapenemase combination profiles.

No. of isolates	β-lactamase profile			Ertapenem			Imipenem			Meropenem		
	*bla*_ESBL_	*bla*_AmpC_	*bla*_Carb_ ^a^	MIC_50_	MIC_90_	MIC range	MIC_50_	MIC_90_	MIC range	MIC_50_	MIC_90_	MIC range
22	+^b^	-	+	>128	>128	0.5 - >128	32	64	1–64	64	>128	0.125 - >128
6	-	+^c^	+	4	8	1–8	1	8	0.5–8	0.5	1	0.5–1
9	+	+^d^	+	4	>128	1–128	8	64	2–64	1	128	0.5–128
20	-	-	+	4	32	0.5 - >128	1	16	0.5–64	0.5	4	0.25–64
113	+	-	-	2	16	1–128	0.5	2	0.06–32	0.25	4	0.06 - >128
316	-	+	-	2	8	0.5 - > 128	1	4	0.125–128	0.125	1	≤0.03–64
373	+	+	-	2	16	0.5 - >128	1	4	0.125–64	0.125	1	<0.03–32
276	-	-	-	2	8	0.5–128	0.5	4	0.125–64	0.125	1	≤0.03–16

^a^ *bla*_Carb_, carbapenemase. The distribution of the carbapenemases genes were: KPC-2 (13), IMP-8 (6), VIM-1 (2), and NDM-1 (1) among the 22 *bla*_ESBL_+ *bla*_AmpC_- *bla*_Carb_+ isolates; IMP-8 (6) among all 6 *bla*_ESBL_- *bla*_AmpC+_ *bla*_Carb_+ isolates; IMP-8 (7) and KPC-2 (2) among the 9 *bla*_ESBL_+ *bla*_AmpC_+ *bla*_Carb_+ isolates; and IMP-8 (18) and KPC-2 (2) among the 20 *bla*_ESBL_- *bla*_AmpC_- *bla*_Carb_+ isolates.

^b^ Co-carriage of *bla*_ESBL_ included CTX-M+SHV (12 isolates), SHV (8), CTX-M (1), SHV+OXY-5 (1).

^c^ Co-carriage of *bla*_AmpC_ included ACT (5), and CMY (1)

^d^ Co-carriage of *bla*_ESBL_ and *bla*_*AmpC*_ included SHV+ACT (4), CTX-M+CMY (3), SHV+CMY (1), CTX-M+DHA (1)

### Stratified analysis for species distribution, carbapenem susceptibilities, and distribution of carbapenemases by hospital types

When the 1135 CRE isolates were stratified by types of source hospital (medical centers vs. regional hospitals), we found that the distribution of species and carbapenemase differed but their carbapenem susceptibilities were similar (Table 7). Isolates from the two regional hospitals had lower proportions of CR-ecl, CR-eae, and CR-cfr, but a higher proportion of CR-kpn. However, all KPC-2, NDM-1, and VIM-1 positive CRE isolates were from the two medical centers, while IMP-8 was the only carbapenemase detected in CRE isolates from regional hospitals.

**Table 7:** Stratified analysis for species distribution, carbapenem susceptibilities, and distribution of carbapenemases by hospital types.

Parameters tested	Medical centers (n = 901)	Regional hospitals (n = 234)	*p* value
Distribution of species (n, %)			< 0.01
*K*. *pneumoniae*	421 (46.7%)	156 (66.7%)	
*E*. *cloacae*	237 (26.3%)	30 (12.8%)	
*E*. *coli*	110 (12.2%)	35 (15.0%)	
*E*. *aerogenes*	83 (9.2%)	5 (2.1%)	
*C*. *freundii*	18 (2.0%)	2 (0.8%)	
Carbapenem susceptibilities (S%)			
Ertapenem	1.8%	0.4%	0.22
Imipenem	71.8%	76.5%	0.15
Meropenem	88.5%	91.9%	0.13
Distribution of carbapenemases (n, %)			< 0.01
IMP-8	18 (47.4%)	19 (100%)	
KPC-2	17 (44.7%)	0	
NDM-1	1 (2.6%)	0	
VIM-1	2 (5.3%)	0	

## Discussion

The present study investigated the drug susceptibilities and relevant molecular characteristics of 1135 CRE including several common and important bacterial species from different source specimens, and found that the prevalence of carbapenemase-producing *Enterobacteriaceae* (CPE) among clinical CRE isolates was around 5.0% in Taiwan. The prevalence of CPE among hospitals with endemicity has been reported to range from 20% to 40% [32]. Prior studies from Taiwan demonstrated that the prevalence of carbapenemase production among imipenem-non-susceptible *E*. *coli* was 4% [33], and that of imipenem-non-susceptible *K*. *pneumoniae* increased from 6% to 22.3% between 2010 and 2012 [34]. Our study demonstrated that the proportion of CPE was lower (5.0% in overall *Enterobacteriaceae*, 1.4% in CR-eco, 3.1% in CR-kpn, and 10.1% in CR-ecl). However, if only isolates with non-susceptibility to imipenem were considered, the proportion of CPE among all species combined, *E*. *coli*, and *K*. *pneumoniae* would be 14.2% (44/309), 6.5% (2/31), and 11.8% (18/152), respectively. These rates are similar to those reported from Taiwan previously [32,34]. Therefore, comparison of CPE prevalence must take into account the carbapenem resistance criteria used.

The most prevalent carbapenemases among CPE isolates were KPC-2 and IMP-8; and NDM-type carbapenemase was found only in a single isolate. Sixteen of the 17 KPC-2-positive isolates were *K*. *pneumoniae*, and all were genetically indistinguishable or closely related, and belonged to ST11. This indicated that intra- and inter-hospital spread of KPC-2-positive *K*. *pneumoniae* occurred in Taiwan and echoed previous reports [34,35]. The first KPC-positive *K*. *pneumoniae* in Taiwan was found in 2010 [36]. The relatively short interval from its appearance to horizontal spread among hospitals [34,35] strongly suggested that KPC-positive *K*. *pneumoniae* isolates have a high potential to cause healthcare-associate outbreaks. In addition, the KPC-2-positive *K*. *pneumoniae* carried different types of plasmid and some carried more than one plasmid. Carriage of multiple plasmids has been found in carbapenem-resistant *Enterobacteriaceae* [29,37]. Co-carriage of different ESBL and different AmpC genes, alone or in combination, were also noted among the KPC-2 isolates. Taken together, these data indicated rapid evolution of the ST11 KPC-2 isolates with acquisition and spread of transferable resistance determinants in Taiwan.

One CR-eco isolate in the present study carried KPC-2 gene. A recent study from Taiwan also showed similar result [33]. However, the KPC-2-positive *E*. *coli* identified in the prior study belonged to ST410, not ST35 identified in our present study. This implies that the KPC gene might have been transmitted to multiple clones of *E*. *coli* in Taiwan. Whether KPC-2-positive *E*. *coli* would become more prevalent in Taiwan requires careful monitoring.

Of the 37 IMP-8-positive bacteria, those belonging to *E*. *cloacae* (26 isolates) and *R*. *planticola* (4 isolates) were most noteworthy. Studies from other countries demonstrated that IMP-positive *E*. *cloacae* have caused several outbreaks [32,39]. Although IMP-8 carbapenem-nonsusceptible *E*. *cloacae* has been reported in Taiwan since early 2000s [18,28], IMP-8 was detected in only 5.7% of the ertapenem-resistant *E*. *cloacae* [18]. However, we found that *E*. *cloacae* had the highest proportion (10.1%) of carbapenemase-producers among the three most common species of CRE, and 96.3% of carbapenemase-producing *E*. *cloacae* had IMP-8. This phenomenon has not been reported in Taiwan. We also found closely related IMP-8 *E*. *cloacae*, indicating possible intra- and inter-hospital spread. This likely contributed to the increased prevalence of IMP-8-positive *E*. *cloacae* in Taiwan. Careful monitoring of carbapenem susceptibilities and carriage of carbapenemase genes among *E*. *cloacae* clinical isolates should be implemented.

Although there were only four *R*. *planticola* isolates recovered in this study, all came from the same hospital, and all were positive for IMP-8 and genetically closely related. These results implied intra-hospital cross-transmission. The clinical importance of this species is still not well known, but cases of bacteremia, urinary tract infections, and necrotizing fasciitis were recently reported [40–42]. Although only a small number of carbapenem-resistant *R*. *planticola* isolates have been noted to date, further surveillance is necessary to elucidate its clinical impact.

The drug susceptibility profiles differed significantly between isolates carrying KPC-2 and IMP-8 carbapenemase genes. All 17 KPC-2 isolates were resistant to ertapenem, imipenem, and meropenem. However, they were highly susceptible to tigecycline (94.1%) and colistin (94.1%). The high carbapenem MICs are consistent with previous reports on KPC-2-producers [9,13]. In contrast, 5.4%, 37.8%, 86.5% of the IMP-8-positive isolates were susceptible to ertapenem, imipenem, and meropenem, respectively. In addition, although also highly susceptible to colistin (94.6%), the IMP-8-producers had lower susceptibility to tigecycline (73.0%). Therefore, tigecycline is not an adequate empirical antibiotic to treat infections caused by IMP-8-producing CRE in Taiwan.

It should be pointed out also that although tigecycline and colistin remained the two most active agents against the tested CRE (91.4% and 90.8%, respectively), susceptibility to tigecycline was lowest in *Enterobacter* spp., at 84.3% in CR-ecl and 81.8% in CR-eae. Susceptibility to colistin was also lowest (82.0%) in CR-ecl. Data on susceptibilities to tigecycline and/or colistin in carbapenem-resistant *Enterobacter* spp. are limited. However, a high resistance rate of up to 58.3% to tigecycline has been reported [43]. The mechanisms of tigecycline and/or colistin resistance in *Enterobacter* spp. warrant further investigation.

We defined CRE as *Enterobacteriaceae* isolates with non-susceptibility to ertapenem, imipenem, or meropenem in the present study. For the 1135 CRE isolates we tested, carbapenem resistance was mostly observed in ertapenem (98.5%), followed by imipenem (27.3%), and meropenem (10.9%). Similar results have been found by other studies [44]. More clinical studies should be done to determine whether infections caused by ertapenem-non-susceptible, but imipenem- or meropenem- susceptible *Enterobacteriaceae* could be effectively treated by imipenem or meropenem.

Of note, in the US Centers for Disease Control and Prevention (CDC) surveillance definition for CRE [45], it is suggested that CRE isolates should be non-susceptible to carbapenems other than ertapenem if the 2012 CLSI interpretive criteria were used [21,45]. Although the 2012 CLSI interpretive criteria were used in the present study, by the CDC CRE surveillance definition, 14 of the 37 IMP-8-positive isolates would not be considered as CRE. However, if non-susceptibility to ertapenem was considered, all would be recognized as CRE. Therefore, application of the surveillance definition for CRE proposed by CDC might be limited in some special situations, especially when *Enterobacteriaceae* isolates carrying IMP-type carbapenemases are prevalent. Therefore, whether non-susceptibility to ertapenem should be included as a criterion for CRE needs to be evaluated by each region depending on local carbapenemase prevalence.

Carriage of *bla*_ESBL_ and or *bla*_AmpC_ in combination with active efflux or porin loss has also been shown to be responsible for carbapenem-resistance in *Enterobacteriaceae* [46–48]. In the present study, 859 (75.7%) of the 1135 CRE isolates carried ESBL and AmpC genes alone or in combination. The two predominant types of ESBL genes were CTX-M (71.2%) and SHV (28.6%). The two predominant types of AmpC genes were DHA (70.6%) and CMY (22.3%). Prior studies have demonstrated that worldwide the most frequently reported ESBL and AmpC enzymes types were CTX-M and CMY, respectively [49]. However, the predominant type of AmpC enzymes in mainland China was DHA [49]. Taiwan neighbors and has frequent trades with mainland China. Therefore, the epidemiology in Taiwan might be similar to that in mainland China but differ from other countries.

There were 276 carbapenem-nonsusceptible isolates that were negative for the ESBL, AmpC and carbapenemase genes, and most were *Enterobacter* spp. (227 isolates). In a recent study of ertapenem-nonsusceptible *E*. *cloacae* (MICs 8 - > 256 μg/mL) in Taiwan, 24 of the 53 isolates studied were ESBL and AmpC enzyme negative but had either active efflux pump and/or loss of decreased outer membrane proteins [18]. Although the presence of other as yet unidentified β-lactamases in our isolates cannot be ruled out, decreased permeability due to outer membrane protein loss or active efflux pump likely also played a role.

Carbapenemase-producing isolates with co-carriage of ESBLs, and/or AmpC had different carbapenem MICs. Although this phenomenon might be due to varied synergistic effects between different β-lactamases, it might also be just a confounding event resulting from distributions of carbapenemases among isolates with or without co-carriage of ESBLs or AmpC β–lactamases since KPC-2 isolates had higher carbapenem MICs than those with IMP-8.

Difference in carbapenemase distribution between isolates from medical centers and regional hospitals was noted in the present study. CPE with IMP-type carbapenemases was reported in early 2000s and spread to many hospitals in different regions of Taiwan [18,38]. However, KPC-type CPEs did not appear in Taiwan until 2010 and were found mostly from hospitals in northern Taiwan [34–36]. The two medical centers in the present study are located in northern Taiwan whereas the two regional hospitals are located in central and southern Taiwan. This might be one reason accounting for the difference in carbapenemase distribution between the hospital types.

In conclusion, nearly all the CRE isolates in the present study were resistant to ertapenem but the majority remained susceptible to imipenem and meropenem. Carbapenemase was detected in 5.0% of the CRE isolates but the prevalence and carbapenemase differed by species. KPC-2 and IMP-8 were the two most common carbapenemases detected and their antibiograms differed. KPC-2 was mainly found in *K*. *pneumoniae* (2.8%), while IMP-8 was mostly found in *E*. *cloacae* (9.7%). KPC-2 was also detected in one *E*. *coli* isolates. Intra- and inter- hospital spread of KPC-2-producing ST11 *K*. *pneumoniae* and IMP-8-positive *E*. *cloacae* has occurred, which might lead to the increase of their prevalence. Analysis on plasmid replicons on the 16 KPC-2-producing ST11 *K*. *pneumoniae* revealed possible rapid evolution of these isolates with acquisition and spread of transferable resistance determinants. Of special concern is that non-susceptibility to ertapenem should be considered as a criterion for the surveillance definition of CRE in areas where IMP type carbapenemase is prevalent.

## Supporting Information

S1 File The raw data of 1135 carbapenem-resistant *Enterobacteriaceae* isolates. (PDF)
