# Supplementary material for: Carbapenem-Nonsusceptible Enterobacteriaceae in Taiwan
Source: PLoS One. 2015 Mar 20;10(3):e0121668. doi: 10.1371/journal.pone.0121668 (PMC4368706; doi:10.1371/journal.pone.0121668)
Supplement: S1 File — (PDF) [file pone.0121668.s001.pdf]

S1. The raw data of 1135 carbapenem-resistant *Enterobacteriaceae* isolates

CDCNO	Special_I	Species_	Hosp	ETP	ERT_C20	ETP__C2(	ETP_EU	IMP
2011CRE	E581	C. freunc	NTU	2	R	R	R	2
2011CRE	E582	C. freunc	NTU	1	I	R	I	2
2011CRE	E583	C. freunc	NTU	2	R	R	R	16
2011CRE	E584	E. cloaca	NTU	1	I	R	I	1
2011CRE	E585	E. cloaca	NTU	0.5	S	I	S	2
2011CRE	E586	E. cloaca	NTU	1	I	R	I	1
2011CRE	E587	E. cloaca	NTU	2	R	R	R	2
2011CRE	E588	E. aeroge	NTU	8	R	R	R	8
2011CRE	E590	E. aeroge	NTU	2	R	R	R	2
2011CRE	E591	E. aeroge	NTU	0.5	S	I	S	2
2011CRE	E592	E. aeroge	NTU	0.5	S	I	S	2
2011CRE	E593	E. aeroge	NTU	2	R	R	R	2
2011CRE	E594	E. aeroge	NTU	1	I	R	I	1
2011CRE	E595	E. aeroge	NTU	1	I	R	I	1
2011CRE	E596	E. aeroge	NTU	2	R	R	R	1
2011CRE	E597	E. cloaca	NTU	2	R	R	R	1
2011CRE	E598	E. cloaca	NTU	2	R	R	R	2
2011CRE	E599	E. cloaca	NTU	2	R	R	R	32
2011CRE	E600	E. cloaca	NTU	1	I	R	I	1
2011CRE	E601	E. cloaca	NTU	1	I	R	I	2
2011CRE	E602	E. cloaca	NTU	4	R	R	R	2
2011CRE	E603	E. cloaca	NTU	0.5	S	I	S	2
2011CRE	E604	E. cloaca	NTU	2	R	R	R	8
2011CRE	E605	E. cloaca	NTU	2	R	R	R	1
2011CRE	E606	E. cloaca	NTU	2	R	R	R	1
2011CRE	E607	E. cloaca	NTU	1	I	R	I	1
2011CRE	E608	E. cloaca	NTU	1	I	R	I	4
2011CRE	E609	E. cloaca	NTU	1	I	R	I	1
2011CRE	E612	E. cloaca	NTU	1	I	R	I	2
2011CRE	E614	E. coli	NTU	4	R	R	R	0.5
2011CRE	E615	E. coli	NTU	1	I	R	I	1
2011CRE	E616	E. coli	NTU	1	I	R	I	1
2011CRE	E617	E. coli	NTU	1	I	R	I	1
2011CRE	E618	E. coli	NTU	4	R	R	R	4
2011CRE	E619	E. coli	NTU	2	R	R	R	2
2011CRE	E621	E. coli	NTU	4	R	R	R	2
2011CRE	E622	E. coli	NTU	16	R	R	R	4
2011CRE	E623	E. coli	NTU	4	R	R	R	2
2011CRE	E624	E. cloaca	NTU	1	I	R	I	1
2011CRE	E625	E. coli	NTU	8	R	R	R	2
2011CRE	E626	E. coli	NTU	8	R	R	R	4
2011CRE	E627	K. pneun	NTU	1	I	R	I	8
2011CRE	E628	K. pneun	NTU	0.5	S	I	S	2
2011CRE	E629	K. pneun	NTU	1	I	R	I	1
2011CRE	E630	K. pneun	NTU	2	R	R	R	4

2011CRE	E631	K. pneum	NTU	1	I	R	I	2
2011CRE	E632	K. pneum	NTU	1	I	R	I	1
2011CRE	E633	K. pneum	NTU	1	I	R	I	1
2011CRE	E634	K. pneum	NTU	2	R	R	R	4
2011CRE	E635	K. pneum	NTU	1	I	R	I	2
2011CRE	E636	K. pneum	NTU	32	R	R	R	8
2011CRE	E637	K. pneum	NTU	0.5	S	I	S	2
2011CRE	E638	K. pneum	NTU	4	R	R	R	4
2011CRE	E639	K. pneum	NTU	2	R	R	R	4
2011CRE	E640	K. pneum	NTU	0.5	S	I	S	8
2011CRE	E641	K. pneum	NTU	4	R	R	R	4
2011CRE	E642	K. pneum	NTU	8	R	R	R	8
2011CRE	E643	K. pneum	NTU	2	R	R	R	1
2011CRE	E644	K. pneum	NTU	2	R	R	R	2
2011CRE	E646	K. pneum	NTU	>128	R	R	R	32
2011CRE	E651	E. aeroge	NTU	1	I	R	R	2
2011CRE	E652	E. aeroge	NTU	128	R	R	R	16
2011CRE	E656	E. aeroge	NTU	1	I	R	R	0.5
2011CRE	E657	E. cloaca	NTU	1	I	R	R	0.25
2011CRE	E658	E. cloaca	NTU	1	I	R	R	0.5
2011CRE	E659	E. cloaca	NTU	1	I	R	R	0.5
2011CRE	E660	E. cloaca	NTU	1	I	R	R	0.5
2011CRE	E661	E. cloaca	NTU	8	R	R	R	2
2011CRE	E663	E. cloaca	NTU	2	R	R	R	0.5
2011CRE	E664	E. cloaca	NTU	8	R	R	R	1
2011CRE	E665	E. cloaca	NTU	1	I	R	R	0.5
2011CRE	E666	E. cloaca	NTU	1	I	R	R	0.25
2011CRE	E667	E. cloaca	NTU	1	I	R	R	0.5
2011CRE	E668	E. cloaca	NTU	2	R	R	R	0.25
2011CRE	E669	E. cloaca	NTU	1	I	R	R	0.25
2011CRE	E670	E. coli	NTU	4	R	R	R	0.5
2011CRE	E671	E. coli	NTU	1	I	R	R	0.125
2011CRE	E672	E. coli	NTU	4	R	R	R	0.125
2011CRE	E675	E. coli	NTU	8	R	R	R	1
2011CRE	E676	E. coli	NTU	8	R	R	R	2
2011CRE	E678	K. pneum	NTU	2	R	R	R	0.125
2011CRE	E680	K. pneum	NTU	1	I	R	R	0.25
2011CRE	E681	K. pneum	NTU	1	I	R	R	1
2011CRE	E683	K. pneum	NTU	2	R	R	R	0.5
2011CRE	E685	K. pneum	NTU	1	I	R	R	1
2011CRE	E686	K. pneum	NTU	2	R	R	R	0.5
2011CRE	E687	K. pneum	NTU	16	R	R	R	1
2011CRE	E689	K. pneum	NTU	128	R	R	R	64
2011CRE	E692	K. pneum	NTU	2	R	R	R	1
2011CRE	E694	K. pneum	NTU	4	R	R	R	2
2011CRE	E695	K. pneum	NTU	128	R	R	R	32

2011CRE	E697	K. pneun	NTU	2	R	R	R	0.25
2011CRE	E700	K. pneun	NTU	1	I	R	R	1
2011CRE	E701	K. pneun	NTU	1	I	R	R	1
2011CRE	E704	C. koseri	NTU	1	I	R	R	0.25
2011CRE	E705	E. aeroge	NTU	1	I	R	R	0.5
2011CRE	E706	E. aeroge	NTU	1	I	R	R	1
2011CRE	E710	E. cloaca	NTU	1	I	R	R	0.5
2011CRE	E711	E. cloaca	NTU	1	I	R	R	2
2011CRE	E713	E. coli	NTU	2	R	R	R	0.25
2011CRE	E715	E. coli	NTU	2	R	R	R	1
2011CRE	E718	K. oxytoc	NTU	64	R	R	R	64
2011CRE	E719	K. pneun	NTU	2	R	R	R	1
2011CRE	E720	K. pneun	NTU	4	R	R	R	0.5
2011CRE	E721	K. pneun	NTU	2	R	R	R	0.25
2011CRE	E723	K. pneun	NTU	32	R	R	R	4
2011CRE	E724	K. pneun	NTU	2	R	R	R	0.5
2011CRE	E725	K. pneun	NTU	1	I	R	R	1
2011CRE	E726	K. pneun	NTU	1	I	R	R	0.25
2011CRE	E727	K. pneun	NTU	2	R	R	R	1
2011CRE	E728	K. pneun	NTU	128	R	R	R	16
2011CRE	E729	K. pneun	NTU	2	R	R	R	1
2011CRE	E730	K. pneun	NTU	1	I	R	R	2
2011CRE	E731	K. pneun	NTU	2	R	R	R	2
2011CRE	E732	K. pneun	NTU	1	I	R	R	1
2011CRE	E733	C. freunc	NTU	1	I	R	R	8
2011CRE	E735	K. pneun	NTU	8	R	R	R	4
2011CRE	E736	K. pneun	NTU	1	I	R	R	0.25
2011CRE	E737	S. marce	NTU	16	R	R	R	4
2011CRE	E740	E. cloaca	NTU	2	R	R	R	0.25
2011CRE	E742	E. cloaca	NTU	2	R	R	R	0.5
2011CRE	E743	E. cloaca	NTU	1	I	R	R	0.25
2011CRE	E744	K. pneun	NTU	2	R	R	R	1
2011CRE	E745	K. pneun	NTU	1	I	R	R	1
2011CRE	E746	K. pneun	NTU	128	R	R	R	16
2011CRE	E749	K. pneun	NTU	1	I	R	R	0.25
2011CRE	E750	E. coli	NTU	4	R	R	R	0.5
2011CRE	E752	E. coli	NTU	1	I	R	R	0.25
2011CRE	E753	K. pneun	NTU	1	I	R	R	0.5
2011CRE	E754	K. pneun	NTU	64	R	R	R	8
2011CRE	E755	K. pneun	NTU	4	R	R	R	1
2011CRE	E756	E. coli	NTU	4	R	R	R	0.25
2011CRE	E757	K. pneun	NTU	1	I	R	R	0.125
2011CRE	E759	E. aeroge	NTU	1	I	R	R	0.25
2011CRE	E760	K. pneun	NTU	2	R	R	R	0.5
2011CRE	E763	K. pneun	NTU	128	R	R	R	16
2011CRE	E764	E. cloaca	NTU	1	I	R	R	4

2011CRE	E767	K. pneun	NTU	2	R	R	R	1
2011CRE	E768	E. coli	NTU	4	R	R	R	1
2011CRE	E769	E. coli	NTU	1	I	R	R	0.25
2011CRE	E770	K. pneun	NTU	2	R	R	R	1
2011CRE	E771	K. pneun	NTU	1	I	R	R	1
2011CRE	E773	E. cloaca	NTU	8	R	R	R	0.5
2011CRE	E774	K. pneun	NTU	1	I	R	R	0.5
2011CRE	E775	E. cloaca	NTU	1	I	R	R	0.25
2011CRE	E776	K. pneun	NTU	2	R	R	R	0.5
2011CRE	E777	K. pneun	NTU	2	R	R	R	0.125
2011CRE	E778	K. pneun	NTU	1	I	R	R	2
2011CRE	E779	K. pneun	NTU	1	I	R	R	1
2011CRE	E780	E. cloaca	NTU	2	R	R	R	0.25
2011CRE	E783	K. pneun	NTU	1	I	R	R	0.5
2011CRE	E784	K. pneun	NTU	2	R	R	R	1
2011CRE	E785	K. pneun	NTU	2	R	R	R	0.5
2011CRE	E786	K. pneun	NTU	2	R	R	R	0.125
2011CRE	E787	K. pneun	NTU	1	I	R	R	0.25
2011CRE	E788	K. pneun	NTU	32	R	R	R	8
2011CRE	E789	K. pneun	NTU	4	R	R	R	0.5
2011CRE	E791	E. cloaca	NTU	1	I	R	R	0.25
2011CRE	E793	E. aeroge	NTU	64	R	R	R	8
2011CRE	E798	E. coli	NTU	1	I	R	R	0.25
2011CRE	E799	E. aeroge	NTU	1	I	R	R	1
2011CRE	E800	K. pneun	NTU	2	R	R	R	0.5
2011CRE	E801	K. pneun	NTU	32	R	R	R	2
2011CRE	E802	E. coli	NTU	2	R	R	R	0.5
2011CRE	E803	K. pneun	NTU	2	R	R	R	1
2011CRE	E804	E. cloaca	NTU	2	R	R	R	1
2011CRE	E805	E. cloaca	NTU	2	R	R	R	1
2011CRE	E806	E. aeroge	NTU	1	I	R	R	2
2011CRE	E807	E. coli	NTU	2	R	R	R	1
2011CRE	E808	K. pneun	NTU	2	R	R	R	1
2011CRE	E809	K. pneun	NTU	8	R	R	R	8
2011CRE	E810	K. pneun	NTU	2	R	R	R	0.5
2011CRE	E811	E. cloaca	NTU	2	R	R	R	1
2011CRE	E813	E. coli	NTU	2	R	R	R	1
2011CRE	E814	K. pneun	NTU	1	I	R	R	0.5
2011CRE	E816	E. aeroge	NTU	2	R	R	R	2
2011CRE	E817	E. cloaca	NTU	2	R	R	R	0.25
2011CRE	E819	K. pneun	NTU	1	I	R	R	0.5
2011CRE	E820	E. aeroge	NTU	4	R	R	R	0.5
2011CRE	E822	K. pneun	NTU	128	R	R	R	16
2011CRE	E823	E. cloaca	NTU	4	R	R	R	1
2011CRE	E824	E. aeroge	NTU	1	I	R	R	1
2011CRE	E826	K. pneun	NTU	4	R	R	R	1

2011CRE	E827	E. coli	NTU	1	I	R	R	0.25
2011CRE	E828	K. pneum	NTU	2	R	R	R	1
2011CRE	E831	K. pneum	NTU	16	R	R	R	2
2011CRE	E832	K. pneum	NTU	2	R	R	R	0.5
2011CRE	E833	E. cloaca	NTU	16	R	R	R	1
2011CRE	E834	E. cloaca	NTU	4	R	R	R	16
2011CRE	E835	K. pneum	NTU	2	R	R	R	1
2011CRE	E836	E. cloaca	NTU	4	R	R	R	0.5
2011CRE	E839	K. pneum	NTU	2	R	R	R	1
2011CRE	E840	E. coli	NTU	4	R	R	R	1
2011CRE	E841	K. pneum	NTU	1	I	R	R	0.5
2011CRE	E844	E. aeroge	NTU	2	R	R	R	2
2011CRE	E845	E. cloaca	NTU	2	R	R	R	0.5
2011CRE	E846	E. cloaca	NTU	1	I	R	R	0.25
2011CRE	E848	E. coli	NTU	4	R	R	R	0.25
2011CRE	E849	E. coli	NTU	2	R	R	R	0.5
2011CRE	E850	E. cloaca	NTU	1	I	R	R	0.5
2011CRE	E851	K. pneum	NTU	2	R	R	R	0.5
2011CRE	E853	E. cloaca	NTU	0.5	S	I	S	2
2011CRE	E854	E. aeroge	NTU	32	R	R	R	8
2011CRE	E855	E. cloaca	NTU	1	I	R	R	0.25
2011CRE	E858	E. coli	NTU	4	R	R	R	1
2011CRE	E859	C. freund	NTU	2	R	R	R	0.25
2011CRE	E860	E. cloaca	NTU	1	I	R	R	0.25
2011CRE	E861	E. coli	NTU	2	R	R	R	1
2011CRE	E862	K. pneum	NTU	1	I	R	R	0.25
2011CRE	E863	K. pneum	NTU	1	I	R	R	0.125
2011CRE	E864	K. pneum	NTU	1	I	R	R	1
2011CRE	E865	E. cloaca	NTU	16	R	R	R	2
2011CRE	E866	E. cloaca	NTU	2	R	R	R	0.5
2011CRE	E867	K. pneum	NTU	1	I	R	R	0.5
2011CRE	E869	E. cloaca	NTU	2	R	R	R	2
2011CRE	E870	E. cloaca	NTU	1	I	R	R	0.5
2011CRE	E871	K. pneum	NTU	2	R	R	R	1
2011CRE	E873	E. coli	NTU	2	R	R	R	0.25
2011CRE	E874	P. stuarti	NTU	4	R	R	R	2
2011CRE	E875	K. pneum	NTU	1	I	R	R	0.5
2011CRE	E876	E. cloaca	NTU	1	I	R	R	0.25
2011CRE	E877	K. pneum	NTU	1	I	R	R	0.5
2011CRE	E878	E. cloaca	NTU	2	R	R	R	0.5
2011CRE	E880	K. pneum	NTU	8	R	R	R	1
2011CRE	E881	K. pneum	NTU	2	R	R	R	0.5
2011CRE	E882	K. pneum	NTU	2	R	R	R	0.5
2011CRE	E884	K. pneum	NTU	1	I	R	R	0.5
2011CRE	E885	K. plantic	NTU	2	R	R	R	1
2011CRE	E892	E. cloaca	NTU	1	I	R	R	0.5

2011CRE	E893	E. coli	NTU	2	R	R	R	0.25
2011CRE	E894	E. aeroge	NTU	1	I	R	R	0.5
2011CRE	E896	K. pneun	NTU	1	I	R	R	0.5
2011CRE	E897	K. pneun	NTU	2	R	R	R	0.125
2011CRE	E899	E. coli	NTU	2	R	R	R	1
2011CRE	E900	K. pneun	NTU	2	R	R	R	0.5
2011CRE	E901	K. pneun	NTU	64	R	R	R	16
2011CRE	E903	K. pneun	NTU	8	R	R	R	1
2011CRE	E905	K. pneun	NTU	1	I	R	R	0.5
2011CRE	E906	E. aeroge	NTU	1	I	R	R	1
2011CRE	E907	E. cloaca	NTU	2	R	R	R	1
2011CRE	E908	E. cloaca	NTU	1	I	R	R	0.5
2011CRE	E909	K. pneun	NTU	4	R	R	R	1
2011CRE	E910	E. aeroge	NTU	1	I	R	R	0.25
2011CRE	E911	E. cloaca	NTU	1	I	R	R	0.5
2011CRE	E912	K. pneun	NTU	1	I	R	R	1
2011CRE	E914	E. coli	NTU	32	R	R	R	4
2011CRE	E915	C. freund	NTU	1	I	R	R	0.5
2011CRE	E917	K. pneun	NTU	1	I	R	R	1
2011CRE	E922	E. cloaca	NTU	1	I	R	R	0.5
2011CRE	E924	E. cloaca	NTU	8	R	R	R	2
2011CRE	E927	E. coli	NTU	4	R	R	R	1
2011CRE	E928	E. coli	NTU	16	R	R	R	2
2011CRE	E929	K. pneun	NTU	1	I	R	R	0.5
2011CRE	E930	K. pneun	NTU	1	I	R	R	2
2011CRE	E931	K. pneun	NTU	2	R	R	R	1
2011CRE	E932	K. pneun	NTU	8	R	R	R	1
2011CRE	E933	K. pneun	NTU	1	I	R	R	1
2011CRE	E937	E. aeroge	NTU	2	R	R	R	0.25
2011CRE	E938	C. freund	NTU	2	R	R	R	0.5
2011CRE	E939	E. aeroge	NTU	16	R	R	R	2
2011CRE	E940	E. aeroge	NTU	1	I	R	R	2
2011CRE	E942	E. aeroge	NTU	1	I	R	R	0.5
2011CRE	E943	E. aeroge	NTU	1	I	R	R	0.25
2011CRE	E944	E. cloaca	NTU	8	R	R	R	4
2011CRE	E946	E. cloaca	NTU	64	R	R	R	64
2011CRE	E947	E. cloaca	NTU	4	R	R	R	1
2011CRE	E948	E. cloaca	NTU	1	I	R	R	0.25
2011CRE	E949	E. cloaca	NTU	16	R	R	R	2
2011CRE	E950	E. cloaca	NTU	1	I	R	R	0.5
2011CRE	E951	E. coli	NTU	2	R	R	R	1
2011CRE	E952	E. coli	NTU	1	I	R	R	0.125
2011CRE	E954	E. coli	NTU	2	R	R	R	0.5
2011CRE	E955	E. coli	NTU	32	R	R	R	16
2011CRE	E956	K. pneun	NTU	2	R	R	R	0.125
2011CRE	E957	K. pneun	NTU	2	R	R	R	0.5

2011CRE	E958	K. pneun	NTU	1	I	R	R	0.5
2011CRE	E962	K. pneun	NTU	1	I	R	R	1
2011CRE	E963	K. pneun	NTU	1	I	R	R	0.5
2011CRE	E965	E. cloaca	NTU	1	I	R	R	0.5
2011CRE	E966	K. pneun	NTU	2	R	R	R	0.5
2011CRE	E967	K. pneun	NTU	2	R	R	R	0.5
2011CRE	E968	K. pneun	NTU	1	I	R	R	0.25
2011CRE	E969	K. pneun	NTU	1	I	R	R	0.5
2011CRE	E970	E. cloaca	NTU	1	I	R	R	0.25
2011CRE	E971	E. cloaca	NTU	1	I	R	R	0.25
2011CRE	E972	E. cloaca	NTU	2	R	R	R	0.5
2011CRE	E973	E. cloaca	NTU	1	I	R	R	0.5
2011CRE	E974	K. pneun	NTU	0.5	S	I	S	2
2011CRE	E975	E. cloaca	NTU	1	I	R	R	0.25
2011CRE	E977	E. cloaca	NTU	2	R	R	R	0.5
2011CRE	E978	K. pneun	NTU	1	I	R	R	0.25
2011CRE	E980	E. cloaca	NTU	1	I	R	R	0.5
2011CRE	E982	K. pneun	NTU	2	R	R	R	0.25
2011CRE	E984	E. cloaca	NTU	1	I	R	R	0.5
2011CRE	E985	E. cloaca	NTU	8	R	R	R	0.5
2011CRE	E986	K. pneun	NTU	1	I	R	R	2
2011CRE	E987	E. cloaca	NTU	4	R	R	R	0.5
2011CRE	E988	E. cloaca	NTU	1	I	R	R	0.5
2011CRE	E989	E. coli	NTU	2	R	R	R	0.25
2011CRE	E991	E. aeroge	NTU	4	R	R	R	2
2011CRE	E995	E. aeroge	NTU	4	R	R	R	2
2011CRE	E997	E. cloaca	NTU	1	I	R	R	0.25
2011CRE	E998	E. cloaca	NTU	32	R	R	R	8
2011CRE	E999	E. cloaca	NTU	64	R	R	R	16
2011CRE	E1000	E. cloaca	NTU	1	I	R	R	1
2011CRE	F002	E. aeroge	NTU	32	R	R	R	4
2011CRE	F004	E. aeroge	NTU	128	R	R	R	16
2011CRE	F006	K. pneun	NTU	4	R	R	R	2
2011CRE	F007	K. pneun	NTU	2	R	R	R	0.5
2011CRE	F009	K. pneun	NTU	1	I	R	R	0.25
2011CRE	F011	K. plantic	NTU	16	R	R	R	64
2011CRE	F013	M. morg	NTU	2	R	R	R	4
2011CRE	F014	P. rettge	NTU	4	R	R	R	2
2011CRE	F015	K. pneun	NTU	2	R	R	R	1
2011CRE	F016	E. coli	NTU	1	I	R	R	0.5
2011CRE	F017	E. cloaca	NTU	1	I	R	R	0.5
2011CRE	F018	K. pneun	NTU	4	R	R	R	2
2011CRE	F019	E. aeroge	NTU	2	R	R	R	0.5
2011CRE	F020	E. cloaca	NTU	4	R	R	R	0.5
2011CRE	F021	E. cloaca	NTU	1	I	R	R	1
2011CRE	F022	E. cloaca	NTU	1	I	R	R	0.5

2011CRE	F023	E. cloaca	NTU	2	R	R	R	1
2011CRE	F025	E. cloaca	NTU	4	R	R	R	0.5
2011CRE	F026	E. cloaca	NTU	2	R	R	R	2
2011CRE	F027	E. cloaca	NTU	2	R	R	R	0.5
2011CRE	F028	E. cloaca	NTU	1	I	R	R	0.25
2011CRE	F029	C. koseri	NTU	2	R	R	R	1
2011CRE	F030	E. aeroge	NTU	1	I	R	R	0.5
2011CRE	F032	E. cloaca	NTU	4	R	R	R	1
2011CRE	F036	E. cloaca	NTU	2	R	R	R	1
2011CRE	F037	E. cloaca	NTU	8	R	R	R	8
2011CRE	F038	E. cloaca	NTU	1	I	R	R	0.5
2011CRE	F039	E. cloaca	NTU	1	I	R	R	0.5
2011CRE	F041	E. cloaca	NTU	4	R	R	R	1
2011CRE	F042	E. cloaca	NTU	2	R	R	R	0.5
2011CRE	F044	E. cloaca	NTU	1	I	R	R	0.5
2011CRE	F046	E. coli	NTU	1	I	R	R	0.5
2011CRE	F178	K. pneum	YTM	1	I	R	R	0.25
2011CRE	F182	K. pneum	YTM	2	R	R	R	1
2011CRE	F183	K. pneum	YTM	2	R	R	R	1
2011CRE	F184	E. aeroge	YTM	2	R	R	R	1
2011CRE	F190	K. pneum	YTM	128	R	R	R	16
2011CRE	F192	K. pneum	YTM	2	R	R	R	2
2011CRE	F193	K. pneum	YTM	4	R	R	R	2
2011CRE	F194	E. coli	YTM	4	R	R	R	0.5
2011CRE	F196	K. pneum	YTM	64	R	R	R	8
2011CRE	F197	S. marce	YTM	128	R	R	R	8
2011CRE	F200	E. cloaca	YTM	16	R	R	R	8
2011CRE	F203	E. coli	YTM	2	R	R	R	0.5
2011CRE	F204	K. pneum	YTM	>128	R	R	R	64
2011CRE	F206	M. morg	YTM	0.5	S	I	S	4
2011CRE	F209	K. pneum	YTM	2	R	R	R	1
2011CRE	F211	K. pneum	YTM	1	I	R	R	1
2011CRE	F212	E. cloaca	YTM	4	R	R	R	0.5
2011CRE	F216	K. pneum	YTM	2	R	R	R	2
2011CRE	F222	E. coli	YTM	1	I	R	R	0.25
2011CRE	F224	E. aeroge	YTM	128	R	R	R	0.5
2011CRE	F225	E. aeroge	YTM	1	I	R	R	8
2011CRE	F228	C. freund	YTM	1	I	R	R	1
2011CRE	F229	E. cloaca	YTM	4	R	R	R	8
2011CRE	F230	E. cloaca	YTM	1	I	R	R	4
2011CRE	F231	K. pneum	YTM	1	I	R	R	0.25
2011CRE	F232	E. cloaca	YTM	2	R	R	R	2
2011CRE	F234	K. pneum	YTM	1	I	R	R	2
2011CRE	F236	K. pneum	YTM	>128	R	R	R	64
2011CRE	F237	K. pneum	YTM	>128	R	R	R	64
2011CRE	F238	K. pneum	YTM	>128	R	R	R	32

2011CRE	F239	K. pneun	YTM	1	I	R	R	1
2011CRE	F240	E. cloaca	YTM	2	R	R	R	1
2011CRE	F243	E. cloaca	YTM	1	I	R	R	0.25
2011CRE	F248	E. cloaca	YTM	2	R	R	R	0.25
2011CRE	F250	K. pneun	YTM	>128	R	R	R	64
2011CRE	F251	K. pneun	YTM	>128	R	R	R	64
2011CRE	F254	K. pneun	YTM	2	R	R	R	2
2011CRE	F256	K. pneun	YTM	4	R	R	R	2
2011CRE	F257	K. pneun	YTM	4	R	R	R	1
2011CRE	F259	K. pneun	YTM	2	R	R	R	1
2011CRE	F260	E. coli	YTM	4	R	R	R	0.5
2011CRE	F261	P. stuarti	YTM	4	R	R	R	4
2011CRE	F262	K. pneun	YTM	2	R	R	R	2
2011CRE	F263	K. pneun	YTM	4	R	R	R	2
2011CRE	F266	E. cloaca	YTM	4	R	R	R	0.5
2011CRE	F268	E. cloaca	YTM	4	R	R	R	1
2011CRE	F276	K. pneun	NTU	2	R	R	R	0.25
2011CRE	F277	K. pneun	NTU	1	I	R	R	0.5
2011CRE	F278	K. pneun	NTU	2	R	R	R	1
2011CRE	F279	K. pneun	NTU	2	R	R	R	1
2011CRE	F280	K. pneun	NTU	32	R	R	R	2
2011CRE	F281	K. pneun	NTU	1	I	R	R	0.5
2011CRE	F282	K. pneun	NTU	32	R	R	R	4
2011CRE	F283	K. pneun	NTU	16	R	R	R	2
2011CRE	F284	K. pneun	NTU	1	I	R	R	0.5
2011CRE	F285	K. pneun	NTY	2	R	R	R	1
2011CRE	F286	K. pneun	NTY	8	R	R	R	4
2011CRE	F287	E. cloaca	NTY	4	R	R	R	1
2011CRE	F288	K. pneun	NTY	2	R	R	R	1
2011CRE	F289	E. coli	NTY	8	R	R	R	1
2011CRE	F290	E. cloaca	NTY	4	R	R	R	1
2011CRE	F291	K. pneun	NTY	2	R	R	R	1
2011CRE	F292	E. aeroge	NTY	1	I	R	R	0.5
2011CRE	F293	K. pneun	NTY	1	I	R	R	1
2011CRE	F294	K. pneun	NTY	2	R	R	R	1
2011CRE	F295	K. pneun	NTY	2	R	R	R	2
2011CRE	F296	E. coli	NTY	2	R	R	R	1
2011CRE	F298	K. pneun	NTY	2	R	R	R	1
2011CRE	F299	E. coli	NTY	4	R	R	R	0.5
2011CRE	F300	E. coli	NTY	2	R	R	R	0.5
2011CRE	F301	K. pneun	NTY	2	R	R	R	2
2011CRE	F302	K. pneun	NTY	>128	R	R	R	32
2011CRE	F303	E. coli	NTY	4	R	R	R	1
2011CRE	F304	K. pneun	NTY	2	R	R	R	2
2011CRE	F305	E. coli	NTY	>128	R	R	R	16
2011CRE	F306	K. pneun	NTY	2	R	R	R	1

2011CRE	F307	E. aeroge	NTY	2	R	R	R	0.5
2011CRE	F308	K. pneun	NTY	2	R	R	R	1
2011CRE	F309	K. pneun	NTY	2	R	R	R	2
2011CRE	F310	K. pneun	NTY	1	I	R	R	1
2011CRE	F311	E. coli	NTY	1	I	R	R	0.5
2011CRE	F313	K. pneun	NTY	2	R	R	R	1
2011CRE	F314	K. pneun	NTY	64	R	R	R	32
2011CRE	F315	K. pneun	NTY	4	R	R	R	2
2011CRE	F316	K. pneun	NTY	4	R	R	R	2
2011CRE	F317	K. oxyto	NTY	2	R	R	R	8
2011CRE	F318	K. pneun	NTY	2	R	R	R	0.5
2011CRE	F319	E. coli	NTY	2	R	R	R	0.5
2011CRE	F320	K. pneun	NTY	2	R	R	R	1
2011CRE	F321	S. marce	NTY	8	R	R	R	2
2011CRE	F322	K. pneun	NTY	2	R	R	R	2
2011CRE	F323	K. pneun	NTY	4	R	R	R	2
2011CRE	F324	E. coli	NTY	2	R	R	R	0.5
2011CRE	F326	K. pneun	NTY	2	R	R	R	0.5
2011CRE	F328	E. cloaca	NTY	1	I	R	R	0.25
2011CRE	F330	K. pneun	NTY	2	R	R	R	0.5
2011CRE	F333	K. pneun	NTY	2	R	R	R	2
2011CRE	F334	E. coli	NTY	8	R	R	R	1
2011CRE	F335	K. pneun	NTY	2	R	R	R	1
2011CRE	F336	K. pneun	NTY	2	R	R	R	1
2011CRE	F337	S. marce	NTY	8	R	R	R	8
2011CRE	F338	K. pneun	NTY	1	I	R	R	1
2011CRE	F339	K. pneun	NTY	2	R	R	R	2
2011CRE	F340	K. pneun	NTY	2	R	R	R	0.5
2011CRE	F344	K. pneun	NTY	2	R	R	R	1
2011CRE	F347	K. pneun	NTY	2	R	R	R	2
2011CRE	F349	K. pneun	NTY	2	R	R	R	1
2011CRE	F350	K. pneun	NTY	1	I	R	R	0.5
2011CRE	F351	K. pneun	NTY	128	R	R	R	32
2011CRE	F352	E. coli	NTY	1	I	R	R	0.25
2011CRE	F353	E. aeroge	NTY	2	R	R	R	0.25
2011CRE	F354	K. pneun	NTY	2	R	R	R	1
2011CRE	F355	K. pneun	NTY	2	R	R	R	1
2011CRE	F356	E. cloaca	NTY	2	R	R	R	1
2011CRE	F358	K. pneun	NTY	2	R	R	R	0.5
2011CRE	F359	S. marce	NTY	8	R	R	R	2
2011CRE	F360	E. coli	NTY	2	R	R	R	1
2011CRE	F362	K. pneun	NTY	4	R	R	R	2
2011CRE	F363	K. pneun	NTY	2	R	R	R	1
2011CRE	F364	K. pneun	NTY	16	R	R	R	2
2011CRE	F365	E. cloaca	NTY	4	R	R	R	2
2011CRE	F366	K. pneun	NTY	1	I	R	R	1

2011CRE	F369	K. pneun	NTY	2	R	R	R	0.5
2011CRE	F273	E. aeroge	YTM	1	I	R	R	0.5
2011CRE	F275	K. pneun	YTM	2	R	R	R	0.5
2011CRE	F385	K. pneun	YTM	2	R	R	R	1
2011CRE	F386	C. divers	YTM	2	R	R	R	2
2011CRE	F388	E. cloaca	YTM	1	I	R	R	0.25
2011CRE	F389	E. coli	YTM	8	R	R	R	4
2011CRE	F392	K. pneun	YTM	2	R	R	R	1
2011CRE	F397	K. pneun	YTM	2	R	R	R	0.25
2011CRE	F399	K. pneun	YTM	4	R	R	R	2
2011CRE	F400	E. cloaca	YTM	4	R	R	R	0.5
2011CRE	F403	K. pneun	YTM	2	R	R	R	1
2011CRE	F404	K. pneun	YTM	>128	R	R	R	32
2011CRE	F406	C. divers	YTM	2	R	R	R	0.5
2011CRE	F407	E. cloaca	YTM	1	I	R	R	0.5
2011CRE	F410	E. cloaca	YTM	2	R	R	R	1
2011CRE	F415	E. cloaca	YTM	1	I	R	R	0.25
2011CRE	F421	K. pneun	YTM	8	R	R	R	2
2011CRE	F423	E. cloaca	YTM	2	R	R	R	1
2011CRE	F426	E. coli	YTM	64	R	R	R	8
2011CRE	F427	K. pneun	YTM	2	R	R	R	1
2011CRE	F429	K. pneun	YTM	8	R	R	R	4
2011CRE	F431	E. cloaca	YTM	2	R	R	R	0.5
2011CRE	F432	E. cloaca	YTM	2	R	R	R	2
2011CRE	F434	K. pneun	YTM	4	R	R	R	4
2011CRE	F435	K. pneun	YTM	4	R	R	R	4
2011CRE	F436	K. pneun	YTM	1	I	R	R	0.25
2011CRE	F441	S. marce	YTM	1	I	R	R	4
2011CRE	F444	K. pneun	YTM	2	R	R	R	1
2011CRE	F447	E. cloaca	YTM	0.5	S	I	S	2
2011CRE	F448	E. coli	YTM	1	I	R	R	0.25
2011CRE	F451	K. pneun	YTM	>128	R	R	R	16
2011CRE	F453	E. coli	YTM	1	I	R	R	2
2011CRE	F457	K. pneun	YTM	2	R	R	R	0.5
2011CRE	F458	K. pneun	YTM	2	R	R	R	4
2011CRE	F462	C. divers	YTM	2	R	R	R	0.25
2011CRE	F464	E. aeroge	YTM	1	I	R	R	1
2011CRE	F466	K. pneun	YTM	16	R	R	R	2
2011CRE	F467	E. coli	YTM	8	R	R	R	1
2011CRE	F468	K. pneun	YTM	1	I	R	R	0.5
2011CRE	F470	K. pneun	YTM	1	I	R	R	0.25
2011CRE	F475	E. coli	YTM	2	R	R	R	0.5
2011CRE	F477	E. cloaca	YTM	4	R	R	R	1
2011CRE	F478	E. cloaca	YTM	1	I	R	R	2
2011CRE	F482	P. stuarti	YTM	16	R	R	R	4
2011CRE	F371	K. pneun	NTY	2	R	R	R	1

2011CRE	F372	E. cloaca	NTY	1	I	R	R	0.5
2011CRE	F373	K. pneun	NTY	2	R	R	R	1
2011CRE	F374	K. pneun	NTY	2	R	R	R	1
2011CRE	F376	K. pneun	NTY	2	R	R	R	2
2011CRE	F377	K. pneun	NTY	2	R	R	R	1
2011CRE	F378	K. pneun	NTY	2	R	R	R	1
2011CRE	F379	K. pneun	NTY	2	R	R	R	1
2011CRE	F381	K. pneun	NTY	1	I	R	R	1
2011CRE	F382	E. coli	NTY	4	R	R	R	1
2011CRE	F383	K. pneun	NTY	4	R	R	R	1
2011CRE	F384	K. pneun	NTY	64	R	R	R	4
2012CRE	F665	E. coli	YTM	8	R	R	R	8
2012CRE	F666	E. coli	YTM	2	R	R	R	0.5
2012CRE	F667	E. aeroge	YTM	1	I	R	R	2
2012CRE	F671	K. pneun	YTM	2	R	R	R	2
2012CRE	F672	K. pneun	YTM	4	R	R	R	0.25
2012CRE	F674	K. pneun	YTM	128	R	R	R	16
2012CRE	F679	E. cloaca	YTM	4	R	R	R	4
2012CRE	F680	E. cloaca	YTM	1	I	R	R	0.5
2012CRE	F681	E. cloaca	YTM	2	R	R	R	0.5
2012CRE	F684	E. cloaca	YTM	2	R	R	R	2
2012CRE	F685	E. cloaca	YTM	1	I	R	R	1
2012CRE	F687	E. coli	YTM	4	R	R	R	1
2012CRE	F688	E. coli	YTM	4	R	R	R	4
2012CRE	F690	E. cloaca	YTM	1	I	R	R	0.5
2012CRE	F695	K. pneun	YTM	1	I	R	R	1
2012CRE	F696	K. pneun	YTM	1	I	R	R	2
2012CRE	F697	K. pneun	YTM	2	R	R	R	0.5
2012CRE	F698	K. pneun	YTM	2	R	R	R	1
2012CRE	F699	K. pneun	YTM	2	R	R	R	0.5
2012CRE	F700	K. pneun	YTM	2	R	R	R	0.5
2012CRE	F701	K. pneun	YTM	2	R	R	R	0.5
2012CRE	F702	K. pneun	YTM	8	R	R	R	2
2012CRE	F703	K. pneun	YTM	2	R	R	R	1
2012CRE	F704	K. pneun	YTM	2	R	R	R	1
2012CRE	F706	P. stuarti	YTM	16	R	R	R	8
2012CRE	F708	E. coli	YTM	1	I	R	R	0.25
2012CRE	F711	E. cloaca	YTM	2	R	R	R	1
2012CRE	F712	K. pneun	YTM	16	R	R	R	4
2012CRE	F714	E. aeroge	YTM	1	I	R	R	0.5
2012CRE	F717	K. pneun	YTM	2	R	R	R	1
2012CRE	F721	K. pneun	YTM	2	R	R	R	0.5
2012CRE	F722	K. pneun	YTM	1	I	R	R	0.5
2012CRE	F725	E. coli	YTM	4	R	R	R	0.125
2012CRE	F726	E. cloaca	YTM	1	I	R	R	0.25
2012CRE	F728	K. pneun	YTM	1	I	R	R	1

2012CRE	F730	K. pneun	YTM	128	R	R	R	16
2012CRE	F732	E. coli	YTM	8	R	R	R	1
2012CRE	F733	K. pneun	YTM	1	I	R	R	0.5
2012CRE	F734	E. cloaca	YTM	2	R	R	R	2
2012CRE	F738	K. pneun	YTM	0.5	S	I	S	4
2012CRE	F741	K. pneun	YTM	2	R	R	R	1
2012CRE	F742	K. pneun	YTM	128	R	R	R	8
2012CRE	F744	K. pneun	YTM	64	R	R	R	4
2012CRE	F745	E. cloaca	YTM	0.5	S	I	S	2
2012CRE	F746	K. pneun	YTM	2	R	R	R	1
2012CRE	F747	K. pneun	YTM	8	R	R	R	4
2012CRE	F748	E. cloaca	YTM	2	R	R	R	0.5
2012CRE	F749	P. rettge	YTM	8	R	R	R	8
2012CRE	F750	K. pneun	YTM	2	R	R	R	1
2012CRE	F752	K. pneun	YTM	32	R	R	R	16
2012CRE	F756	K. pneun	YTM	4	R	R	R	1
2012CRE	F757	K. pneun	YTM	8	R	R	R	2
2012CRE	F760	K. pneun	YTM	16	R	R	R	2
2012CRE	F761	K. pneun	YTM	8	R	R	R	1
2012CRE	F506	E. aeroge	NTU	1	I	R	R	1
2012CRE	F509	K. pneun	NTU	2	R	R	R	2
2012CRE	F510	K. pneun	NTU	1	I	R	R	0.5
2012CRE	F511	E. coli	NTU	1	I	R	R	0.5
2012CRE	F512	E. aeroge	NTU	32	R	R	R	8
2012CRE	F513	E. aeroge	NTU	16	R	R	R	4
2012CRE	F517	E. cloaca	NTU	2	R	R	S	1
2012CRE	F519	E. cloaca	NTU	1	I	R	R	0.5
2012CRE	F520	E. cloaca	NTU	2	R	R	R	0.5
2012CRE	F521	K. pneun	NTU	64	R	R	R	16
2012CRE	F522	K. pneun	NTU	1	I	R	R	1
2012CRE	F523	K. pneun	NTU	2	R	R	R	0.5
2012CRE	F524	K. pneun	NTU	2	R	R	R	0.5
2012CRE	F525	K. pneun	NTU	2	R	R	R	0.5
2012CRE	F528	K. pneun	NTU	2	R	R	R	2
2012CRE	F529	K. pneun	NTU	1	I	R	R	0.5
2012CRE	F531	K. pneun	NTU	>128	R	R	R	32
2012CRE	F592	E. aeroge	NTU	1	I	R	R	0.5
2012CRE	F593	E. cloaca	NTU	4	R	R	R	0.5
2012CRE	F594	E. cloaca	NTU	1	I	R	R	0.5
2012CRE	F595	E. cloaca	NTU	1	I	R	R	0.5
2012CRE	F596	E. cloaca	NTU	1	I	R	R	0.5
2012CRE	F597	E. cloaca	NTU	4	R	R	R	1
2012CRE	F598	E. cloaca	NTU	1	I	R	R	0.25
2012CRE	F599	E. cloaca	NTU	1	I	R	R	1
2012CRE	F600	E. cloaca	NTU	2	R	R	R	0.25
2012CRE	F602	E. cloaca	NTU	1	I	R	R	0.5

2012CRE	F603	E. cloaca	NTU	1	I	R	R	1
2012CRE	F604	E. coli	NTU	2	R	R	R	0.25
2012CRE	F605	E. coli	NTU	1	I	R	R	0.5
2012CRE	F606	K. pneum	NTU	1	I	R	R	0.5
2012CRE	F607	K. pneum	NTU	1	I	R	R	0.5
2012CRE	F608	K. pneum	NTU	2	R	R	R	1
2012CRE	F609	K. pneum	NTU	2	R	R	R	1
2012CRE	F610	K. pneum	NTU	2	R	R	R	1
2012CRE	F611	K. pneum	NTU	1	I	R	R	0.5
2012CRE	F613	E. aeroge	NTU	2	R	R	R	1
2012CRE	F614	E. aeroge	NTU	8	R	R	R	4
2012CRE	F615	E. aeroge	NTU	1	I	R	R	0.25
2012CRE	F617	E. aeroge	NTU	2	R	R	R	0.5
2012CRE	F618	E. aeroge	NTU	1	I	R	R	0.5
2012CRE	F619	E. cloaca	NTU	8	R	R	R	1
2012CRE	F620	E. cloaca	NTU	2	R	R	R	0.5
2012CRE	F621	E. cloaca	NTU	1	I	R	R	0.5
2012CRE	F622	E. cloaca	NTU	1	I	R	R	0.5
2012CRE	F623	E. cloaca	NTU	2	R	R	R	0.5
2012CRE	F624	E. coli	NTU	2	R	R	R	1
2012CRE	F626	E. coli	NTU	2	R	R	R	0.5
2012CRE	F627	K. pneum	NTU	4	R	R	R	1
2012CRE	F628	K. pneum	NTU	4	R	R	R	0.5
2012CRE	F629	K. pneum	NTU	1	I	R	R	1
2012CRE	F631	K. pneum	NTU	2	R	R	R	0.5
2012CRE	F632	K. pneum	NTU	64	R	R	R	8
2012CRE	F633	K. pneum	NTU	4	R	R	R	2
2012CRE	F634	K. pneum	NTU	2	R	R	R	1
2012CRE	F635	K. pneum	NTU	1	I	R	R	1
2012CRE	F636	K. pneum	NTU	2	R	R	R	1
2012CRE	F637	K. pneum	NTU	1	I	R	R	0.5
2012CRE	F639	K. pneum	NTU	128	R	R	R	4
2012CRE	F642	C. freund	NTU	1	I	R	R	0.5
2012CRE	F644	E. aeroge	NTU	64	R	R	R	8
2012CRE	F645	E. cloaca	NTU	2	R	R	R	0.5
2012CRE	F646	E. cloaca	NTU	2	R	R	R	0.5
2012CRE	F647	E. cloaca	NTU	4	R	R	R	0.25
2012CRE	F649	E. coli	NTU	1	I	R	R	0.25
2012CRE	F650	E. coli	NTU	1	I	R	R	0.25
2012CRE	F651	E. coli	NTU	>128	R	R	R	64
2012CRE	F652	K. pneum	NTU	2	R	R	R	0.5
2012CRE	F653	K. pneum	NTU	2	R	R	R	1
2012CRE	F654	K. pneum	NTU	8	R	R	R	1
2012CRE	F655	K. pneum	NTU	8	R	R	R	1
2012CRE	F656	K. pneum	NTU	1	I	R	R	0.25
2012CRE	F657	K. pneum	NTU	2	R	R	R	1

2012CRE	F659	K. pneum	NTU	2	R	R	R	0.125
2012CRE	F660	K. pneum	NTU	>128	R	R	R	128
2012CRE	F661	M. morg	NTU	2	R	R	R	4
2012CRE	F663	C. freunc	NTU	1	I	R	R	2
2012CRE	F664	C. freunc	NTU	32	R	R	R	2
2012CRE	F763	C. freunc	NTU	2	R	R	R	0.5
2012CRE	F764	E. aeroge	NTU	1	I	R	R	0.5
2012CRE	F765	E. aeroge	NTU	1	I	R	R	0.5
2012CRE	F766	E. aeroge	NTU	1	I	R	R	1
2012CRE	F767	E. cloaca	NTU	4	R	R	R	0.5
2012CRE	F768	E. cloaca	NTU	1	I	R	R	0.5
2012CRE	F769	E. cloaca	NTU	4	R	R	R	2
2012CRE	F770	E. cloaca	NTU	2	R	R	R	0.5
2012CRE	F771	E. cloaca	NTU	1	I	R	R	0.5
2012CRE	F772	E. cloaca	NTU	16	R	R	R	2
2012CRE	F773	E. cloaca	NTU	2	R	R	R	0.25
2012CRE	F774	E. coli	NTU	8	R	R	R	0.25
2012CRE	F775	E. coli	NTU	1	I	R	R	0.25
2012CRE	F776	K. pneum	NTU	1	I	R	R	0.5
2012CRE	F777	K. pneum	NTU	1	I	R	R	1
2012CRE	F778	K. pneum	NTU	2	R	R	R	0.5
2012CRE	F779	K. pneum	NTU	8	R	R	R	0.5
2012CRE	F780	K. pneum	NTU	128	R	R	R	4
2012CRE	F782	K. pneum	NTU	2	R	R	R	0.5
2012CRE	F783	K. pneum	NTU	2	R	R	R	2
2012CRE	F784	K. pneum	NTU	1	I	R	R	0.25
2012CRE	F785	K. pneum	NTU	1	I	R	R	0.25
2012CRE	F786	K. pneum	NTU	2	R	R	R	0.5
2012CRE	F787	K. pneum	NTU	4	R	R	R	4
2012CRE	F788	K. pneum	NTU	1	I	R	R	0.125
2012CRE	F789	K. pneum	NTU	2	R	R	R	0.5
2012CRE	F792	E. aeroge	NTU	1	I	R	R	0.25
2012CRE	F794	E. aeroge	NTU	1	I	R	R	0.5
2012CRE	F795	E. cloaca	NTU	1	I	R	R	1
2012CRE	F797	E. cloaca	NTU	4	R	R	R	0.5
2012CRE	F798	E. cloaca	NTU	8	R	R	R	1
2012CRE	F799	E. coli	NTU	1	I	R	R	0.125
2012CRE	F800	E. coli	NTU	2	R	R	R	0.25
2012CRE	F801	K. pneum	NTU	4	R	R	R	0.5
2012CRE	F802	K. pneum	NTU	1	I	R	R	1
2012CRE	F803	K. pneum	NTU	1	I	R	R	1
2012CRE	F804	K. pneum	NTU	1	I	R	R	0.125
2012CRE	F805	K. pneum	NTU	1	I	R	R	0.25
2012CRE	F806	K. pneum	NTU	2	R	R	R	0.25
2012CRE	F807	K. pneum	NTU	128	R	R	R	16
2012CRE	G236	C. freunc	NTU	1	I	R	R	0.25

2012CRE	G237	E. aeroge	NTU	2	R	R	R	0.5
2012CRE	G238	E. cloaca	NTU	1	I	R	R	0.5
2012CRE	G239	E. cloaca	NTU	4	R	R	R	2
2012CRE	G240	E. cloaca	NTU	2	R	R	R	0.25
2012CRE	F795	E. cloaca	NTU	1	I	R	R	1
2012CRE	F806	K. pneum	NTU	2	R	R	R	0.25
2012CRE	F807	K. pneum	NTU	128	R	R	R	16
2012CRE	G273	E. cloaca	NTU	2	R	R	R	2
2012CRE	G297	E. aeroge	NTU	16	R	R	R	4
2012CRE	G321	K. pneum	NTU	2	R	R	R	0.25
2012CRE	G322	K. pneum	NTU	4	R	R	R	0.125
2012CRE	G018	K. pneum	NTU	2	R	R	R	0.5
2012CRE	G051	K. pneum	NTU	2	R	R	R	0.25
2012CRE		E. cloaca	NTU	1	I	R	R	0.5
2012CRE		S. marce	NTU	4	R	R	R	1
2012CRE		E. coli	NTU	4	R	R	R	0.125
2012CRE		E. aeroge	NTU	1	I	R	R	0.5
2012CRE		E. coli	NTU	8	R	R	R	0.5
2012CRE		E. coli	NTU	32	R	R	R	2
2012CRE	G572	K. planti	NTU	4	R	R	R	1
2012CRE	G573	E. coli	NTU	4	R	R	R	2
2012CRE		K. pneum	NTU	128	R	R	R	16
2012CRE	G324	E. cloaca	NTU	4	R	R	R	0.5
2012CRE	G329	E. aeroge	NTU	1	I	R	R	0.5
2012CRE	G330	M. morg	NTU	0.5	S	I	S	2
2012CRE	G486	K. pneum	NTU	4	R	R	R	1
2012CRE	G560	E. cloaca	NTU	8	R	R	R	0.25
2012CRE	G566	E. cloaca	NTU	2	R	R	R	0.25
2012CRE	G711	K. pneum	NTU	2	R	R	R	0.5
2012CRE	G712	K. pneum	NTU	4	R	R	R	1
2012CRE	G713	E. coli	NTU	32	R	R	R	8
2012CRE	G714	E. coli	NTU	2	R	R	R	1
2012CRE	G715	K. pneum	NTU	4	R	R	R	1
2012CRE	G010	K. pneum	NTY	2	R	R	R	0.5
2012CRE	G011	K. pneum	NTY	2	R	R	R	0.25
2012CRE	G012	K. pneum	NTY	4	R	R	R	2
2012CRE	G013	E. coli	NTY	2	R	R	R	0.5
2012CRE	G014	E. coli	NTY	2	R	R	R	0.125
2012CRE	G015	K. pneum	NTY	2	R	R	R	0.5
2012CRE	G016	K. pneum	NTY	2	R	R	R	0.5
2012CRE	G017	K. pneum	NTY	1	I	R	R	0.25
2012CRE	G018	K. pneum	NTY	2	R	R	R	0.5
2012CRE	G019	E. coli	NTY	2	R	R	R	0.5
2012CRE	G020	K. pneum	NTY	4	R	R	R	0.5
2012CRE	G021	K. pneum	NTY	>128	R	R	R	32
2012CRE	G022	E. coli	NTY	2	R	R	R	0.5

2012CRE	G023	K. pneun	NTY	8	R	R	R	2
2012CRE	G024	K. pneun	NTY	4	R	R	R	0.5
2012CRE	G025	K. pneun	NTY	4	R	R	R	0.5
2012CRE	G026	E. cloaca	NTY	1	I	R	R	0.25
2012CRE	G027	E. cloaca	NTY	4	R	R	R	2
2012CRE	G028	E. coli	NTY	128	R	R	R	16
2012CRE	G029	E. cloaca	NTY	64	R	R	R	8
2012CRE	G030	E. coli	NTY	2	R	R	R	1
2012CRE	G031	K. pneun	NTY	2	R	R	R	1
2012CRE	G032	K. pneun	NTY	2	R	R	R	1
2012CRE	G033	K. pneun	NTY	2	R	R	R	0.5
2012CRE	G034	K. pneun	NTY	1	I	R	R	1
2012CRE	G035	K. pneun	NTY	2	R	R	R	1
2012CRE	G037	K. pneun	NTY	4	R	R	R	0.5
2012CRE	G038	K. pneun	NTY	2	R	R	R	1
2012CRE	G039	K. pneun	NTY	2	R	R	R	0.5
2012CRE	G041	K. pneun	NTY	1	I	R	R	0.5
2012CRE	G042	K. pneun	NTY	1	I	R	R	1
2012CRE	G043	K. pneun	NTY	1	I	R	R	0.5
2012CRE	G044	K. pneun	NTY	1	I	R	R	1
2012CRE	G046	K. pneun	NTY	4	R	R	R	1
2012CRE	G047	K. pneun	NTY	1	I	R	R	0.5
2012CRE	G048	K. pneun	NTY	2	R	R	R	1
2012CRE	G049	K. pneun	NTY	2	R	R	R	1
2012CRE	G050	K. pneun	NTY	2	R	R	R	1
2012CRE	G051	K. pneun	NTY	2	R	R	R	0.25
2012CRE	G053	K. pneun	NTY	2	R	R	R	0.25
2012CRE	G054	K. pneun	NTY	2	R	R	R	0.25
2012CRE	G055	E. aeroge	NTY	1	I	R	R	0.5
2012CRE	G056	K. pneun	NTY	2	R	R	R	0.25
2012CRE	G057	E. coli	NTY	1	I	R	R	0.25
2012CRE	G058	K. pneun	NTY	4	R	R	R	0.25
2012CRE	G059	K. pneun	NTY	2	R	R	R	1
2012CRE	G060	K. pneun	NTY	2	R	R	R	4
2012CRE	G061	K. pneun	NTY	2	R	R	R	0.25
2012CRE	G062	K. pneun	NTY	2	R	R	R	0.5
2012CRE	G064	K. pneun	NTY	2	R	R	R	2
2012CRE	G065	K. pneun	NTY	2	R	R	R	0.5
2012CRE	G066	K. pneun	NTY	1	I	R	R	0.25
2012CRE	G068	K. pneun	NTY	>128	R	R	R	16
2012CRE	G069	K. pneun	NTY	2	R	R	R	2
2012CRE	G070	K. pneun	NTY	2	R	R	R	0.5
2012CRE	G072	K. pneun	NTY	2	R	R	R	1
2012CRE	G073	K. pneun	NTY	4	R	R	R	0.5
2012CRE	G075	K. pneun	NTY	1	I	R	R	1
2012CRE	G077	E. coli	NTY	2	R	R	R	0.5

2012CRE	G078	K. pneun	NTY	2	R	R	R	0.5
2012CRE	G079	K. pneun	NTY	4	R	R	R	0.5
2012CRE	G080	E. coli	NTY	2	R	R	R	0.25
2012CRE	G081	K. pneun	NTY	4	R	R	R	0.5
2012CRE	G082	K. pneun	NTY	2	R	R	R	0.5
2012CRE	G083	K. pneun	NTY	2	R	R	R	1
2012CRE	G084	K. pneun	NTY	4	R	R	R	1
2012CRE	G085	K. pneun	NTY	2	R	R	R	0.5
2012CRE	G086	K. pneun	NTY	2	R	R	R	1
2012CRE	G087	K. pneun	NTY	2	R	R	R	0.5
2012CRE	G089	E. cloaca	NTY	2	R	R	R	1
2012CRE	G090	E. cloaca	NTY	2	R	R	R	2
2012CRE	G091	E. coli	NTY	2	R	R	R	0.25
2012CRE	G092	E. cloaca	NTY	2	R	R	R	1
2012CRE	G093	E. coli	NTY	2	R	R	R	0.5
2012CRE	G094	K. pneun	NTY	64	R	R	R	4
2012CRE	G095	E. cloaca	NTY	1	I	R	R	1
2012CRE	G096	K. pneun	NTY	2	R	R	R	0.5
2012CRE	G097	K. pneun	NTY	2	R	R	R	0.5
2012CRE	G098	E. cloaca	NTY	4	R	R	R	4
2012CRE	G099	E. coli	NTY	2	R	R	R	0.5
2012CRE	G100	E. cloaca	NTY	4	R	R	R	1
2012CRE	G101	K. pneun	NTY	4	R	R	R	0.125
2012CRE	G102	K. pneun	NTY	8	R	R	R	0.25
2012CRE	G103	E. coli	NTY	1	I	R	R	0.25
2012CRE	G104	E. coli	NTY	32	R	R	R	8
2012CRE	G105	K. pneun	NTY	2	R	R	R	2
2012CRE	G242	E. cloaca	NTU	2	R	R	R	0.5
2012CRE	G243	E. cloaca	NTU	4	R	R	R	0.5
2012CRE	G244	E. coli	NTU	4	R	R	R	0.125
2012CRE	G245	K. pneun	NTU	1	I	R	R	0.5
2012CRE	G246	K. pneun	NTU	2	R	R	R	0.25
2012CRE	G247	K. pneun	NTU	8	R	R	R	1
2012CRE	G248	K. pneun	NTU	2	R	R	R	0.25
2012CRE	G249	K. pneun	NTU	2	R	R	R	1
2012CRE	G251	K. pneun	NTU	1	I	R	R	0.5
2012CRE	G252	K. pneun	NTU	4	R	R	R	0.5
2012CRE	G253	K. pneun	NTU	2	R	R	R	1
2012CRE	G254	K. pneun	NTU	>128	R	R	R	64
2012CRE	G255	K. plantic	NTU	2	R	R	R	1
2012CRE	G256	K. pneun	NTU	2	R	R	R	2
2012CRE	G257	K. pneun	NTU	8	R	R	R	1
2012CRE	G258	K. pneun	NTU	4	R	R	R	0.5
2012CRE	G259	S. marce	NTU	4	R	R	R	2
2012CRE	G260	E. aeroge	NTU	1	I	R	R	0.25
2012CRE	G261	E. aeroge	NTU	2	R	R	R	0.5

2012CRE	G262	E. cloaca	NTU	1	I	R	R	0.5
2012CRE	G263	E. cloaca	NTU	1	I	R	R	0.5
2012CRE	G264	K. pneun	NTU	2	R	R	R	0.5
2012CRE	G265	K. pneun	NTU	1	I	R	R	1
2012CRE	G266	K. pneun	NTU	>128	R	R	R	32
2012CRE	G267	K. pneun	NTU	1	I	R	R	0.5
2012CRE	G268	K. pneun	NTU	2	R	R	R	2
2012CRE	G269	K. pneun	NTU	2	R	R	R	1
2012CRE	G270	K. pneun	NTU	1	I	R	R	1
2012CRE	G271	K. pneun	NTU	>128	R	R	R	16
2012CRE	G273	E. cloaca	NTU	2	R	R	R	2
2012CRE	G274	E. cloaca	NTU	2	R	R	R	0.5
2012CRE	G275	E. aeroge	NTU	4	R	R	R	4
2012CRE	G276	E. aeroge	NTU	1	I	R	R	0.5
2012CRE	G279	E. cloaca	NTU	1	I	R	R	0.25
2012CRE	G282	E. cloaca	NTU	2	R	R	R	0.5
2012CRE	G284	E. coli	NTU	1	I	R	R	0.25
2012CRE	G285	E. coli	NTU	8	R	R	R	0.5
2012CRE	G286	K. pneun	NTU	2	R	R	R	0.5
2012CRE	G287	K. pneun	NTU	2	R	R	R	0.5
2012CRE	G288	K. pneun	NTU	1	I	R	R	1
2012CRE	G289	S. marce	NTU	2	R	R	R	1
2012CRE	G290	S. marce	NTU	2	R	R	R	1
2012CRE	G291	K. pneun	NTU	4	R	R	R	0.5
2012CRE	G292	K. pneun	NTU	32	R	R	R	1
2012CRE	G293	E. cloaca	NTU	16	R	R	R	0.5
2012CRE	G294	K. pneun	NTU	128	R	R	R	8
2012CRE	G295	K. pneun	NTU	128	R	R	R	0.25
2012CRE	G296	K. pneun	NTU	2	R	R	R	1
2012CRE	G297	E. aeroge	NTU	8	R	R	R	4
2012CRE	G300	E. cloaca	NTU	1	I	R	R	1
2012CRE	G301	E. cloaca	NTU	2	R	R	R	1
2012CRE	G302	E. cloaca	NTU	1	I	R	R	0.25
2012CRE	G303	E. cloaca	NTU	1	I	R	R	0.5
2012CRE	G304	E. cloaca	NTU	1	I	R	R	0.25
2012CRE	G305	E. cloaca	NTU	2	R	R	R	0.25
2012CRE	G337	K. pneun	台大新竹	1	I	R	R	1
2012CRE	G342	K. pneun	台大新竹	>128	R	R	R	32
2012CRE	G346	P. stuarti	台大新竹	0.5	S	I	S	8
2012CRE	G354	K. pneun	台大新竹	2	R	R	R	0.5
2012CRE	G361	E. cloaca	台大新竹	4	R	R	R	0.5
2012CRE	G362	K. pneun	台大新竹	1	I	R	R	2
2012CRE	G367	K. pneun	台大新竹	1	I	R	R	1
2012CRE	G369	E. coli	台大新竹	2	R	R	R	1
2012CRE	G371	C. freunc	台大新竹	1	I	R	R	2
2012CRE	G372	E. cloaca	台大新竹	1	I	R	R	1

2012CRE	G455	E. cloaca	YTM	2	R	R	R	1
2012CRE	G456	E. cloaca	YTM	2	R	R	R	0.5
2012CRE	G457	E. coli	YTM	1	I	R	R	0.25
2012CRE	G459	E. cloaca	YTM	4	R	R	R	2
2012CRE	G460	E. cloaca	YTM	1	I	R	R	1
2012CRE	G461	K. pneum	YTM	8	R	R	R	0.5
2012CRE	G462	E. cloaca	YTM	2	R	R	R	1
2012CRE	G463	K. pneum	YTM	2	R	R	R	1
2012CRE	G465	E. cloaca	YTM	4	R	R	R	1
2012CRE	G466	E. cloaca	YTM	2	R	R	R	1
2012CRE	G467	K. pneum	YTM	2	R	R	R	1
2012CRE	G471	E. cloaca	YTM	2	R	R	R	0.5
2012CRE	G472	K. pneum	YTM	>128	R	R	R	64
2012CRE	G473	K. pneum	YTM	2	R	R	R	1
2012CRE	G474	K. pneum	YTM	>128	R	R	R	64
2012CRE	G477	E. cloaca	YTM	4	R	R	R	0.5
2012CRE	G478	E. cloaca	YTM	4	R	R	R	0.5
2012CRE	G479	K. pneum	YTM	2	R	R	R	1
2012CRE	G481	E. coli	YTM	32	R	R	R	2
2012CRE	G482	E. cloaca	YTM	2	R	R	R	1
2012CRE	G487	K. pneum	YTM	2	R	R	R	1
2012CRE	G488	P. rettge	YTM	16	R	R	R	8
2012CRE	G492	K. pneum	YTM	16	R	R	R	8
2012CRE	G494	E. coli	YTM	4	R	R	R	0.5
2012CRE	G496	K. pneum	YTM	2	R	R	R	1
2012CRE	G497	K. pneum	YTM	4	R	R	R	1
2012CRE	G498	C. freund	YTM	64	R	R	R	16
2012CRE	G501	K. pneum	YTM	2	R	R	R	0.5
2012CRE	G503	K. pneum	YTM	2	R	R	R	0.5
2012CRE	G504	K. pneum	YTM	2	R	R	R	0.5
2012CRE	G509	K. pneum	YTM	2	R	R	R	2
2012CRE	G511	K. pneum	YTM	64	R	R	R	8
2012CRE	G515	E. cloaca	YTM	1	I	R	R	1
2012CRE	G516	E. cloaca	YTM	1	I	R	R	0.5
2012CRE	G517	K. pneum	YTM	>128	R	R	R	64
2012CRE	G518	E. cloaca	YTM	4	R	R	R	0.5
2012CRE	G520	M. morg	YTM	>128	R	R	R	8
2012CRE	G521	K. pneum	YTM	2	R	R	R	1
2012CRE	G523	K. pneum	YTM	1	I	R	R	0.25
2012CRE	G524	E. cloaca	YTM	4	R	R	R	4
2012CRE	G525	E. cloaca	YTM	4	R	R	R	1
2012CRE	G526	E. aeroge	YTM	4	R	R	R	1
2012CRE	G530	K. pneum	YTM	2	R	R	R	1
2012CRE	G532	E. aeroge	YTM	2	R	R	R	2
2012CRE	G535	E. coli	YTM	8	R	R	R	0.5
2012CRE	G536	E. coli	YTM	4	R	R	R	0.5

2012CRE	G537	E. cloaca	YTM	0.5	S	I	S	2
2012CRE	G542	E. coli	YTM	8	R	R	R	1
2012CRE	G543	K. pneum	YTM	64	R	R	R	8
2012CRE	G545	K. pneum	YTM	2	R	R	R	0.5
2012CRE	G546	K. pneum	YTM	2	R	R	R	1
2012CRE	G549	K. pneum	YTM	4	R	R	R	0.5
2012CRE	G722	E. cloaca	YTM	4	R	R	R	1
2012CRE	G724	K. pneum	YTM	4	R	R	R	1
2012CRE	G727	K. pneum	YTM	8	R	R	R	1
2012CRE	G729	E. cloaca	YTM	4	R	R	R	2
2012CRE	G731	K. pneum	YTM	8	R	R	R	1
2012CRE	G732	E. coli	YTM	4	R	R	R	0.5
2012CRE	G734	K. pneum	YTM	32	R	R	R	4
2012CRE	G736	E. aeroge	YTM	1	I	R	R	1
2012CRE	G737	K. pneum	NTY	2	R	R	R	1
2012CRE	G738	K. pneum	NTY	2	R	R	R	0.5
2012CRE	G739	E. aeroge	NTY	1	I	R	R	0.5
2012CRE	G740	K. pneum	NTY	2	R	R	R	1
2012CRE	G741	K. pneum	NTY	1	I	R	R	0.5
2012CRE	G742	E. cloaca	NTY	4	R	R	R	0.5
2012CRE	G743	K. pneum	NTY	2	R	R	R	0.5
2012CRE	G744	E. coli	NTY	2	R	R	R	0.5
2012CRE	G745	E. cloaca	NTY	2	R	R	R	1
2012CRE	G746	C. freund	NTY	8	R	R	R	4
2012CRE	G747	K. pneum	NTY	4	R	R	R	1
2012CRE	G748	E. cloaca	NTY	4	R	R	R	1
2012CRE	G749	K. pneum	NTY	2	R	R	R	0.5
2012CRE	G750	K. pneum	NTY	2	R	R	R	0.25
2012CRE	G751	K. pneum	NTY	4	R	R	R	2
2012CRE	G753	K. pneum	NTY	2	R	R	R	0.5
2012CRE	G754	E. coli	NTY	2	R	R	R	1
2012CRE	G755	K. pneum	NTY	2	R	R	R	0.25
2012CRE	G756	K. pneum	NTY	2	R	R	R	1
2012CRE	G757	K. pneum	NTY	2	R	R	R	1
2012CRE	G758	K. pneum	NTY	128	R	R	R	32
2012CRE	G759	K. pneum	NTY	4	R	R	R	1
2012CRE	G760	K. pneum	NTY	8	R	R	R	1
2012CRE	G761	E. cloaca	NTY	2	R	R	R	0.5
2012CRE	G762	E. cloaca	NTY	8	R	R	R	4
2012CRE	G763	E. cloaca	NTY	1	I	R	R	0.25
2012CRE	G764	E. cloaca	NTY	8	R	R	R	1
2012CRE	G765	K. pneum	NTY	4	R	R	R	0.25
2012CRE	G766	K. pneum	NTY	2	R	R	R	1
2012CRE	G767	K. pneum	NTY	2	R	R	R	0.5
2012CRE	G768	K. pneum	NTY	4	R	R	R	0.5
2012CRE	G769	E. coli	NTY	128	R	R	R	16

2012CRE	G770	E. cloaca	NTY	4	R	R	R	0.5
2012CRE	G771	K. pneun	NTY	4	R	R	R	0.5
2012CRE	G772	E. cloaca	NTY	4	R	R	R	1
2012CRE	G773	E. coli	NTY	2	R	R	R	0.06
2012CRE	G774	K. pneun	NTY	2	R	R	R	1
2012CRE	G775	E. coli	NTY	4	R	R	R	2
2012CRE	G776	K. pneun	NTY	8	R	R	R	0.5
2012CRE	G777	K. pneun	NTY	128	R	R	R	8
2012CRE	G778	E. cloaca	NTY	2	R	R	R	0.5
2012CRE	G779	K. pneun	NTY	2	R	R	R	1
2012CRE	G780	K. pneun	NTY	4	R	R	R	1
2012CRE	G781	K. pneun	NTY	2	R	R	R	0.5
2012CRE	G782	K. pneun	NTY	16	R	R	R	1
2012CRE	G783	K. pneun	NTY	128	R	R	R	8
2012CRE	G784	K. pneun	NTY	4	R	R	R	0.5
2012CRE	G785	K. pneun	NTY	2	R	R	R	1
2012CRE	G786	E. cloaca	NTY	2	R	R	R	0.5
2012CRE	G787	E. cloaca	NTY	4	R	R	R	2
2012CRE	G788	K. pneun	NTY	16	R	R	R	0.5
2012CRE	G789	K. pneun	NTY	1	I	R	R	0.5
2012CRE	G790	E. cloaca	NTY	4	R	R	R	0.25
2012CRE	G791	K. pneun	NTY	16	R	R	R	0.5
2012CRE	G792	S. marce	NTY	32	R	R	R	8
2012CRE	G793	K. pneun	NTY	4	R	R	R	0.25
2012CRE	G794	E. coli	NTY	4	R	R	R	0.5
2012CRE	G717	E. coli	台大新竹	16	R	R	R	4
2012CRE	G272	E. cloaca	NTU	1	I	R	R	0.5
2012CRE	G306	E. cloaca	NTU	2	R	R	R	8
2012CRE	G307	E. coli	NTU	4	R	R	R	0.25
2012CRE	G308	E. coli	NTU	2	R	R	R	0.125
2012CRE	G310	K. pneun	NTU	2	R	R	R	0.25
2012CRE	G311	K. pneun	NTU	2	R	R	R	0.5
2012CRE	G312	K. pneun	NTU	2	R	R	R	0.25
2012CRE	G313	K. pneun	NTU	2	R	R	R	0.25
2012CRE	G314	K. pneun	NTU	32	R	R	R	2
2012CRE	G315	K. pneun	NTU	4	R	R	R	1
2012CRE	G316	K. pneun	NTU	2	R	R	R	0.25
2012CRE	G317	K. pneun	NTU	2	R	R	R	0.25
2012CRE	G318	K. pneun	NTU	2	R	R	R	2
2012CRE	G319	K. pneun	NTU	2	R	R	R	0.25
2012CRE	G320	S. marce	NTU	2	R	R	R	0.5
2012CRE	G321	K. pneun	NTU	2	R	R	R	0.5
2012CRE	G322	K. pneun	NTU	2	R	R	R	0.25
2012CRE	G576	E. aeroge	NTU	1	I	R	R	0.5
2012CRE	G577	E. cloaca	NTU	1	I	R	R	0.25
2012CRE	G578	E. cloaca	NTU	1	I	R	R	0.25

2012CRE	G579	E. cloaca	NTU	1	I	R	R	0.5
2012CRE	G580	E. cloaca	NTU	1	I	R	R	0.25
2012CRE	G581	E. coli	NTU	1	I	R	R	0.25
2012CRE	G582	E. coli	NTU	1	I	R	R	0.25
2012CRE	G583	K. pneum	NTU	2	R	R	R	2
2012CRE	G584	K. pneum	NTU	8	R	R	R	8
2012CRE	G586	K. pneum	NTU	1	I	R	R	0.5
2012CRE	G587	K. pneum	NTU	2	R	R	R	1
2012CRE	G588	K. pneum	NTU	64	R	R	R	4
2012CRE	G589	K. pneum	NTU	1	I	R	R	0.5
2012CRE	G590	K. pneum	NTU	8	R	R	R	1
2012CRE	G591	C. freund	NTU	1	I	R	R	0.5
2012CRE	G592	E. aeroge	NTU	1	I	R	R	0.5
2012CRE	G594	E. cloaca	NTU	1	I	R	R	0.5
2012CRE	G596	E. cloaca	NTU	2	R	R	R	1
2012CRE	G598	E. cloaca	NTU	2	R	R	R	0.25
2012CRE	G599	E. coli	NTU	1	I	R	R	0.25
2012CRE	G600	E. coli	NTU	8	R	R	R	0.5
2012CRE	G601	K. pneum	NTU	4	R	R	R	1
2012CRE	G602	K. pneum	NTU	1	I	R	R	1
2012CRE	G603	K. pneum	NTU	1	I	R	R	0.25
2012CRE	G604	K. pneum	NTU	2	R	R	R	1
2012CRE	G605	K. pneum	NTU	2	R	R	R	1
2012CRE	G606	K. pneum	NTU	1	I	R	R	0.5
2012CRE	G607	K. pneum	NTU	2	R	R	R	0.5
2012CRE	G608	E. aeroge	NTU	1	I	R	R	0.5
2012CRE	G609	E. cloaca	NTU	1	I	R	R	0.5
2012CRE	G453	K. pneum	NTU	2	R	R	R	0.5
2012CRE	G597	E. cloaca	NTU	2	R	R	R	0.25
2012CRE	G610	E. coli	NTU	4	R	R	R	0.25
2012CRE	G611	K. pneum	NTU	2	R	R	R	1
2012CRE	G612	K. pneum	NTU	2	R	R	R	0.5
2012CRE	G613	K. pneum	NTU	4	R	R	R	1
2012CRE	G614	K. pneum	NTU	4	R	R	R	0.25
2012CRE	G615	K. pneum	NTU	32	R	R	R	2
2012CRE	G616	K. pneum	NTU	2	R	R	R	1
2012CRE	G617	K. pneum	NTU	2	R	R	R	0.5
2012CRE	G618	K. pneum	NTU	4	R	R	R	1
2012CRE	G619	K. pneum	NTU	2	R	R	R	1
2012CRE	G620	K. pneum	NTU	4	R	R	R	1
2012CRE	G621	K. pneum	NTU	4	R	R	R	0.5
2012CRE	G622	K. pneum	NTU	1	I	R	R	0.25
2012CRE	G624	C. freund	NTU	1	I	R	R	0.25
2012CRE	G625	C. freund	NTU	4	R	R	R	1
2012CRE	G626	E. aeroge	NTU	1	I	R	R	2
2012CRE	G627	E. aeroge	NTU	1	I	R	R	0.5

2012CRE	G628	E. aeroge	NTU	1	I	R	R	2
2012CRE	G629	E. aeroge	NTU	2	R	R	R	1
2012CRE	G630	E. aeroge	NTU	8	R	R	R	2
2012CRE	G631	E. cloaca	NTU	8	R	R	R	1
2012CRE	G632	E. cloaca	NTU	2	R	R	R	0.5
2012CRE	G633	E. cloaca	NTU	8	R	R	R	2
2012CRE	G635	E. coli	NTU	2	R	R	R	2
2012CRE	G636	E. coli	NTU	8	R	R	R	1
2012CRE	G637	K. pneum	NTU	16	R	R	R	4
2012CRE	G638	K. pneum	NTU	2	R	R	R	1
2012CRE	G639	K. pneum	NTU	1	I	R	R	0.5
2012CRE	G640	K. pneum	NTU	>128	R	R	R	64
2012CRE	G641	K. pneum	NTU	1	I	R	R	0.25
2012CRE	G642	K. pneum	NTU	2	R	R	R	1
2012CRE	G643	K. pneum	NTU	2	R	R	R	0.5
2012CRE	G644	K. pneum	NTU	2	R	R	R	0.5
2012CRE	G645	K. pneum	NTU	4	R	R	R	0.5
2012CRE	G646	K. pneum	NTU	>128	R	R	R	16
2012CRE	G648	K. pneum	NTU	1	I	R	R	0.5
2012CRE	G649	K. pneum	NTU	2	R	R	R	0.5
2012CRE	G650	E. aeroge	NTU	2	R	R	R	0.25
2012CRE	G651	E. aeroge	NTU	32	R	R	R	8
2012CRE	G653	E. cloaca	NTU	4	R	R	R	0.25
2012CRE	G654	E. cloaca	NTU	2	R	R	R	0.5
2012CRE	G656	E. coli	NTU	4	R	R	R	0.125
2012CRE	G657	E. coli	NTU	2	R	R	R	0.25
2012CRE	G658	E. coli	NTU	32	R	R	R	4
2012CRE	G659	K. pneum	NTU	2	R	R	R	0.5
2012CRE	G660	K. pneum	NTU	1	I	R	R	0.5
2012CRE	G661	K. pneum	NTU	2	R	R	R	0.5
2012CRE	G662	K. pneum	NTU	1	I	R	R	0.5
2012CRE	G663	K. pneum	NTU	128	R	R	R	32
2012CRE	G664	K. pneum	NTU	16	R	R	R	2
2012CRE	G665	K. pneum	NTU	4	R	R	R	0.5
2012CRE	G666	S. marce	NTU	8	R	R	R	2
2012CRE	G667	S. marce	NTU	0.5	S	I	S	4
2012CRE	G668	C. freund	NTU	2	R	R	R	0.5
2012CRE	G669	E. aeroge	NTU	16	R	R	R	2
2012CRE	G670	E. aeroge	NTU	1	I	R	R	0.5
2012CRE	G671	E. cloaca	NTU	2	R	R	R	0.25
2012CRE	G672	E. coli	NTU	4	R	R	R	1
2012CRE	G673	E. coli	NTU	4	R	R	R	0.25
2012CRE	G674	E. coli	NTU	4	R	R	R	0.25
2012CRE	G675	K. pneum	NTU	1	I	R	R	1
2012CRE	G676	K. pneum	NTU	2	R	R	R	0.25
2012CRE	G677	K. pneum	NTU	4	R	R	R	0.25

2012CRE	G678	K. pneun	NTU	2	R	R	R	0.25
2012CRE	G679	K. pneun	NTU	16	R	R	R	0.5
2012CRE	G680	K. pneun	NTU	>128	R	R	R	32
2012CRE	G681	K. pneun	NTU	2	R	R	R	0.5
2012CRE	G682	K. pneun	NTU	1	I	R	R	0.25
2012CRE	G683	E. aeroge	NTU	1	I	R	R	0.5
2012CRE	G684	E. cloaca	NTU	1	I	R	R	0.25
2012CRE	G685	E. cloaca	NTU	8	R	R	R	0.5
2012CRE	G686	E. cloaca	NTU	8	R	R	R	0.25
2012CRE	G687	E. cloaca	NTU	8	R	R	R	0.25
2012CRE	G688	E. coli	NTU	1	I	R	R	0.25
2012CRE	G689	E. coli	NTU	128	R	R	R	16
2012CRE	G690	E. coli	NTU	8	R	R	R	1
2012CRE	G691	K. pneun	NTU	2	R	R	R	0.25
2012CRE	G692	K. pneun	NTU	2	R	R	R	0.5
2012CRE	G693	K. pneun	NTU	1	I	R	R	0.25
2012CRE	G694	K. pneun	NTU	2	R	R	R	0.5
2012CRE	G695	K. pneun	NTU	2	R	R	R	1
2012CRE	G696	K. pneun	NTU	2	R	R	R	0.25
2012CRE	G697	K. pneun	NTU	4	R	R	R	0.5
2012CRE	G698	K. pneun	NTU	2	R	R	R	0.25
2012CRE	G699	E. cloaca	NTU	4	R	R	R	2
2012CRE	G700	E. cloaca	NTU	4	R	R	R	0.5
2012CRE	G701	E. cloaca	NTU	4	R	R	R	2
2012CRE	G702	E. cloaca	NTU	8	R	R	R	0.5
2012CRE	G703	E. coli	NTU	2	R	R	R	1
2012CRE	G704	K. pneun	NTU	1	I	R	R	1
2012CRE	G705	K. pneun	NTU	8	R	R	R	1
2012CRE	G706	K. pneun	NTU	2	R	R	R	0.25
2012CRE	G707	K. pneun	NTU	4	R	R	R	1
2012CRE	G709	K. pneun	NTU	1	I	R	R	0.25
2012CRE	G710	K. pneun	NTU	2	R	R	R	0.5

IMP_C	MEM	MEM_C	FTX	FTX_C	FMX	FMX_C	FEP	FEP_C
I	0.125	S	>128	R	>128	R	2	S
I	0.06	S	16	R	128	R	0.25	S
R	1	S	64	R	32	I	0.25	S
S	0.06	S	32	R	128	R	32	R
I	0.06	S	16	R	64	R	0.25	S
S	0.06	S	>128	R	>128	R	2	S
I	0.25	S	64	R	>128	R	16	I
R	0.5	S	>128	R	>128	R	32	R
I	0.125	S	64	R	128	R	8	S
I	0.06	S	32	R	64	R	0.5	S
I	0.03	S	8	R	64	R	0.25	S
I	0.25	S	128	R	>128	R	8	S
S	0.06	S	64	R	128	R	1	S
S	0.06	S	64	R	128	R	1	S
S	0.06	S	32	R	128	R	1	S
S	0.06	S	128	R	>128	R	2	S
I	0.125	S	128	R	>128	R	1	S
R	0.5	S	128	R	32	I	8	S
S	0.06	S	32	R	128	R	0.25	S
I	0.125	S	4	R	>128	R	0.125	S
I	0.5	S	>128	R	>128	R	8	S
I	0.06	S	>128	R	>128	R	8	S
R	0.25	S	>128	R	>128	R	16	I
S	0.125	S	128	R	>128	R	2	S
S	0.125	S	64	R	>128	R	1	S
S	0.06	S	>128	R	>128	R	1	S
R	0.125	S	8	R	8	S	2	S
S	0.06	S	64	R	>128	R	0.5	S
I	0.125	S	>128	R	128	R	64	R
S	0.25	S	>128	R	>128	R	>128	R
S	0.06	S	>128	R	>128	R	32	R
S	0.06	S	>128	R	128	R	128	R
S	0.06	S	>128	R	>128	R	128	R
R	0.25	S	>128	R	>128	R	2	S
I	0.125	S	>128	R	>128	R	64	R
I	0.25	S	>128	R	>128	R	4	S
R	0.25	S	>128	R	>128	R	4	S
I	0.125	S	128	R	>128	R	4	S
S	0.125	S	128	R	>128	R	0.5	S
I	0.25	S	>128	R	>128	R	8	S
R	0.5	S	>128	R	>128	R	4	S
R	0.06	S	>128	R	>128	R	8	S
I	0.03	S	128	R	64	R	16	I
S	0.03	S	64	R	>128	R	16	I
R	0.125	S	>128	R	>128	R	32	R

I	0.06	S	>128	R	128	R	64	R
S	0.06	S	>128	R	>128	R	128	R
S	0.06	S	128	R	64	R	0.25	S
R	0.125	S	64	R	128	R	1	S
I	0.125	S	128	R	64	R	0.25	S
R	2	I	8	R	4	S	32	R
I	0.03	S	>128	R	128	R	32	R
R	0.125	S	>128	R	>128	R	64	R
R	0.06	S	16	R	>128	R	0.125	S
R	0.06	S	8	R	32	I	0.125	S
R	1	S	>128	R	>128	R	8	S
R	1	S	8	R	>128	R	8	S
S	0.06	S	64	R	128	R	32	R
I	0.06	S	64	R	128	R	32	R
R	128	R	>128	R	>128	R	>128	R
I	0.125	S	32	R	>128	R	1	S
R	8	R	>128	R	>128	R	>128	R
S	0.125	S	64	R	128	R	0.5	S
S	0.06	S	32	R	>128	R	1	S
S	0.06	S	32	R	>128	R	0.25	S
S	0.125	S	128	R	128	R	0.5	S
S	0.125	S	16	R	>128	R	0.125	S
I	1	R	32	R	>128	R	1	S
S	0.125	S	128	R	>128	R	0.5	S
S	0.5	R	>128	R	>128	R	2	S
S	0.06	S	32	R	>128	R	0.5	S
S	0.125	S	32	R	128	R	4	S
S	0.06	S	128	R	>128	R	1	S
S	0.06	S	>128	R	>128	R	2	I
S	0.06	S	32	R	>128	R	1	S
S	0.125	S	>128	R	>128	R	>128	R
S	0.06	S	>128	R	8	S	>128	R
S	0.25	S	>128	R	8	S	>128	R
S	0.5	R	128	R	>128	R	2	S
I	1	R	128	R	>128	R	2	S
S	0.125	S	>128	R	1	S	>128	R
S	0.125	S	>128	R	64	R	64	R
S	0.06	S	>128	R	128	R	64	R
S	0.125	S	128	R	64	R	32	R
S	0.06	S	128	R	64	R	16	I
S	0.125	S	>128	R	128	R	64	R
S	2	R	8	R	64	R	8	S
R	32	R	>128	R	>128	R	>128	R
S	0.125	S	16	R	128	R	16	S
I	0.25	S	>128	R	>128	R	>128	R
R	4	R	8	R	>128	R	2	S

S	0.06	S	>128	R	>128	R	128	R
S	0.06	S	8	R	64	R	0.25	S
S	0.06	S	8	R	64	R	0.25	S
S	0.125	S	128	R	>128	R	2	S
S	0.125	S	16	R	128	R	0.25	S
S	0.125	S	16	R	128	R	0.125	S
S	0.06	S	>128	R	>128	R	64	R
I	0.5	I	0.5	S	>128	R	0.06	S
S	0.125	S	>128	R	>128	R	64	R
S	0.125	S	128	R	>128	R	2	S
R	32	R	>128	R	>128	R	>128	R
S	0.06	S	>128	R	128	R	64	R
S	0.5	S	32	R	16	I	32	R
S	0.06	S	>128	R	>128	R	64	R
R	2	I	>128	R	>128	R	4	S
S	0.06	S	>128	R	128	R	32	R
S	<0.03	S	8	R	128	R	0.5	S
S	<0.03	S	32	R	32	I	0.5	S
S	0.25	S	>128	R	>128	R	2	S
R	8	R	>128	R	>128	R	>128	R
S	0.06	S	>128	R	>128	R	64	R
I	0.06	S	128	R	128	R	128	R
I	0.06	S	128	R	128	R	32	R
S	0.06	S	64	R	128	R	32	R
R	1	S	64	R	128	R	32	R
R	2	I	>128	R	>128	R	128	R
S	0.06	S	8	R	128	R	32	R
R	2	I	>128	R	128	R	128	R
S	0.125	S	>128	R	>128	R	0.5	S
S	0.25	S	>128	R	>128	R	8	S
S	0.06	S	>128	R	>128	R	4	S
S	0.06	S	>128	R	64	R	64	R
S	0.125	S	64	R	128	R	0.5	S
R	4	R	>128	R	>128	R	32	R
S	0.06	S	>128	R	128	R	128	R
S	0.25	S	128	R	>128	R	4	S
S	0.25	S	128	R	>128	R	1	S
S	0.06	S	32	R	64	R	2	S
R	4	R	>128	R	>128	R	>128	R
S	0.25	S	>128	R	>128	R	8	S
S	0.25	S	>128	R	2	S	>128	R
S	0.25	S	32	R	16	I	1	S
S	0.06	S	64	R	128	R	0.5	S
S	0.5	S	>128	R	8	S	>128	R
R	8	R	>128	R	>128	R	>128	R
R	0.25	S	128	R	>128	R	0.5	S

S	0.25	S	64	R	>128	R	8	S
S	0.25	S	>128	R	>128	R	4	S
S	0.125	S	>128	R	128	R	>128	R
S	0.125	S	>128	R	128	R	32	R
S	0.125	S	>128	R	128	R	>128	R
S	0.5	S	>128	R	>128	R	16	I
S	0.06	S	8	R	32	I	0.5	S
S	0.06	S	64	R	64	R	0.25	S
S	0.25	S	>128	R	128	R	128	R
S	0.125	S	>128	R	1	S	>128	R
I	0.125	S	64	R	64	R	0.25	S
S	0.125	S	32	R	64	R	0.5	S
S	0.06	S	>128	R	>128	R	4	S
S	0.125	S	>128	R	128	R	64	R
S	0.25	S	>128	R	>128	R	1	S
S	0.06	S	>128	R	128	R	64	R
S	0.06	S	>128	R	128	R	128	R
S	0.125	S	>128	R	0.5	S	>128	R
R	4	R	128	R	>128	R	64	R
S	0.5	S	128	R	8	S	64	R
S	0.125	S	>128	R	>128	R	2	S
R	4	R	128	R	>128	R	4	S
S	0.06	S	>128	R	1	S	>128	R
S	0.125	S	64	R	>128	R	1	S
S	0.06	S	64	R	128	R	8	S
I	2	I	>128	R	>128	R	>128	R
S	0.125	S	>128	R	32	I	>128	R
S	0.06	S	128	R	128	R	32	R
S	0.5	S	>128	R	>128	R	8	S
S	0.5	S	>128	R	>128	R	4	S
I	0.25	S	8	R	>128	R	0.25	S
S	0.125	S	128	R	>128	R	2	S
S	0.06	S	>128	R	128	R	128	R
R	4	R	>128	R	>128	R	>128	R
S	0.06	S	>128	R	128	R	64	R
S	0.25	S	16	R	>128	R	1	S
S	0.125	S	>128	R	>128	R	2	S
S	0.06	S	128	R	64	R	8	S
I	0.25	S	128	R	128	R	1	S
S	0.125	S	128	R	>128	R	8	S
S	0.06	S	128	R	32	I	16	I
S	0.125	S	>128	R	>128	R	4	S
R	8	R	>128	R	>128	R	>128	R
S	0.25	S	>128	R	>128	R	2	S
S	0.06	S	>128	R	128	R	1	S
S	0.25	S	>128	R	>128	R	>128	R

S	0.06	S	>128	R	>128	R	32	R
S	0.25	S	>128	R	>128	R	1	S
I	8	R	>128	R	>128	R	128	R
S	0.06	S	64	R	128	R	2	S
S	0.5	S	>128	R	>128	R	1	S
R	2	I	>128	R	>128	R	64	R
S	0.125	S	32	R	64	R	2	S
S	0.25	S	>128	R	>128	R	4	S
S	0.25	S	128	R	128	R	32	R
S	0.125	S	>128	R	>128	R	32	R
S	0.06	S	128	R	64	R	4	S
I	0.25	S	64	R	>128	R	0.5	S
S	0.125	S	2	I	>128	R	0.125	S
S	0.06	S	>128	R	>128	R	2	S
S	0.125	S	>128	R	8	S	>128	R
S	0.125	S	>128	R	>128	R	128	R
S	0.125	S	128	R	>128	R	0.5	S
S	0.06	S	>128	R	128	R	128	R
I	0.25	S	64	R	16	I	32	R
R	4	R	128	R	>128	R	4	S
S	0.125	S	64	R	64	R	128	R
S	0.25	S	>128	R	>128	R	2	S
S	0.125	S	>128	R	>128	R	8	S
S	0.125	S	>128	R	>128	R	4	S
S	0.25	S	>128	R	>128	R	128	R
S	0.06	S	2	I	64	R	0.5	S
S	<0.03	S	>128	R	64	R	1	S
S	0.06	S	>128	R	128	R	64	R
I	1	S	>128	R	>128	R	>128	R
S	0.06	S	64	R	>128	R	4	S
S	0.06	S	32	R	>128	R	2	S
I	0.5	S	8	R	>128	R	0.125	S
S	0.06	S	64	R	>128	R	0.25	S
S	0.125	S	>128	R	128	R	64	R
S	0.125	S	>128	R	>128	R	>128	R
I	2	I	128	R	64	R	128	R
S	0.06	S	8	R	64	R	0.25	S
S	0.125	S	64	R	64	R	64	R
S	0.125	S	64	R	>128	R	1	S
S	0.25	S	>128	R	>128	R	32	R
S	0.25	S	>128	R	>128	R	128	R
S	0.125	S	>128	R	>128	R	128	R
S	0.125	S	>128	R	>128	R	64	R
S	0.06	S	>128	R	64	R	64	R
S	0.5	S	32	R	64	R	8	S
S	0.06	S	>128	R	>128	R	2	S

S	0.125	S	>128	R	8	S	>128	R
S	0.125	S	64	R	>128	R	1	S
S	0.06	S	64	R	>128	R	16	I
S	0.125	S	>128	R	16	I	>128	R
S	0.25	S	128	R	>128	R	16	I
S	0.06	S	64	R	>128	R	16	I
R	4	R	8	R	>128	R	16	I
S	0.5	S	>128	R	>128	R	>128	R
S	0.125	S	64	R	>128	R	0.5	S
S	0.25	S	64	R	>128	R	4	S
S	0.125	S	64	R	>128	R	2	S
S	0.06	S	128	R	>128	R	0.25	S
S	0.125	S	4	R	>128	R	0.125	S
S	0.125	S	64	R	128	R	0.5	S
S	0.125	S	64	R	128	R	0.25	S
S	0.06	S	128	R	>128	R	32	R
R	2	I	64	R	>128	R	2	S
S	0.06	S	>128	R	>128	R	2	S
S	0.06	S	>128	R	128	R	64	R
S	0.125	S	16	R	128	R	0.25	S
I	0.5	S	>128	R	>128	R	128	R
S	0.25	S	128	R	>128	R	4	S
I	1	S	>128	R	>128	R	>128	R
S	<0.03	S	32	R	32	I	8	S
I	0.06	S	>128	R	128	R	64	R
S	0.06	S	>128	R	128	R	64	R
S	0.25	S	>128	R	128	R	128	R
S	0.06	S	128	R	64	R	64	R
S	0.125	S	>128	R	>128	R	32	R
S	0.06	S	64	R	128	R	0.5	S
I	1	S	64	R	>128	R	4	S
I	0.125	S	2	I	32	I	0.125	S
S	0.06	S	64	R	128	R	0.5	S
S	0.06	S	64	R	>128	R	0.5	S
R	1	S	128	R	>128	R	2	S
R	16	R	128	R	>128	R	2	S
S	0.5	S	>128	R	>128	R	8	S
S	0.125	S	128	R	>128	R	2	S
I	8	R	>128	R	>128	R	64	R
S	0.06	S	>128	R	>128	R	4	S
S	0.125	S	>128	R	>128	R	4	S
S	0.06	S	128	R	128	R	32	R
S	0.125	S	128	R	>128	R	4	S
R	16	R	128	R	64	R	32	R
S	0.125	S	>128	R	2	S	>128	R
S	0.06	S	128	R	128	R	64	R

S	0.06	S	128	R	64	R	64	R
S	0.06	S	64	R	128	R	64	R
S	0.06	S	32	R	64	R	8	S
S	0.125	S	64	R	>128	R	0.25	S
S	0.06	S	128	R	128	R	32	R
S	0.06	S	128	R	128	R	64	R
S	0.06	S	8	R	64	R	0.125	S
S	0.06	S	64	R	64	R	64	R
S	0.25	S	>128	R	>128	R	2	S
S	0.125	S	>128	R	>128	R	4	S
S	0.125	S	128	R	>128	R	1	S
S	0.125	S	1	S	128	R	0.06	S
I	0.06	S	128	R	64	R	0.25	S
S	0.06	S	128	R	128	R	0.5	S
S	0.25	S	64	R	>128	R	0.25	S
S	0.06	S	64	R	64	R	32	R
S	0.06	S	16	R	>128	R	0.125	S
S	0.25	S	>128	R	1	S	>128	R
S	0.125	S	64	R	128	R	0.25	S
S	0.5	S	>128	R	>128	R	1	S
I	0.06	S	32	R	64	R	8	S
S	0.25	S	128	R	>128	R	2	S
S	0.125	S	64	R	128	R	0.25	S
S	0.25	S	>128	R	2	S	64	R
I	0.5	S	>128	R	>128	R	4	S
I	0.25	S	128	R	>128	R	2	S
S	0.06	S	64	R	>128	R	1	S
R	2	I	>128	R	>128	R	4	S
R	16	R	>128	R	>128	R	>128	R
S	8	R	128	R	>128	R	32	R
R	4	R	64	R	>128	R	8	S
R	4	R	128	R	>128	R	2	S
I	0.25	S	>128	R	>128	R	>128	R
S	0.125	S	>128	R	1	S	>128	R
S	0.06	S	2	I	64	R	0.25	S
R	4	R	>128	R	>128	R	128	R
R	0.5	S	128	R	>128	R	0.5	S
I	2	I	128	R	32	I	128	R
S	1	S	>128	R	64	R	>128	R
S	0.125	S	128	R	>128	R	2	S
S	0.125	S	>128	R	>128	R	2	S
I	0.5	S	>128	R	>128	R	64	R
S	0.06	S	64	R	128	R	1	S
S	0.25	S	32	R	>128	R	2	S
S	0.125	S	16	R	>128	R	0.125	S
S	0.125	S	128	R	>128	R	1	S

S	0.25	S	16	R	>128	R	0.25	S
S	0.25	S	128	R	>128	R	2	S
I	0.25	S	64	R	128	R	0.25	S
S	0.125	S	>128	R	>128	R	0.5	S
S	0.06	S	64	R	>128	R	4	S
S	0.25	S	>128	R	128	R	>128	R
S	0.125	S	64	R	>128	R	8	S
S	0.25	S	128	R	>128	R	0.5	S
S	0.5	S	64	R	>128	R	32	R
R	2	I	>128	R	>128	R	128	R
S	0.125	S	32	R	128	R	0.125	S
S	0.125	S	8	R	>128	R	0.5	S
S	0.5	S	>128	R	128	R	>128	R
S	0.25	S	>128	R	>128	R	128	R
S	0.06	S	32	R	128	R	0.125	S
S	0.06	S	64	R	>128	R	1	S
S	0.06	S	>128	R	0.25	S	>128	R
S	0.06	S	8	R	128	R	1	S
S	0.06	S	>128	R	128	R	64	R
S	0.06	S	0.25	S	>128	R	0.125	S
R	8	R	>128	R	>128	R	>128	R
I	0.06	S	128	R	64	R	64	R
I	0.06	S	16	R	64	R	64	R
S	0.25	S	>128	R	>128	R	32	R
R	4	R	>128	R	>128	R	4	S
R	0.06	S	0.5	S	2	S	16	I
R	8	R	>128	R	>128	R	128	R
S	0.125	S	128	R	>128	R	16	I
R	64	R	>128	R	>128	R	32	R
R	0.125	S	0.06	S	2	S	0.06	S
S	0.125	S	>128	R	64	R	128	R
S	0.06	S	8	R	64	R	0.125	S
S	0.125	S	>128	R	>128	R	128	R
I	0.125	S	>128	R	64	R	64	R
S	0.125	S	32	R	>128	R	0.5	S
S	0.06	S	0.125	S	8	S	0.125	S
R	0.125	S	1	S	128	R	0.125	S
S	0.06	S	32	R	64	R	2	S
R	0.25	S	>128	R	>128	R	4	S
R	0.06	S	<0.03	S	8	S	32	R
S	0.125	S	32	R	32	I	0.5	S
I	0.125	S	128	R	>128	R	16	I
I	0.06	S	>128	R	64	R	64	R
R	64	R	>128	R	>128	R	128	R
R	64	R	>128	R	>128	R	128	R
R	64	R	>128	R	>128	R	128	R

S	0.06	S	>128	R	64	R	64	R	
S	0.25	S	128	R	>128	R	8	S	
S	0.06	S	>128	R	>128	R	2	S	
S	0.125	S	>128	R	>128	R	2	S	
R	128	R	>128	R	>128	R	>128	R	
R	128	R	>128	R	>128	R	128	R	
I	0.25	S	>128	R	>128	R	2	S	
I	0.125	S	>128	R	>128	R	128	R	
S	0.125	S	>128	R	128	R	128	R	
S	0.125	S	64	R	128	R	0.125	S	
S	0.25	S	128	R	>128	R	2	S	
R	0.125	S	64	R	>128	R	2	S	
I	0.06	S	>128	R	128	R	128	R	
I	0.5	S	>128	R	>128	R	64	R	
S	0.125	S	>128	R	>128	R	0.5	S	
S	0.25	S	128	R	>128	R	0.5	S	
S	0.125	S	>128	R	>128	R	64	R	
S	0.06	S	>128	R	64	R	>128	R	
S	0.125	S	>128	R	128	R	64	R	
S	0.125	S	>128	R	64	R	>128	R	
I	2	I	>128	R	>128	R	4	S	
S	0.06	S	4	R	32	I	0.06	S	
R	1	S	>128	R	>128	R	64	R	
I	0.5	S	16	R	32	I	0.125	S	
S	0.06	S	4	R	32	I	0.125	S	
S	0.125	S	>128	R	128	R	32	R	
R	1	S	128	R	>128	R	1	S	
S	0.5	S	128	R	>128	R	32	R	
S	0.125	S	>128	R	>128	R	16	I	
S	0.5	S	128	R	>128	R	2	S	
S	0.5	S	64	R	>128	R	16	I	
S	0.125	S	>128	R	>128	R	64	R	
S	0.06	S	64	R	128	R	0.5	S	
S	0.25	S	128	R	>128	R	2	S	
S	0.125	S	>128	R	>128	R	64	R	
I	0.125	S	16	R	>128	R	0.125	S	
S	0.25	S	128	R	>128	R	2	S	
S	0.06	S	>128	R	128	R	64	R	
S	0.125	S	64	R	>128	R	1	S	
S	0.125	S	64	R	>128	R	1	S	
I	0.06	S	128	R	>128	R	32	R	
R	16	R	>128	R	>128	R	>128	R	
S	0.25	S	>128	R	>128	R	2	S	
I	0.125	S	>128	R	>128	R	64	R	
R	32	R	>128	R	>128	R	16	I	
S	0.125	S	>128	R	>128	R	64	R	

S	0.06	S	32	R	128	R	0.5	S
S	0.06	S	>128	R	64	R	64	R
I	0.125	S	128	R	16	I	32	R
S	0.06	S	4	R	32	I	0.5	S
S	0.06	S	64	R	128	R	1	S
S	0.125	S	128	R	>128	R	1	S
R	4	R	>128	R	>128	R	4	S
I	0.25	S	>128	R	>128	R	64	R
I	0.25	S	>128	R	>128	R	2	S
R	2	I	>128	R	128	R	16	I
S	0.06	S	>128	R	128	R	64	R
S	0.125	S	128	R	>128	R	16	I
S	0.125	S	>128	R	128	R	64	R
I	0.5	S	>128	R	128	R	128	R
I	0.25	S	128	R	128	R	32	R
I	0.5	S	>128	R	>128	R	128	R
S	0.125	S	>128	R	>128	R	128	R
S	0.06	S	>128	R	>128	R	64	R
S	0.06	S	128	R	>128	R	2	S
S	0.125	S	8	R	128	R	0.25	S
I	0.125	S	>128	R	>128	R	2	S
S	0.5	S	64	R	>128	R	2	S
S	0.125	S	128	R	128	R	32	R
S	0.125	S	>128	R	128	R	32	R
R	2	I	>128	R	>128	R	1	S
S	0.125	S	128	R	128	R	0.5	S
I	0.125	S	>128	R	128	R	128	R
S	0.125	S	128	R	128	R	64	R
S	0.125	S	>128	R	>128	R	32	R
I	0.125	S	128	R	128	R	32	R
S	0.125	S	>128	R	>128	R	32	R
S	0.06	S	64	R	64	R	32	R
R	4	R	>128	R	>128	R	>128	R
S	0.06	S	128	R	>128	R	4	S
S	0.125	S	>128	R	>128	R	2	S
S	0.125	S	>128	R	128	R	64	R
S	0.125	S	>128	R	128	R	32	R
S	0.125	S	>128	R	>128	R	0.5	S
S	0.25	S	>128	R	2	S	128	R
I	0.5	S	>128	R	64	R	128	R
S	0.125	S	>128	R	>128	R	8	S
I	0.06	S	128	R	128	R	32	R
S	0.06	S	16	R	128	R	0.25	S
I	1	S	>128	R	8	S	>128	R
I	0.5	S	8	R	>128	R	0.5	S
S	0.06	S	4	R	32	I	1	S

S	0.06	S	>128	R	128	R	64	R
S	0.125	S	32	R	128	R	1	S
S	0.25	S	>128	R	128	R	32	R
S	0.25	S	128	R	128	R	128	R
I	0.25	S	128	R	>128	R	16	I
S	0.06	S	64	R	>128	R	1	S
R	0.5	S	>128	R	>128	R	4	S
S	0.06	S	128	R	128	R	32	R
S	0.125	S	>128	R	0.5	S	>128	R
I	0.25	S	1	S	>128	R	64	R
S	0.5	S	>128	R	>128	R	8	S
S	0.06	S	128	R	128	R	32	R
R	128	R	128	R	>128	R	64	R
S	0.125	S	>128	R	128	R	>128	R
S	0.06	S	4	R	>128	R	0.125	S
S	0.125	S	32	R	128	R	1	S
S	0.06	S	>128	R	>128	R	4	S
I	0.5	S	>128	R	>128	R	>128	R
S	0.125	S	32	R	>128	R	8	S
R	4	R	>128	R	>128	R	>128	R
S	0.125	S	16	R	128	R	16	S
R	0.25	S	>128	R	>128	R	128	R
S	0.125	S	64	R	>128	R	8	S
I	0.25	S	2	I	32	I	0.06	S
R	1	S	32	R	16	I	8	S
R	0.25	S	16	R	>128	R	0.5	S
S	0.125	S	>128	R	>128	R	1	S
R	0.125	S	64	R	64	R	16	I
S	0.06	S	128	R	128	R	64	R
I	0.125	S	>128	R	>128	R	1	S
S	0.06	S	>128	R	64	R	>128	R
R	64	R	>128	R	>128	R	128	R
I	0.125	S	128	R	128	R	2	S
S	0.06	S	>128	R	128	R	64	R
R	0.125	S	128	R	128	R	64	R
S	0.25	S	>128	R	4	S	>128	R
S	0.125	S	0.5	S	128	R	0.06	S
I	4	R	>128	R	16	I	>128	R
S	0.5	S	>128	R	>128	R	8	S
S	0.06	S	128	R	64	R	0.5	S
S	0.125	S	>128	R	1	S	>128	R
S	0.25	S	128	R	>128	R	4	S
S	0.125	S	128	R	>128	R	128	R
I	0.25	S	128	R	>128	R	16	I
R	2	I	32	R	>128	R	1	S
S	0.06	S	>128	R	128	R	32	R

S	0.125	S	>128	R	>128	R	8	S
S	0.125	S	>128	R	>128	R	32	R
S	0.125	S	>128	R	>128	R	1	S
I	0.06	S	128	R	128	R	64	R
S	0.125	S	>128	R	128	R	64	R
S	0.125	S	128	R	>128	R	4	S
S	0.25	S	128	R	>128	R	2	S
S	0.06	S	16	R	>128	R	0.25	S
S	0.25	S	64	R	128	R	2	S
S	0.125	S	8	R	>128	R	2	S
R	2	I	>128	R	>128	R	>128	R
R	1	S	>128	R	>128	R	8	S
S	0.125	S	128	R	>128	R	2	S
I	0.25	S	>128	R	128	R	64	S
I	0.125	S	4	R	128	R	0.125	S
S	0.25	S	>128	R	1	S	>128	R
R	4	R	>128	R	>128	R	>128	R
R	0.25	S	128	R	64	R	32	R
S	0.06	S	>128	R	>128	R	8	S
S	0.125	S	>128	R	>128	R	4	S
I	0.06	S	128	R	>128	R	16	I
R	0.06	S	>128	R	>128	R	4	S
R	0.25	S	>128	R	>128	R	>128	R
R	0.25	S	>128	R	>128	R	64	R
S	0.125	S	128	R	>128	R	8	S
S	<0.03	S	2	I	32	I	0.25	S
I	0.06	S	2	I	32	I	4	S
S	0.125	S	128	R	128	R	16	I
S	0.125	S	>128	R	64	R	64	R
S	0.125	S	128	R	128	R	32	R
S	0.125	S	>128	R	>128	R	64	R
S	0.125	S	128	R	128	R	32	R
S	0.25	S	>128	R	>128	R	>128	R
S	0.125	S	128	R	>128	R	64	R
S	0.06	S	>128	R	128	R	64	R
R	4	R	16	R	>128	R	0.25	S
S	0.06	S	64	R	>128	R	1	S
S	0.125	S	>128	R	>128	R	16	I
R	0.5	S	128	R	>128	R	2	S
S	0.06	S	8	R	>128	R	1	S
S	0.06	S	128	R	128	R	64	R
S	0.125	S	>128	R	64	R	32	R
S	0.125	S	32	R	128	R	1	S
S	0.25	S	>128	R	2	S	128	R
S	0.06	S	32	R	>128	R	4	S
S	0.125	S	32	R	>128	R	2	S

R	8	R	>128	R	>128	R	>128	R
S	0.5	S	>128	R	>128	R	32	R
S	0.06	S	128	R	128	R	64	R
I	0.25	S	32	R	128	R	0.25	S
R	0.125	S	128	R	64	R	0.25	S
S	0.125	S	>128	R	128	R	64	R
R	4	R	>128	R	>128	R	128	R
R	8	R	>128	R	32	I	>128	R
I	0.25	S	16	R	8	S	4	S
S	0.125	S	8	R	>128	R	1	S
R	0.5	S	>128	R	>128	R	128	R
S	0.25	S	>128	R	>128	R	8	S
R	4	R	>128	R	>128	R	2	S
S	0.125	S	32	R	128	R	1	S
R	4	R	>128	R	>128	R	8	S
S	0.5	S	32	R	>128	R	0.5	S
I	0.5	S	>128	R	>128	R	128	R
I	1	S	>128	R	>128	R	>128	R
S	0.5	S	>128	R	>128	R	128	R
S	0.125	S	16	R	128	R	0.25	S
I	0.125	S	128	R	128	R	128	R
S	<0.03	S	8	R	32	I	2	S
S	0.125	S	64	R	>128	R	1	S
R	4	R	64	R	>128	R	2	S
R	2	I	>128	R	>128	R	4	S
S	0.25	S	64	R	128	R	0.5	S
S	0.06	S	>128	R	>128	R	2	S
S	0.125	S	>128	R	>128	R	4	S
R	2	I	>128	R	>128	R	128	R
S	0.125	S	8	R	64	R	1	S
S	0.06	S	128	R	64	R	64	R
S	0.125	S	>128	R	64	R	128	R
S	0.25	S	2	I	128	R	1	S
I	0.25	S	64	R	>128	R	1	S
S	0.06	S	128	R	32	I	64	R
R	64	R	>128	R	>128	R	32	R
S	0.125	S	32	R	128	R	1	S
S	0.125	S	128	R	>128	R	1	S
S	0.25	S	>128	R	>128	R	32	R
S	0.125	S	64	R	128	R	1	S
S	0.06	S	128	R	>128	R	0.25	S
S	0.5	S	128	R	>128	R	2	S
S	0.125	S	128	R	>128	R	4	S
S	0.125	S	2	I	>128	R	0.06	S
S	0.125	S	128	R	>128	R	2	S
S	0.125	S	128	R	>128	R	0.5	S

S	0.25	S	16	R	128	R	0.5	S
S	0.06	S	>128	R	>128	R	32	R
S	0.125	S	4	R	128	R	0.06	S
S	0.06	S	>128	R	128	R	64	R
S	0.125	S	>128	R	2	S	64	R
S	0.125	S	128	R	128	R	32	R
S	0.25	S	128	R	>128	R	2	S
S	0.25	S	32	R	128	R	0.5	S
S	0.06	S	8	R	128	R	1	S
S	0.5	S	>128	R	>128	R	128	R
R	2	I	128	R	>128	R	8	S
S	0.06	S	32	R	>128	R	0.5	S
S	0.25	S	32	R	128	R	1	S
S	0.125	S	32	R	128	R	0.25	S
S	1	S	>128	R	>128	R	16	I
S	0.25	S	>128	R	>128	R	4	S
S	0.125	S	>128	R	>128	R	8	S
S	0.125	S	>128	R	>128	R	4	S
S	0.125	S	>128	R	>128	R	2	S
S	0.125	S	128	R	>128	R	4	S
S	0.125	S	>128	R	32	I	32	R
S	0.5	S	>128	R	8	S	>128	R
S	0.25	S	>128	R	16	I	64	R
S	0.06	S	128	R	64	R	32	R
S	0.06	S	128	R	128	R	64	R
R	16	R	>128	R	>128	R	>128	R
I	0.25	S	16	R	>128	R	0.25	S
S	0.25	S	16	R	128	R	0.125	S
S	0.06	S	8	R	64	R	0.5	S
S	0.125	S	>128	R	128	R	64	R
S	0.06	S	8	R	128	R	0.25	S
R	4	R	>128	R	>128	R	>128	R
S	0.06	S	64	R	64	R	0.5	S
R	4	R	>128	R	>128	R	8	S
S	0.125	S	128	R	>128	R	1	S
S	0.125	S	64	R	>128	R	0.5	S
S	0.25	S	128	R	>128	R	4	S
S	0.125	S	>128	R	128	R	>128	R
S	<0.03	S	32	R	64	R	0.125	S
R	32	R	>128	R	>128	R	32	R
S	0.06	S	128	R	128	R	64	R
S	0.125	S	32	R	128	R	16	I
S	0.25	S	>128	R	>128	R	>128	R
S	0.25	S	>128	R	>128	R	>128	R
S	0.06	S	>128	R	128	R	64	R
S	0.125	S	>128	R	32	I	2	S

S	0.06	S	>128	R	128	R	64	R	
R	32	R	>128	R	>128	R	32	R	
R	0.5	S	128	R	>128	R	0.5	S	
I	0.125	S	>128	R	>128	R	4	S	
I	8	R	>128	R	128	R	32	R	
S	0.125	S	128	R	>128	R	1	S	
S	0.06	S	8	R	>128	R	0.5	S	
S	0.125	S	64	R	128	R	0.5	S	
S	0.125	S	64	R	128	R	0.5	S	
S	0.25	S	64	R	128	R	2	S	
S	0.125	S	64	R	128	R	0.5	S	
I	0.5	S	>128	R	>128	R	1	S	
S	0.125	S	>128	R	>128	R	2	S	
S	0.125	S	0.5	S	128	R	0.25	S	
I	1	S	>128	R	>128	R	16	I	
S	0.06	S	32	R	>128	R	1	S	
S	0.5	S	>128	R	4	S	>128	R	
S	0.125	S	>128	R	64	R	128	R	
S	0.06	S	32	R	64	R	16	I	
S	0.06	S	128	R	64	R	64	R	
S	0.125	S	>128	R	64	R	64	R	
S	0.5	S	>128	R	>128	R	64	R	
R	4	R	>128	R	>128	R	>128	R	
S	0.06	S	>128	R	128	R	64	R	
I	0.125	S	8	R	>128	R	0.5	S	
S	<0.03	S	4	R	64	R	0.5	S	
S	<0.03	S	4	R	64	R	0.06	S	
S	0.06	S	>128	R	64	R	64	R	
R	2	I	128	R	64	R	64	R	
S	0.06	S	>128	R	128	R	1	S	
S	0.125	S	32	R	128	R	0.25	S	
S	0.125	S	64	R	>128	R	2	S	
S	0.06	S	8	R	128	R	0.25	S	
S	0.06	S	4	R	128	R	0.06	S	
S	0.25	S	>128	R	>128	R	4	S	
S	1	S	>128	R	>128	R	0.06	S	
S	0.06	S	>128	R	1	S	128	R	
S	0.06	S	>128	R	128	R	128	R	
S	0.125	S	32	R	128	R	0.25	S	
S	0.25	S	64	R	>128	R	16	I	
S	0.06	S	32	R	>128	R	0.25	S	
S	<0.03	S	4	R	64	R	0.25	S	
S	<0.03	S	4	R	64	R	0.5	S	
S	0.125	S	64	R	>128	R	1	S	
R	4	R	>128	R	>128	R	>128	R	
S	0.06	S	64	R	>128	R	0.125	S	

S	0.125	S	64	R	>128	R	0.5	S
S	0.125	S	16	R	>128	R	0.125	S
I	0.25	S	64	R	>128	R	0.25	S
S	0.06	S	32	R	>128	R	0.125	S
S	0.06	S	4	R	128	R	0.06	S
S	0.125	S	64	R	>128	R	1	S
R	4	R	>128	R	>128	R	>128	R
I	0.5	S	16	R	>128	R	0.125	S
R	0.5	S	128	R	>128	R	1	S
S	0.06	S	>128	R	128	R	64	R
S	0.125	S	>128	R	128	R	64	R
S	0.06	S	>128	R	>128	R	64	R
S	0.125	S	>128	R	128	R	128	R
S	0.06	S	16	R	128	R	0.125	S
S	0.25	S	16	R	32	I	4	S
S	0.25	S	>128	R	1	S	>128	R
S	0.06	S	64	R	128	R	0.5	S
S	0.25	S	32	R	128	R	8	S
I	1	S	>128	R	>128	R	128	R
S	0.25	S	64	R	64	R	8	S
I	1	S	>128	R	>128	R	>128	R
R	4	R	128	R	>128	R	64	R
S	0.25	S	128	R	>128	R	0.5	S
S	0.06	S	32	R	128	R	0.5	S
I	0.125	S	64	R	64	R	0.5	S
S	0.125	S	>128	R	128	R	128	R
S	0.25	S	>128	R	16	I	>128	R
S	0.06	S	128	R	>128	R	1	S
S	0.06	S	64	R	32	I	8	S
S	0.06	S	128	R	128	R	32	R
R	2	I	>128	R	>128	R	>128	R
S	0.125	S	128	R	>128	R	2	S
S	0.125	S	>128	R	>128	R	128	R
S	0.06	S	>128	R	128	R	32	R
S	0.06	S	>128	R	128	R	64	R
I	0.125	S	128	R	>128	R	64	R
S	0.125	S	128	R	>128	R	1	S
S	0.06	S	>128	R	>128	R	>128	R
S	0.06	S	>128	R	128	R	64	R
S	0.125	S	>128	R	128	R	32	R
S	0.06	S	32	R	128	R	1	S
S	0.06	S	>128	R	>128	R	64	R
S	0.125	S	128	R	>128	R	2	S
S	0.06	S	>128	R	128	R	1	S
R	16	R	128	R	>128	R	64	R
S	0.06	S	128	R	>128	R	8	S

I	0.25	S	8	R	>128	R	0.5	S
S	0.125	S	128	R	128	R	32	R
S	0.25	S	>128	R	>128	R	64	R
S	0.06	S	>128	R	>128	R	4	S
I	1	S	>128	R	>128	R	32	R
R	8	R	>128	R	>128	R	16	I
R	2	I	128	R	>128	R	32	R
S	0.06	S	64	R	>128	R	1	S
S	0.06	S	4	R	32	I	4	S
S	0.06	S	128	R	128	R	32	R
S	0.06	S	128	R	128	R	32	R
S	0.06	S	4	R	64	R	0.06	S
S	0.06	S	16	R	64	R	2	S
S	0.125	S	>128	R	128	R	64	R
S	0.06	S	128	R	128	R	64	R
S	0.06	S	128	R	128	R	32	R
S	0.06	S	64	R	64	R	16	I
S	0.06	S	16	R	64	R	0.125	S
S	0.06	S	32	R	32	I	4	S
S	<0.03	S	16	R	64	R	2	S
S	0.06	S	>128	R	128	R	16	I
S	<0.03	S	8	R	128	R	1	S
S	0.06	S	128	R	>128	R	32	R
S	0.125	S	64	R	>128	R	16	I
S	0.06	S	8	R	128	R	0.125	S
S	0.125	S	>128	R	128	R	128	R
S	0.06	S	2	I	64	R	0.125	S
S	0.06	S	64	R	64	R	4	S
S	0.06	S	32	R	128	R	0.25	S
S	0.06	S	>128	R	128	R	64	R
S	0.125	S	>128	R	>128	R	16	I
S	0.06	S	64	R	128	R	16	I
S	0.06	S	32	R	128	R	4	S
R	0.25	S	128	R	>128	R	0.5	S
S	0.06	S	>128	R	128	R	32	R
S	0.06	S	>128	R	>128	R	64	R
I	0.125	S	>128	R	>128	R	1	S
S	0.06	S	4	R	32	I	0.25	S
S	0.06	S	4	R	64	R	2	S
R	8	R	>128	R	>128	R	>128	R
I	0.125	S	>128	R	128	R	128	R
S	0.06	S	4	R	64	R	0.25	S
S	0.06	S	128	R	>128	R	32	R
S	0.125	S	>128	R	>128	R	>128	R
S	0.06	S	4	R	64	R	0.125	S
S	0.125	S	128	R	>128	R	8	S

S	0.06	S	>128	R	128	R	64	R
S	0.125	S	>128	R	128	R	32	R
S	0.06	S	>128	R	>128	R	8	S
S	0.125	S	>128	R	>128	R	64	R
S	0.06	S	64	R	64	R	16	I
S	0.06	S	128	R	128	R	64	R
S	0.125	S	>128	R	128	R	32	R
S	0.06	S	>128	R	128	R	64	R
S	0.06	S	>128	R	64	R	128	R
S	0.06	S	64	R	32	I	16	I
S	0.5	S	128	R	128	R	32	R
I	0.5	S	128	R	128	R	32	R
S	0.06	S	64	R	128	R	8	S
S	0.125	S	>128	R	>128	R	1	S
S	0.125	S	128	R	>128	R	8	S
R	2	I	>128	R	>128	R	>128	R
S	0.25	S	128	R	>128	R	2	S
S	0.06	S	128	R	128	R	64	R
S	0.125	S	128	R	>128	R	64	R
R	1	S	128	R	>128	R	32	R
S	0.125	S	128	R	>128	R	8	S
S	0.5	S	128	R	>128	R	16	I
S	0.06	S	128	R	128	R	64	R
S	0.125	S	>128	R	128	R	64	R
S	0.06	S	128	R	>128	R	8	S
R	2	I	128	R	>128	R	8	S
I	0.125	S	8	R	>128	R	0.125	S
S	0.125	S	64	R	128	R	0.25	S
S	0.125	S	>128	R	>128	R	4	S
S	0.125	S	>128	R	>128	R	64	R
S	0.06	S	128	R	64	R	16	I
S	0.125	S	32	R	64	R	16	I
S	0.25	S	>128	R	>128	R	>128	R
S	0.06	S	128	R	128	R	64	R
S	0.125	S	8	R	32	I	16	I
S	0.06	S	128	R	64	R	32	R
S	0.25	S	>128	R	>128	R	128	R
S	0.125	S	128	R	128	R	1	S
R	>128	R	>128	R	>128	R	128	R
S	0.5	S	128	R	>128	R	16	I
I	0.125	S	16	R	128	R	16	I
S	0.06	S	16	R	64	R	0.25	S
S	0.06	S	>128	R	128	R	64	R
I	1	S	64	R	128	R	2	S
S	0.06	S	64	R	128	R	2	S
S	0.06	S	128	R	>128	R	16	I

S	0.125	S	64	R	>128	R	0.25	S	
S	0.06	S	128	R	>128	R	1	S	
S	0.125	S	>128	R	>128	R	64	R	
S	0.06	S	32	R	128	R	0.5	S	
R	64	R	>128	R	>128	R	128	R	
S	<0.03	S	32	R	64	R	16	I	
I	0.25	S	>128	R	>128	R	16	I	
S	0.125	S	128	R	64	R	0.5	S	
S	0.125	S	32	R	128	R	0.5	S	
R	8	R	>128	R	>128	R	8	S	
I	0.25	S	16	R	>128	R	0.125	S	
S	0.06	S	>128	R	>128	R	2	S	
R	1	S	>128	R	>128	R	4	S	
S	0.125	S	32	R	128	R	0.5	S	
S	0.06	S	64	R	>128	R	1	S	
S	0.5	S	>128	R	>128	R	4	S	
S	<0.03	S	32	R	128	R	1	S	
S	0.125	S	128	R	>128	R	2	S	
S	0.125	S	>128	R	>128	R	128	R	
S	0.125	S	>128	R	128	R	64	R	
S	0.125	S	>128	R	128	R	1	S	
S	0.25	S	64	R	128	R	2	S	
S	0.5	S	8	R	64	R	0.5	S	
S	0.25	S	128	R	128	R	128	R	
S	0.5	S	128	R	64	R	16	I	
S	0.25	S	64	R	128	R	4	S	
R	4	R	128	R	>128	R	16	S	
S	4	R	>128	R	>128	R	128	R	
S	0.125	S	>128	R	128	R	>128	R	
R	0.5	S	128	R	>128	R	1	S	
S	0.25	S	0.5	S	16	I	8	S	
S	0.5	S	>128	R	>128	R	1	S	
S	0.06	S	64	R	128	R	0.5	S	
S	0.125	S	1	S	64	R	<0.03	S	
S	0.06	S	128	R	>128	R	2	S	
S	0.125	S	>128	R	>128	R	8	S	
S	0.06	S	8	R	32	I	0.5	S	
R	8	R	>128	R	>128	R	>128	R	
R	0.5	S	32	R	64	R	0.5	S	
S	0.125	S	>128	R	128	R	64	R	
S	0.25	S	>128	R	>128	R	1	S	
I	0.125	S	16	R	64	R	0.5	S	
S	0.06	S	64	R	32	I	0.25	S	
S	0.5	S	128	R	>128	R	2	S	
I	0.5	S	>128	R	16	I	8	S	
S	0.125	S	64	R	>128	R	0.25	S	

S	0.25	S	128	R	2	S	16	I
S	0.125	S	>128	R	>128	R	2	S
S	0.125	S	>128	R	>128	R	16	I
I	0.25	S	128	R	>128	R	32	R
S	0.125	S	8	R	>128	R	0.125	S
S	0.5	S	>128	R	>128	R	>128	R
S	0.5	S	>128	R	>128	R	32	R
S	0.06	S	8	R	128	R	0.5	S
S	0.5	S	128	R	>128	R	2	S
S	0.5	S	64	R	>128	R	8	S
S	0.06	S	128	R	128	R	32	R
S	0.25	S	>128	R	>128	R	64	R
R	>128	R	>128	R	>128	R	>128	R
S	0.06	S	128	R	128	R	64	R
R	128	R	>128	R	>128	R	>128	R
S	0.125	S	>128	R	>128	R	8	S
S	0.25	S	>128	R	>128	R	8	S
S	0.125	S	>128	R	16	I	64	R
I	4	R	>128	R	8	S	>128	R
S	0.125	S	128	R	>128	R	8	S
S	0.125	S	>128	R	>128	R	32	R
R	4	R	0.25	S	8	S	0.5	S
R	2	I	64	R	>128	R	1	S
S	0.125	S	>128	R	>128	R	64	R
S	0.06	S	8	R	128	R	0.5	S
S	0.125	S	128	R	128	R	32	R
R	8	R	128	R	>128	R	16	I
S	0.25	S	128	R	128	R	32	R
S	0.125	S	>128	R	128	R	32	R
S	0.125	S	>128	R	64	R	>128	R
I	0.125	S	128	R	128	R	32	R
R	4	R	>128	R	>128	R	>128	R
S	0.25	S	128	R	>128	R	0.5	S
S	0.06	S	64	R	128	R	0.25	S
R	>128	R	>128	R	>128	R	32	R
S	0.25	S	>128	R	>128	R	16	I
R	64	R	2	I	8	S	32	R
S	0.125	S	128	R	64	R	64	R
S	0.06	S	128	R	64	R	16	I
R	0.5	S	32	R	>128	R	0.25	S
S	0.5	S	32	R	>128	R	8	S
S	0.25	S	128	R	>128	R	8	S
S	0.06	S	>128	R	128	R	64	R
I	0.25	S	128	R	>128	R	4	S
S	0.25	S	>128	R	2	S	>128	R
S	0.25	S	>128	R	>128	R	64	R

I	0.25	S	128	R	16	I	4	S
S	0.25	S	64	R	>128	R	2	S
R	4	R	128	R	>128	R	2	S
S	0.125	S	>128	R	128	R	128	R
S	0.06	S	128	R	64	R	64	R
S	0.06	S	128	R	128	R	64	R
S	0.25	S	>128	R	>128	R	64	R
S	0.125	S	>128	R	128	R	64	R
S	0.5	S	>128	R	>128	R	>128	R
I	0.25	S	128	R	>128	R	16	I
S	0.25	S	>128	R	>128	R	128	R
S	0.125	S	64	R	>128	R	1	S
R	1	S	>128	R	>128	R	>128	R
S	0.06	S	16	R	64	R	0.5	S
S	0.125	S	8	R	128	R	0.125	S
S	0.125	S	>128	R	128	R	32	R
S	0.125	S	>128	R	128	R	>128	R
S	0.125	S	128	R	128	R	64	R
S	0.125	S	>128	R	64	R	16	I
S	0.5	S	64	R	>128	R	32	R
S	0.125	S	>128	R	>128	R	32	R
S	0.125	S	>128	R	>128	R	128	R
S	0.25	S	>128	R	>128	R	4	S
R	1	S	>128	R	>128	R	128	R
S	0.25	S	>128	R	>128	R	128	R
S	0.5	S	64	R	>128	R	16	I
S	0.125	S	>128	R	>128	R	32	R
S	0.125	S	>128	R	>128	R	128	R
I	0.125	S	8	R	128	R	0.25	S
S	0.125	S	>128	R	128	R	32	R
S	0.125	S	128	R	>128	R	2	S
S	0.06	S	128	R	32	I	32	R
S	0.125	S	>128	R	>128	R	64	R
S	0.125	S	>128	R	>128	R	16	I
R	16	R	64	R	>128	R	64	R
S	0.125	S	>128	R	128	R	32	R
S	0.25	S	64	R	>128	R	2	S
S	0.25	S	>128	R	>128	R	4	S
R	1	S	>128	R	>128	R	64	R
S	0.125	S	128	R	>128	R	2	S
S	0.5	S	128	R	>128	R	64	R
S	0.25	S	>128	R	128	R	128	R
S	0.125	S	64	R	128	R	8	S
S	0.125	S	>128	R	64	R	64	R
S	0.25	S	>128	R	>128	R	128	R
R	4	R	>128	R	>128	R	32	R

S	0.5	S	64	R	>128	R	16	I
S	0.125	S	>128	R	128	R	64	R
S	0.5	S	64	R	>128	R	8	S
S	0.25	S	>128	R	4	S	64	R
S	0.125	S	>128	R	>128	R	16	I
I	0.5	S	>128	R	>128	R	32	R
S	0.25	S	>128	R	>128	R	128	R
R	4	R	>128	R	>128	R	8	S
S	0.125	S	>128	R	>128	R	4	S
S	0.125	S	8	R	128	R	1	S
S	0.25	S	>128	R	>128	R	128	R
S	0.125	S	64	R	64	R	16	I
S	0.125	S	128	R	>128	R	16	I
R	4	R	>128	R	>128	R	128	R
S	0.25	S	>128	R	>128	R	128	R
S	0.125	S	128	R	128	R	32	R
S	0.06	S	>128	R	>128	R	0.5	S
I	0.5	S	64	R	>128	R	8	S
S	0.5	S	>128	R	>128	R	128	R
S	0.06	S	16	R	64	R	4	S
S	0.25	S	64	R	>128	R	16	I
S	0.5	S	>128	R	>128	R	128	R
R	8	R	>128	R	>128	R	2	S
S	0.25	S	>128	R	>128	R	128	R
S	0.25	S	>128	R	>128	R	>128	R
R	0.5	S	>128	R	>128	R	>128	R
S	0.125	S	64	R	128	R	0.5	S
R	0.5	S	128	R	64	R	64	R
S	0.25	S	>128	R	32	I	>128	R
S	0.25	S	>128	R	4	S	128	R
S	0.06	S	128	R	128	R	64	R
S	0.06	S	>128	R	64	R	64	R
S	0.125	S	>128	R	64	R	>128	R
S	0.06	S	8	R	64	R	1	S
I	1	S	128	R	>128	R	128	R
S	0.125	S	>128	R	>128	R	64	R
S	0.125	S	>128	R	128	R	64	R
S	0.06	S	>128	R	>128	R	64	R
I	0.125	S	128	R	>128	R	0.5	S
S	0.5	S	>128	R	64	R	>128	R
S	0.125	S	32	R	16	I	4	S
S	0.125	S	>128	R	128	R	64	R
S	0.125	S	>128	R	128	R	64	R
S	0.125	S	64	R	64	R	32	R
S	0.06	S	128	R	>128	R	2	S
S	0.06	S	128	R	>128	R	2	S

S	0.06	S	2	I	128	R	0.125	S
S	0.06	S	16	R	>128	R	0.25	S
S	0.06	S	>128	R	128	R	>128	R
S	0.06	S	>128	R	64	R	64	R
I	0.25	S	128	R	128	R	64	R
R	2	I	64	R	>128	R	16	I
S	0.06	S	8	R	64	R	0.125	S
S	0.125	S	128	R	>128	R	4	S
R	4	R	>128	R	>128	R	>128	R
S	0.06	S	32	R	64	R	0.125	S
S	0.5	S	64	R	>128	R	1	S
S	<0.03	S	128	R	>128	R	2	S
S	0.125	S	64	R	64	R	32	R
S	0.06	S	32	R	128	R	0.125	S
S	0.25	S	8	R	128	R	0.125	S
S	0.125	S	>128	R	>128	R	4	S
S	0.06	S	>128	R	>128	R	2	S
S	0.5	S	>128	R	>128	R	>128	R
S	0.25	S	16	R	>128	R	0.5	S
S	0.06	S	>128	R	32	I	32	R
S	0.125	S	>128	R	0.25	S	128	R
S	0.25	S	128	R	>128	R	4	S
S	0.125	S	64	R	128	R	0.5	S
S	0.06	S	4	R	128	R	0.25	S
S	0.06	S	>128	R	128	R	64	R
S	0.06	S	2	I	128	R	1	S
S	0.125	S	32	R	128	R	32	R
S	0.06	S	128	R	>128	R	2	S
S	0.125	S	>128	R	>128	R	4	S
S	0.25	S	>128	R	>128	R	128	R
S	0.06	S	>128	R	128	R	128	R
S	0.06	S	>128	R	128	R	64	R
S	0.125	S	>128	R	128	R	64	R
S	0.25	S	>128	R	1	S	128	R
I	1	S	>128	R	>128	R	>128	R
S	0.06	S	128	R	128	R	64	R
S	0.125	S	>128	R	32	I	>128	R
S	0.25	S	64	R	>128	R	0.5	S
S	0.06	S	>128	R	128	R	64	R
S	0.125	S	64	R	>128	R	0.5	S
S	0.25	S	>128	R	128	R	128	R
S	0.06	S	2	I	32	I	0.125	S
S	0.06	S	64	R	128	R	0.5	S
S	0.125	S	128	R	>128	R	4	S
I	0.06	S	16	R	128	R	0.25	S
S	0.125	S	64	R	>128	R	2	S

I	0.125	S	0.5	S	128	R	0.06	S
S	0.125	S	64	R	>128	R	1	S
I	0.5	S	>128	R	>128	R	4	S
S	0.5	S	>128	R	>128	R	16	I
S	0.125	S	32	R	>128	R	0.25	S
I	>128	R	>128	R	32	I	>128	R
I	0.125	S	32	R	>128	R	2	S
S	0.5	S	>128	R	>128	R	4	S
R	0.5	S	>128	R	>128	R	>128	R
S	0.125	S	128	R	128	R	32	R
S	0.06	S	8	R	16	I	1	S
R	128	R	>128	R	>128	R	128	R
S	0.06	S	8	R	64	R	0.25	S
S	0.06	S	>128	R	>128	R	32	R
S	0.06	S	>128	R	128	R	64	R
S	0.06	S	>128	R	128	R	64	R
S	0.5	S	64	R	32	I	4	S
R	8	R	>128	R	>128	R	>128	R
S	0.06	S	4	R	64	R	0.125	S
S	0.125	S	>128	R	>128	R	64	R
S	0.125	S	64	R	>128	R	1	S
R	4	R	128	R	>128	R	128	R
S	0.125	S	64	R	>128	R	2	S
S	0.125	S	64	R	128	R	0.25	S
S	0.125	S	>128	R	8	S	>128	R
S	0.125	S	64	R	>128	R	1	S
R	1	S	>128	R	>128	R	4	S
S	0.125	S	128	R	>128	R	64	R
S	0.06	S	128	R	>128	R	32	R
S	0.125	S	>128	R	32	I	128	R
S	0.125	S	8	R	>128	R	0.25	S
R	16	R	>128	R	64	R	32	R
I	1	S	>128	R	>128	R	>128	R
S	0.06	S	2	I	64	R	0.125	S
I	0.25	S	2	I	64	R	2	S
R	1	S	4	R	32	I	2	S
S	0.25	S	>128	R	>128	R	64	R
I	1	S	>128	R	64	R	4	S
S	0.06	S	32	R	>128	R	0.5	S
S	<0.03	S	64	R	>128	R	0.5	S
S	0.25	S	>128	R	>128	R	8	S
S	0.125	S	64	R	>128	R	1	S
S	0.125	S	>128	R	>128	R	2	S
S	0.06	S	>128	R	128	R	32	R
S	0.06	S	>128	R	128	R	64	R
S	0.06	S	>128	R	>128	R	64	R

S	0.125	S	128	R	64	R	32	R
S	0.5	S	>128	R	>128	R	64	R
R	16	R	>128	R	>128	R	>128	R
S	0.06	S	>128	R	128	R	32	R
S	<0.03	S	2	I	32	I	0.25	S
S	0.125	S	64	R	64	R	32	R
S	0.125	S	>128	R	>128	R	2	S
S	0.5	S	64	R	>128	R	1	S
S	0.5	S	>128	R	>128	R	2	S
S	0.25	S	>128	R	>128	R	8	S
S	0.06	S	>128	R	128	R	64	R
R	4	R	>128	R	>128	R	4	S
S	0.25	S	>128	R	>128	R	4	S
S	0.06	S	128	R	64	R	32	R
S	0.06	S	128	R	128	R	64	R
S	0.06	S	>128	R	0.25	S	32	R
S	0.06	S	128	R	128	R	64	R
S	0.125	S	>128	R	128	R	64	R
S	0.06	S	>128	R	128	R	32	R
S	0.125	S	>128	R	128	R	64	R
S	0.06	S	8	R	64	R	0.25	S
I	0.5	S	>128	R	>128	R	2	S
S	0.25	S	>128	R	>128	R	64	R
I	0.25	S	>128	R	>128	R	1	S
S	0.25	S	>128	R	>128	R	4	S
S	0.125	S	>128	R	64	R	64	R
S	0.06	S	128	R	64	R	32	R
S	0.25	S	>128	R	>128	R	>128	R
S	0.06	S	>128	R	128	R	64	R
S	0.25	S	128	R	>128	R	4	S
S	<0.03	S	64	R	32	I	0.25	S
S	0.125	S	32	R	>128	R	0.25	S

AMK1	AMK_C	GEN	GEN_C	CIP	CIP_C	COL	COL_C	TGC
2	S	32	R	0.25	S	0.5	S	1
4	S	1	S	1	S	1	S	0.25
2	S	2	S	2	I	1	S	0.25
1	S	0.5	S	0.125	S	0.5	S	0.5
1	S	0.5	S	0.06	S	0.5	S	0.5
1	S	0.25	S	<0.03	S	1	S	0.5
2	S	16	R	0.125	S	1	S	4
2	S	0.5	S	0.06	S	1	S	0.5
1	S	1	S	32	R	1	S	16
2	S	0.5	S	0.06	S	1	S	1
1	S	0.5	S	0.06	S	1	S	1
1	S	0.25	S	<0.03	S	1	S	0.5
2	S	0.5	S	0.06	S	1	S	1
2	S	0.5	S	0.06	S	1	S	1
2	S	0.5	S	0.06	S	1	S	1
1	S	1	S	0.5	S	1	S	4
1	S	0.25	S	0.06	S	1	S	0.5
1	S	1	S	2	I	32	R	2
2	S	0.5	S	<0.03	S	1	S	1
2	S	0.5	S	<0.03	S	1	S	0.5
2	S	0.5	S	<0.03	S	1	S	1
1	S	1	S	0.06	S	1	S	0.25
2	S	0.5	S	0.06	S	1	S	0.5
1	S	1	S	<0.03	S	1	S	8
2	S	0.5	S	1	S	1	S	0.5
1	S	0.25	S	<0.03	S	1	S	0.5
1	S	64	R	<0.03	S	1	S	1
1	S	1	S	0.25	S	4	R	1
2	S	0.5	S	0.25	S	1	S	0.5
2	S	64	R	>128	R	0.5	S	1
2	S	0.5	S	>128	R	0.5	S	1
2	S	64	R	128	R	0.5	S	0.5
2	S	0.5	S	2	I	0.5	S	1
1	S	0.5	S	0.5	S	0.5	S	0.5
2	S	1	S	>128	R	0.5	S	0.5
16	S	0.5	S	0.5	S	0.5	S	0.5
4	S	256	R	8	R	1	S	0.5
8	S	0.5	S	>128	R	0.5	S	1
2	S	0.5	S	0.06	S	0.5	S	0.5
4	S	0.5	S	0.06	S	0.5	S	1
2	S	1	S	>128	R	0.5	S	0.25
>128	R	256	R	128	R	1	S	0.5
>128	R	256	R	64	R	0.5	S	0.5
8	S	1	S	32	R	1	S	4
>128	R	256	R	128	R	1	S	1

>128	R	256	R	>128	R	0.5	S	0.5
>128	R	0.5	S	>128	R	1	S	1
1	S	0.25	S	0.5	S	0.5	S	1
1	S	0.25	S	<0.03	S	0.5	S	0.5
0.5	S	4	S	1	S	0.5	S	1
32	I	1	S	>128	R	0.5	S	1
>128	R	256	R	64	R	1	S	0.5
1	S	0.25	S	>128	R	1	S	1
2	S	0.5	S	1	S	1	S	0.5
1	S	32	R	0.5	S	0.5	S	1
2	S	128	R	0.125	S	0.5	S	1
16	S	2	S	>128	R	0.5	S	8
1	S	0.25	S	>128	R	1	S	0.5
1	S	0.25	S	>128	R	1	S	0.5
32	I	32	R	64	R	0.5	S	1
2	S	1	S	0.25	S	1	S	4
4	S	1	S	8	R	1	S	1
1	S	0.5	S	0.06	S	1	S	1
1	S	1	S	0.25	S	1	S	1
1	S	0.25	S	<0.03	S	1	S	0.5
1	S	0.25	S	<0.03	S	128	R	0.5
2	S	0.5	S	0.5	S	1	S	1
1	S	0.25	S	0.06	S	32	R	1
2	S	0.5	S	0.5	S	8	R	1
1	S	0.25	S	1	S	1	S	1
1	S	2	S	>128	R	1	S	8
1	S	0.25	S	16	R	1	S	8
1	S	0.25	S	<0.03	S	1	S	1
1	S	0.5	S	2	I	1	S	1
1	S	1	S	1	S	1	S	4
4	S	16	R	>128	R	1	S	1
8	S	64	R	>128	R	1	S	0.25
4	S	64	R	>128	R	0.5	S	1
2	S	1	S	0.5	S	1	S	0.25
4	S	1	S	0.25	S	0.5	S	0.25
>128	R	256	R	>128	R	1	S	1
1	S	0.25	S	>128	R	1	S	1
>128	R	256	R	128	R	1	S	1
1	S	16	R	>128	R	0.5	S	1
>128	R	256	R	128	R	1	S	1
1	S	4	S	>128	R	1	S	0.5
1	S	1	S	0.125	S	2	S	1
>128	R	0.5	S	128	R	0.5	S	1
>128	R	256	R	128	R	0.5	S	1
1	S	2	S	128	R	1	S	1
2	S	16	R	>128	R	32	R	4

1	S	4	S	32	R	1	S	1
2	S	1	S	64	R	0.5	S	2
2	S	0.5	S	128	R	1	S	1
32	I	32	R	0.25	S	0.5	S	0.25
1	S	1	S	0.125	S	0.5	S	1
1	S	1	S	4	R	1	S	0.5
4	S	16	R	8	R	1	S	1
2	S	8	I	0.06	S	1	S	0.5
2	S	64	R	128	R	0.5	S	0.5
2	S	16	R	<0.03	S	0.5	S	0.5
>128	R	256	R	16	R	0.5	S	0.5
>128	R	256	R	128	R	1	S	1
1	S	16	R	4	R	1	S	0.25
4	S	64	R	128	R	1	S	1
2	S	32	R	128	R	0.5	S	0.5
>128	R	256	R	>128	R	1	S	1
2	S	1	S	>128	R	1	S	0.5
2	S	1	S	1	S	1	S	0.25
2	S	16	R	0.5	S	0.5	S	4
>128	R	256	R	128	R	2	S	1
>128	R	0.5	S	128	R	1	S	1
2	S	16	R	>128	R	1	S	1
>128	R	256	R	128	R	2	S	1
1	S	1	S	4	R	1	S	4
2	S	2	S	16	R	1	S	0.5
>128	R	256	R	>128	R	1	S	1
2	S	256	R	>128	R	1	S	1
4	S	2	S	1	S	2	S	4
1	S	0.25	S	0.125	S	1	S	0.5
1	S	1	S	0.25	S	1	S	8
1	S	0.25	S	0.06	S	1	S	0.5
32	I	1	S	128	R	1	S	1
4	S	16	R	>128	R	1	S	1
>128	R	256	R	128	R	16	R	0.5
>128	R	256	R	>128	R	1	S	1
8	S	1	S	<0.03	S	0.5	S	0.25
4	S	2	S	64	R	0.5	S	0.5
16	S	256	R	128	R	0.5	S	1
>128	R	256	R	>128	R	1	S	1
2	S	16	R	128	R	>128	R	1
4	S	4	S	64	R	1	S	0.25
2	S	1	S	8	R	0.5	S	16
2	S	1	S	0.125	S	1	S	1
16	S	64	R	64	R	0.5	S	1
2	S	1	S	>128	R	1	S	1
2	S	1	S	<0.03	S	1	S	0.5

4	S	1	S	>128	R	1	S	0.5	
4	S	1	S	0.06	S	1	S	0.25	
4	S	1	S	64	R	1	S	1	
>128	R	256	R	>128	R	1	S	1	
>128	R	256	R	>128	R	1	S	1	
4	S	1	S	0.25	S	1	S	1	
1	S	8	I	>128	R	1	S	8	
2	S	256	R	0.06	S	>128	R	0.5	
2	S	1	S	>128	R	1	S	1	
>128	R	0.5	S	>128	R	0.5	S	1	
>128	R	0.25	S	32	R	1	S	1	
>128	R	16	R	64	R	1	S	1	
1	S	256	R	0.125	S	1	S	1	
>128	R	0.5	S	128	R	1	S	1	
4	S	2	S	1	S	1	S	0.5	
>128	R	256	R	>128	R	1	S	1	
2	S	0.25	S	>128	R	1	S	1	
1	S	0.25	S	>128	R	1	S	1	
>128	R	256	R	64	R	1	S	0.5	
2	S	1	S	1	S	1	S	1	
1	S	0.5	S	<0.03	S	1	S	0.5	
4	S	1	S	0.125	S	1	S	1	
8	S	64	R	>128	R	1	S	0.25	
4	S	1	S	0.125	S	1	S	1	
1	S	0.5	S	128	R	1	S	0.25	
2	S	1	S	128	R	1	S	1	
2	S	0.5	S	>128	R	1	S	0.5	
>128	R	256	R	128	R	1	S	1	
1	S	0.25	S	0.25	S	1	S	8	
1	S	0.5	S	0.25	S	1	S	8	
4	S	2	S	0.125	S	0.5	S	1	
2	S	0.5	S	128	R	0.5	S	0.5	
>128	R	16	R	>128	R	1	S	1	
>128	R	16	R	>128	R	1	S	1	
>128	R	256	R	128	R	1	S	1	
1	S	0.5	S	<0.03	S	128	R	0.5	
2	S	1	S	1	S	1	S	0.25	
>128	R	256	R	128	R	1	S	1	
2	S	1	S	2	I	1	S	1	
2	S	0.5	S	2	I	1	S	4	
2	S	128	R	>128	R	1	S	1	
8	S	128	R	4	R	0.5	S	1	
>128	R	256	R	>128	R	1	S	1	
1	S	1	S	2	I	64	R	2	
2	S	1	S	0.125	S	1	S	0.5	
1	S	1	S	128	R	1	S	1	

2	S	16	R	>128	R	1	S	0.5
0.5	S	8	I	64	R	0.5	S	1
>128	R	256	R	>128	R	>128	R	1
16	S	1	S	128	R	1	S	1
1	S	1	S	<0.03	S	0.5	S	1
1	S	2	S	2	I	1	S	1
32	I	2	S	>128	R	1	S	1
1	S	0.5	S	<0.03	S	1	S	1
2	S	0.5	S	>128	R	1	S	1
2	S	1	S	64	R	1	S	1
8	S	1	S	>128	R	1	S	1
2	S	1	S	2	I	1	S	1
2	S	0.5	S	0.06	S	128	R	0.5
1	S	1	S	<0.03	S	1	S	1
2	S	16	R	128	R	1	S	0.5
2	S	16	R	64	R	1	S	0.5
1	S	0.25	S	0.06	S	>128	R	1
>128	R	1	S	128	R	1	S	1
2	S	128	R	0.25	S	1	S	0.5
4	S	1	S	0.125	S	1	S	1
1	S	1	S	4	R	1	S	0.5
2	S	64	R	64	R	1	S	0.5
1	S	0.5	S	0.25	S	1	S	16
2	S	0.5	S	0.06	S	2	S	4
2	S	64	R	<0.03	S	1	S	0.25
2	S	1	S	>128	R	1	S	4
2	S	16	R	64	R	1	S	1
>128	R	256	R	128	R	1	S	1
>128	R	256	R	>128	R	1	S	1
8	S	1	S	8	R	1	S	1
2	S	1	S	128	R	1	S	2
1	S	1	S	<0.03	S	1	S	4
1	S	16	R	0.125	S	0.5	S	1
>128	R	1	S	128	R	1	S	4
4	S	64	R	32	R	1	S	0.5
>128	R	16	R	32	R	>128	R	4
1	S	0.5	S	0.5	S	1	S	0.5
1	S	0.5	S	0.25	S	>128	R	1
1	S	0.5	S	0.5	S	1	S	4
1	S	16	R	0.5	S	1	S	4
1	S	0.5	S	128	R	1	S	1
2	S	64	R	64	R	1	S	4
1	S	0.25	S	16	R	1	S	1
>128	R	256	R	128	R	1	S	1
1	S	2	S	1	S	1	S	0.5
1	S	0.5	S	<0.03	S	1	S	0.5

2	S	16	R	>128	R	1	S	1
2	S	1	S	2	I	1	S	4
16	S	2	S	128	R	1	S	1
>128	R	256	R	>128	R	1	S	1
2	S	1	S	64	R	1	S	0.5
1	S	1	S	>128	R	1	S	1
16	S	1	S	>128	R	1	S	1
4	S	64	R	16	R	1	S	1
2	S	64	R	2	I	1	S	1
2	S	1	S	0.25	S	1	S	8
1	S	0.25	S	0.125	S	>128	R	1
1	S	1	S	0.06	S	0.5	S	0.5
1	S	32	R	64	R	1	S	4
2	S	1	S	0.06	S	1	S	1
4	S	1	S	1	S	64	R	0.25
>128	R	256	R	128	R	1	S	1
8	S	16	R	64	R	1	S	0.5
2	S	1	S	0.125	S	1	S	0.5
>128	R	256	R	>128	R	1	S	1
2	S	0.5	S	0.5	S	>128	R	1
2	S	256	R	2	I	0.5	S	1
2	S	1	S	<0.03	S	0.5	S	0.5
2	S	16	R	>128	R	1	S	1
1	S	16	R	>128	R	0.5	S	1
1	S	0.5	S	>128	R	0.5	S	0.5
8	S	1	S	128	R	0.5	S	0.5
1	S	0.5	S	128	R	1	S	1
>128	R	256	R	128	R	1	S	1
8	S	2	S	4	R	0.5	S	1
2	S	0.5	S	0.125	S	1	S	0.25
2	S	0.5	S	2	I	0.5	S	8
2	S	1	S	1	S	1	S	1
2	S	0.5	S	0.125	S	1	S	1
2	S	0.5	S	0.125	S	0.5	S	1
2	S	1	S	0.125	S	1	S	0.5
2	S	0.5	S	0.06	S	0.5	S	0.25
2	S	128	R	2	I	1	S	1
1	S	0.5	S	<0.03	S	1	S	0.5
128	R	0.5	S	1	S	>128	R	0.5
1	S	0.5	S	0.5	S	1	S	0.5
4	S	128	R	32	R	1	S	0.5
2	S	1	S	>128	R	0.5	S	0.5
8	S	128	R	128	R	0.5	S	0.25
2	S	0.5	S	0.5	S	0.5	S	0.25
1	S	64	R	>128	R	0.5	S	1
1	S	16	R	128	R	0.5	S	1

1	S	32	R	128	R	0.5	S	1
>128	R	256	R	128	R	0.5	S	1
2	S	128	R	>128	R	0.5	S	1
2	S	0.5	S	<0.03	S	1	S	0.5
32	I	0.5	S	128	R	0.5	S	1
1	S	32	R	128	R	0.5	S	1
1	S	0.25	S	8	R	0.5	S	1
>128	R	256	R	128	R	0.5	S	1
1	S	8	I	<0.03	S	0.5	S	0.5
1	S	0.5	S	0.06	S	1	S	0.5
0.5	S	1	S	0.125	S	4	R	2
1	S	0.5	S	0.06	S	0.5	S	0.5
>128	R	256	R	16	R	0.5	S	1
1	S	0.5	S	0.06	S	0.5	S	0.5
1	S	0.5	S	0.25	S	0.5	S	0.25
1	S	0.5	S	>128	R	0.5	S	0.5
1	S	0.5	S	0.06	S	0.5	S	0.5
1	S	1	S	32	R	1	S	0.5
1	S	0.5	S	<0.03	S	1	S	0.5
1	S	0.5	S	<0.03	S	2	S	0.5
2	S	64	R	128	R	1	S	1
1	S	0.5	S	0.125	S	1	S	1
2	S	1	S	<0.03	S	64	R	1
2	S	0.5	S	32	R	1	S	0.25
2	S	1	S	0.06	S	1	S	0.5
2	S	1	S	0.125	S	1	S	0.5
2	S	1	S	0.25	S	1	S	1
2	S	1	S	0.25	S	1	S	4
4	S	16	R	32	R	1	S	2
8	S	4	S	64	R	1	S	1
16	S	1	S	1	S	0.5	S	1
2	S	1	S	2	I	1	S	1
>128	R	256	R	128	R	1	S	1
>128	R	256	R	64	R	1	S	1
4	S	1	S	>128	R	1	S	1
2	S	2	S	1	S	1	S	1
4	S	0.5	S	0.125	S	>128	R	1
>128	R	256	R	8	R	>128	R	1
>128	R	256	R	128	R	1	S	1
2	S	1	S	32	R	1	S	0.125
1	S	0.5	S	0.06	S	1	S	1
2	S	1	S	128	R	1	S	1
2	S	1	S	0.25	S	1	S	4
4	S	1	S	64	R	0.5	S	0.125
1	S	0.5	S	0.06	S	1	S	0.5
2	S	0.5	S	<0.03	S	1	S	0.5

2	S	4	S	64	R	1	S	1
2	S	1	S	0.125	S	4	R	0.5
1	S	0.5	S	<0.03	S	1	S	0.5
1	S	0.5	S	1	S	1	S	0.5
8	S	16	R	8	R	1	S	4
16	S	8	I	0.5	S	1	S	0.25
16	S	1	S	0.25	S	1	S	4
2	S	0.5	S	0.06	S	1	S	0.5
1	S	16	R	16	R	1	S	1
1	S	1	S	1	S	1	S	0.5
2	S	0.5	S	0.06	S	1	S	0.5
1	S	0.5	S	1	S	1	S	1
8	S	128	R	32	R	1	S	4
>128	R	256	R	0.5	S	1	S	1
1	S	0.5	S	0.06	S	2	S	0.5
2	S	0.5	S	<0.03	S	1	S	0.5
4	S	1	S	4	R	1	S	0.25
8	S	64	R	32	R	1	S	0.5
1	S	16	R	128	R	1	S	1
1	S	16	R	2	I	1	S	4
1	S	32	R	>128	R	64	R	1
0.5	S	32	R	128	R	1	S	1
2	S	16	R	0.5	S	1	S	1
2	S	64	R	64	R	1	S	0.5
1	S	1	S	4	R	1	S	8
2	S	1	S	0.25	S	>128	R	2
1	S	8	I	>128	R	0.5	S	8
2	S	8	I	0.5	S	1	S	0.25
>128	R	256	R	>128	R	1	S	1
2	S	16	R	<0.03	S	0.5	S	0.5
1	S	32	R	128	R	0.5	S	1
2	S	16	R	2	I	1	S	1
16	S	256	R	16	R	1	S	1
0.5	S	16	R	>128	R	1	S	2
2	S	1	S	0.5	S	1	S	0.25
1	S	1	S	<0.03	S	1	S	0.5
1	S	0.5	S	0.06	S	1	S	1
32	I	1	S	<0.03	S	2	S	1
1	S	256	R	32	R	64	R	1
2	S	0.5	S	<0.03	S	>128	R	0.5
2	S	64	R	1	S	1	S	1
8	S	256	R	8	R	1	S	1
>128	R	256	R	128	R	1	S	1
1	S	64	R	>128	R	1	S	1
2	S	0.25	S	>128	R	1	S	1
1	S	0.5	S	64	R	16	R	1

>128	R	256	R	128	R	1	S	1
16	S	0.5	S	32	R	1	S	1
1	S	0.5	S	0.125	S	1	S	0.5
1	S	4	S	0.06	S	1	S	1
>128	R	256	R	>128	R	0.5	S	0.5
1	S	64	R	>128	R	1	S	1
8	S	1	S	128	R	1	S	0.5
1	S	1	S	128	R	1	S	0.5
1	S	16	R	128	R	1	S	0.5
8	S	4	S	128	R	1	S	0.5
2	S	1	S	>128	R	0.5	S	0.5
>128	R	256	R	128	R	0.5	S	1
>128	R	256	R	128	R	1	S	1
1	S	1	S	128	R	1	S	1
1	S	0.5	S	0.125	S	1	S	0.5
1	S	0.5	S	<0.03	S	>128	R	1
1	S	16	R	16	R	1	S	0.5
8	S	16	R	16	R	1	S	0.5
>128	R	256	R	128	R	1	S	1
>128	R	256	R	128	R	1	S	1
2	S	0.5	S	128	R	1	S	1
2	S	0.5	S	0.5	S	1	S	1
16	S	16	R	128	R	1	S	0.25
8	S	1	S	1	S	1	S	1
1	S	0.5	S	0.5	S	1	S	1
>128	R	256	R	128	R	1	S	1
2	S	0.5	S	0.125	S	1	S	1
2	S	2	S	32	R	1	S	8
>128	R	16	R	128	R	1	S	0.5
2	S	128	R	128	R	0.5	S	1
1	S	128	R	8	R	1	S	1
1	S	0.5	S	128	R	1	S	1
2	S	0.5	S	<0.03	S	1	S	1
2	S	0.5	S	0.25	S	32	R	1
>128	R	256	R	128	R	1	S	1
2	S	0.5	S	64	R	32	R	1
2	S	64	R	32	R	1	S	0.5
1	S	64	R	128	R	1	S	1
4	S	1	S	64	R	1	S	0.25
4	S	128	R	32	R	1	S	0.5
1	S	0.5	S	128	R	2	S	1
>128	R	256	R	128	R	1	S	0.5
2	S	1	S	<0.03	S	0.5	S	0.25
1	S	1	S	128	R	1	S	1
2	S	2	S	128	R	0.5	S	0.5
2	S	0.5	S	128	R	1	S	1

2	S	1	S	0.06	S	1	S	1
2	S	64	R	128	R	1	S	1
4	S	2	S	128	R	1	S	0.25
>128	R	256	R	64	R	1	S	1
2	S	64	R	64	R	1	S	0.25
>128	R	16	R	>128	R	1	S	0.5
4	S	16	R	128	R	1	S	1
>128	R	256	R	128	R	1	S	1
2	S	1	S	128	R	1	S	1
4	S	128	R	4	R	1	S	0.5
>128	R	256	R	128	R	1	S	1
2	S	256	R	32	R	1	S	1
>128	R	256	R	128	R	1	S	1
8	S	8	I	32	R	1	S	0.5
1	S	64	R	128	R	1	S	1
8	S	1	S	>128	R	1	S	0.25
8	S	1	S	>128	R	1	S	1
>128	R	256	R	>128	R	1	S	1
2	S	0.5	S	0.06	S	1	S	0.5
>128	R	256	R	64	R	2	S	1
>128	R	16	R	>128	R	1	S	2
4	S	128	R	64	R	1	S	0.25
2	S	1	S	128	R	1	S	0.5
>128	R	16	R	128	R	1	S	0.5
8	S	1	S	1	S	>128	R	1
2	S	0.5	S	16	R	1	S	1
1	S	1	S	128	R	1	S	0.5
4	S	1	S	32	R	1	S	1
1	S	0.5	S	128	R	1	S	1
>128	R	256	R	>128	R	1	S	1
1	S	0.5	S	128	R	1	S	1
1	S	128	R	2	I	0.5	S	0.5
>128	R	256	R	>128	R	1	S	1
2	S	128	R	128	R	1	S	0.5
1	S	0.5	S	0.06	S	1	S	0.5
1	S	64	R	128	R	1	S	1
>128	R	256	R	128	R	1	S	1
1	S	0.5	S	0.125	S	1	S	0.5
1	S	4	S	128	R	1	S	16
8	S	1	S	4	R	4	R	2
2	S	1	S	64	R	0.5	S	0.25
1	S	64	R	128	R	1	S	1
1	S	1	S	32	R	1	S	1
>128	R	256	R	>128	R	1	S	1
1	S	0.5	S	0.125	S	>128	R	1
>128	R	256	R	128	R	1	S	1

>128	R	256	R	128	R	1	S	1
2	S	1	S	0.25	S	1	S	2
1	S	0.5	S	128	R	1	S	1
1	S	32	R	128	R	1	S	1
8	S	4	S	2	I	1	S	0.5
1	S	0.5	S	<0.03	S	1	S	1
4	S	2	S	64	R	1	S	0.25
1	S	16	R	>128	R	1	S	4
4	S	1	S	16	R	0.5	S	4
2	S	1	S	128	R	0.5	S	1
2	S	1	S	0.06	S	0.5	S	0.5
>128	R	256	R	128	R	1	S	1
2	S	0.5	S	128	R	1	S	1
1	S	32	R	0.5	S	0.5	S	0.25
1	S	0.5	S	0.06	S	1	S	1
1	S	0.5	S	1	S	>128	R	0.5
1	S	0.5	S	0.06	S	1	S	0.5
2	S	1	S	>128	R	1	S	2
1	S	1	S	2	I	1	S	8
4	S	1	S	8	R	1	S	0.5
1	S	16	R	128	R	1	S	0.5
1	S	1	S	128	R	0.5	S	0.5
32	I	16	R	2	I	1	S	2
2	S	16	R	8	R	4	R	0.5
>128	R	256	R	16	R	1	S	0.5
1	S	16	R	1	S	0.5	S	1
2	S	1	S	>128	R	1	S	8
2	S	1	S	128	R	>128	R	1
2	S	64	R	128	R	>128	R	1
4	S	1	S	0.06	S	0.5	S	0.25
2	S	16	R	128	R	0.5	S	0.25
1	S	0.5	S	64	R	1	S	0.5
4	S	16	R	64	R	0.5	S	0.25
1	S	32	R	128	R	1	S	1
1	S	32	R	128	R	>128	R	1
>128	R	256	R	8	R	0.5	S	1
1	S	0.5	S	0.06	S	1	S	0.5
2	S	1	S	0.5	S	0.5	S	1
2	S	0.5	S	0.25	S	0.5	S	0.5
>128	R	256	R	16	R	1	S	1
2	S	1	S	32	R	1	S	8
2	S	0.5	S	32	R	1	S	0.25
16	S	256	R	2	I	1	S	1
1	S	64	R	4	R	1	S	0.5
2	S	4	S	2	I	>128	R	1
>128	R	256	R	128	R	1	S	1

1	S	1	S	0.06	S	1	S	1
>128	R	256	R	128	R	1	S	1
2	S	1	S	>128	R	1	S	1
>128	R	256	R	128	R	1	S	1
>128	R	256	R	128	R	1	S	1
>128	R	0.5	S	>128	R	1	S	1
1	S	16	R	128	R	1	S	1
1	S	0.5	S	128	R	1	S	1
2	S	1	S	0.06	S	1	S	0.25
2	S	0.5	S	128	R	32	R	1
>128	R	256	R	>128	R	1	S	1
4	S	1	S	64	R	0.5	S	0.25
2	S	256	R	<0.03	S	0.5	S	0.25
2	S	16	R	0.25	S	0.5	S	1
1	S	1	S	16	R	1	S	1
2	S	16	R	>128	R	0.5	S	1
>128	R	256	R	>128	R	16	R	1
16	S	1	S	0.125	S	>128	R	1
1	S	0.5	S	2	I	0.5	S	0.5
1	S	0.5	S	0.5	S	0.5	S	1
1	S	16	R	1	S	0.25	S	1
1	S	0.5	S	4	R	0.5	S	0.5
2	S	1	S	4	R	0.5	S	1
4	S	0.5	S	128	R	0.5	S	1
1	S	0.5	S	0.5	S	0.5	S	0.5
2	S	1	S	>128	R	0.5	S	1
>128	R	16	R	1	S	0.5	S	1
2	S	0.5	S	64	R	0.5	S	1
1	S	16	R	64	R	0.5	S	1
1	S	32	R	128	R	0.5	S	1
1	S	64	R	128	R	0.5	S	1
1	S	32	R	128	R	0.5	S	1
1	S	0.5	S	128	R	0.5	S	1
1	S	64	R	128	R	128	R	1
1	S	32	R	128	R	0.5	S	1
4	S	16	R	8	R	>128	R	0.5
2	S	128	R	0.5	S	0.5	S	0.25
1	S	16	R	1	S	0.5	S	1
8	S	2	S	8	R	0.5	S	1
1	S	1	S	8	R	0.5	S	16
>128	R	256	R	128	R	0.5	S	1
1	S	64	R	128	R	0.5	S	1
1	S	0.5	S	0.5	S	0.5	S	1
2	S	128	R	64	R	0.5	S	0.25
8	S	8	I	128	R	0.5	S	2
2	S	1	S	2	I	1	S	0.5

1	S	16	R	128	R	0.5	S	1
1	S	16	R	64	R	0.5	S	0.5
0.5	S	32	R	128	R	0.5	S	1
2	S	1	S	0.06	S	0.5	S	0.5
2	S	16	R	0.5	S	0.5	S	0.25
1	S	16	R	128	R	0.5	S	0.5
1	S	1	S	2	I	1	S	1
1	S	128	R	>128	R	2	S	1
1	S	2	S	2	I	0.5	S	0.5
1	S	16	R	32	R	1	S	0.5
1	S	32	R	128	R	0.5	S	1
1	S	1	S	0.25	S	0.5	S	8
2	S	4	S	128	R	>128	R	1
8	S	64	R	32	R	0.5	S	0.5
2	S	1	S	2	I	0.5	S	0.5
1	S	1	S	0.25	S	1	S	0.5
1	S	32	R	128	R	0.5	S	1
1	S	64	R	128	R	0.5	S	1
0.5	S	16	R	128	R	0.5	S	0.5
2	S	1	S	0.06	S	0.5	S	0.5
>128	R	256	R	128	R	0.5	S	1
16	S	32	R	128	R	0.5	S	1
4	S	256	R	0.06	S	1	S	0.25
2	S	1	S	0.06	S	0.5	S	0.5
2	S	1	S	0.25	S	1	S	0.25
2	S	0.5	S	0.25	S	0.5	S	0.5
1	S	0.5	S	<0.03	S	1	S	1
1	S	1	S	0.125	S	1	S	4
1	S	32	R	>128	R	0.5	S	1
2	S	1	S	16	R	1	S	0.5
2	S	0.5	S	128	R	1	S	1
>128	R	256	R	64	R	1	S	0.5
2	S	1	S	0.25	S	0.5	S	4
2	S	16	R	4	R	0.5	S	1
>128	R	256	R	128	R	1	S	1
8	S	8	I	64	R	1	S	1
2	S	1	S	4	R	0.5	S	1
1	S	0.25	S	0.06	S	64	R	0.5
16	S	128	R	0.5	S	0.5	S	0.5
8	S	0.5	S	0.06	S	>128	R	0.5
2	S	0.5	S	<0.03	S	64	R	0.5
0.5	S	0.25	S	0.125	S	0.5	S	1
1	S	0.5	S	0.5	S	0.5	S	0.5
1	S	0.5	S	1	S	1	S	0.5
1	S	0.5	S	1	S	1	S	0.5
1	S	0.5	S	0.125	S	64	R	0.5

2	S	1	S	1	S	0.5	S	0.5	
2	S	1	S	>128	R	0.5	S	0.5	
1	S	0.25	S	1	S	0.5	S	0.5	
>128	R	256	R	128	R	0.5	S	1	
1	S	0.5	S	64	R	0.5	S	1	
0.5	S	1	S	64	R	0.5	S	1	
1	S	16	R	8	R	0.5	S	1	
1	S	16	R	1	S	1	S	1	
4	S	128	R	4	R	0.5	S	1	
4	S	2	S	<0.03	S	0.5	S	0.5	
2	S	1	S	0.06	S	0.5	S	0.5	
1	S	0.5	S	0.06	S	0.5	S	1	
4	S	1	S	8	R	0.5	S	1	
2	S	0.5	S	<0.03	S	0.5	S	1	
1	S	0.5	S	0.06	S	0.5	S	1	
2	S	0.5	S	<0.03	S	0.5	S	1	
1	S	0.25	S	0.06	S	1	S	0.5	
0.5	S	0.25	S	0.5	S	0.5	S	0.5	
1	S	1	S	16	R	1	S	2	
8	S	1	S	<0.03	S	1	S	0.5	
2	S	1	S	64	R	0.5	S	0.5	
1	S	128	R	128	R	0.5	S	1	
1	S	0.5	S	32	R	1	S	1	
>128	R	256	R	128	R	1	S	1	
2	S	0.5	S	128	R	1	S	1	
0.5	S	1	S	16	R	1	S	4	
1	S	1	S	4	R	1	S	1	
1	S	64	R	0.5	S	0.5	S	1	
1	S	64	R	1	S	1	S	1	
1	S	64	R	128	R	1	S	1	
2	S	1	S	>128	R	0.5	S	1	
2	S	1	S	>128	R	1	S	1	
2	S	1	S	1	S	1	S	0.5	
1	S	1	S	0.125	S	2	S	4	
1	S	0.5	S	0.06	S	1	S	1	
1	S	0.5	S	0.06	S	1	S	0.5	
8	S	16	R	8	R	0.5	S	16	
2	S	1	S	128	R	0.5	S	0.5	
2	S	1	S	64	R	1	S	0.5	
2	S	0.5	S	32	R	0.5	S	0.25	
>128	R	256	R	128	R	1	S	1	
8	S	1	S	>128	R	0.5	S	1	
1	S	0.5	S	128	R	0.5	S	1	
1	S	0.5	S	128	R	0.5	S	1	
1	S	0.5	S	64	R	1	S	0.5	
8	S	16	R	>128	R	0.5	S	1	

>128	R	256	R	64	R	0.5	S	1
>128	R	256	R	32	R	32	R	1
2	S	16	R	8	R	>128	R	1
1	S	0.5	S	0.5	S	0.5	S	0.25
64	R	0.5	S	0.5	S	>128	R	0.5
1	S	0.5	S	0.5	S	0.5	S	0.5
1	S	1	S	0.25	S	0.5	S	1
2	S	1	S	0.06	S	0.5	S	0.5
1	S	0.5	S	0.125	S	0.5	S	1
2	S	0.5	S	<0.03	S	1	S	0.5
2	S	0.5	S	1	S	0.5	S	1
1	S	0.25	S	0.06	S	2	S	1
0.5	S	0.5	S	0.06	S	1	S	1
1	S	0.5	S	<0.03	S	0.5	S	0.5
0.5	S	1	S	0.5	S	0.5	S	0.25
2	S	1	S	2	I	0.5	S	2
16	S	256	R	128	R	0.5	S	0.25
8	S	1	S	>128	R	0.5	S	0.5
1	S	16	R	>128	R	0.5	S	1
>128	R	256	R	64	R	0.5	S	1
>128	R	256	R	>128	R	0.5	S	1
1	S	0.25	S	64	R	32	R	1
2	S	1	S	>128	R	0.5	S	1
>128	R	256	R	128	R	0.5	S	1
2	S	16	R	128	R	0.5	S	1
2	S	128	R	1	S	0.5	S	0.5
1	S	16	R	0.5	S	0.5	S	0.5
>128	R	256	R	64	R	0.5	S	1
2	S	4	S	1	S	0.5	S	0.5
1	S	64	R	1	S	1	S	1
1	S	1	S	1	S	0.5	S	0.5
4	S	128	R	2	I	1	S	1
1	S	1	S	8	R	0.5	S	1
2	S	0.5	S	0.5	S	>128	R	0.5
2	S	8	I	64	R	0.5	S	2
2	S	0.5	S	0.06	S	0.5	S	1
4	S	1	S	64	R	0.5	S	0.5
4	S	128	R	>128	R	0.5	S	0.5
2	S	0.5	S	1	S	0.5	S	1
4	S	16	R	1	S	0.5	S	1
2	S	8	I	>128	R	0.5	S	1
2	S	0.5	S	2	I	0.5	S	0.5
2	S	0.5	S	2	I	0.5	S	1
2	S	64	R	32	R	0.5	S	1
2	S	0.5	S	128	R	0.5	S	1
4	S	0.5	S	0.125	S	128	R	1

2	S	0.5	S	0.06	S	0.5	S	1
2	S	0.5	S	<0.03	S	1	S	0.5
1	S	0.25	S	0.06	S	>128	R	1
1	S	0.5	S	<0.03	S	0.5	S	1
2	S	64	R	0.5	S	>128	R	0.5
2	S	64	R	32	R	0.5	S	1
2	S	0.5	S	128	R	0.5	S	1
1	S	0.5	S	<0.03	S	128	R	1
2	S	0.5	S	2	I	1	S	1
>128	R	256	R	128	R	1	S	1
>128	R	256	R	64	R	0.5	S	1
4	S	0.5	S	128	R	0.5	S	1
1	S	32	R	128	R	0.5	S	1
2	S	0.5	S	0.06	S	>128	R	1
4	S	16	R	16	R	1	S	1
4	S	128	R	32	R	1	S	1
2	S	0.5	S	0.06	S	1	S	0.5
8	S	0.5	S	0.25	S	1	S	0.5
2	S	1	S	128	R	0.5	S	0.5
1	S	2	S	1	S	1	S	1
4	S	256	R	8	R	0.5	S	0.25
32	I	2	S	128	R	0.5	S	1
1	S	0.25	S	0.125	S	16	R	1
2	S	0.5	S	0.06	S	0.5	S	1
2	S	0.25	S	0.125	S	>128	R	1
1	S	64	R	128	R	2	S	1
1	S	128	R	2	I	1	S	1
2	S	0.5	S	<0.03	S	0.5	S	0.5
1	S	32	R	1	S	0.5	S	1
>128	R	32	R	128	R	0.5	S	1
2	S	256	R	8	R	0.5	S	0.06
4	S	256	R	32	R	0.5	S	0.25
1	S	0.5	S	128	R	0.5	S	1
>128	R	256	R	128	R	0.5	S	1
>128	R	256	R	128	R	0.5	S	1
>128	R	256	R	128	R	0.5	S	1
2	S	64	R	64	R	1	S	0.25
8	S	0.5	S	>128	R	0.5	S	1
2	S	0.25	S	>128	R	0.5	S	1
>128	R	256	R	128	R	0.5	S	1
>128	R	256	R	128	R	0.5	S	1
4	S	0.5	S	128	R	0.5	S	1
4	S	2	S	0.5	S	0.5	S	0.25
1	S	1	S	128	R	0.5	S	1
>128	R	256	R	32	R	0.5	S	1
>128	R	256	R	128	R	0.5	S	0.5

>128	R	256	R	32	R	0.5	S	1
8	S	0.25	S	128	R	0.5	S	1
1	S	0.5	S	128	R	0.5	S	1
1	S	0.25	S	0.06	S	0.5	S	0.5
4	S	2	S	4	R	0.5	S	0.5
2	S	256	R	32	R	0.5	S	0.25
2	S	2	S	128	R	0.5	S	4
2	S	256	R	32	R	0.5	S	0.5
>128	R	256	R	32	R	1	S	1
1	S	0.5	S	128	R	0.5	S	1
1	S	0.5	S	128	R	0.5	S	1
1	S	0.5	S	1	S	0.5	S	0.5
>128	R	256	R	32	R	0.5	S	1
1	S	64	R	128	R	0.5	S	1
2	S	0.5	S	128	R	0.5	S	1
>128	R	0.5	S	>128	R	0.5	S	1
>128	R	256	R	16	R	0.25	S	0.5
2	S	8	I	1	S	0.5	S	1
>128	R	256	R	0.5	S	0.5	S	1
>128	R	16	R	>128	R	1	S	0.5
>128	R	256	R	>128	R	0.5	S	1
1	S	16	R	>128	R	4	R	8
4	S	0.5	S	128	R	0.5	S	1
>128	R	16	R	128	R	0.5	S	8
1	S	1	S	64	R	32	R	0.5
1	S	32	R	128	R	0.5	S	1
2	S	0.5	S	128	R	0.5	S	1
>128	R	256	R	32	R	0.5	S	1
4	S	1	S	<0.03	S	0.5	S	1
1	S	0.5	S	16	R	0.5	S	0.5
>128	R	256	R	128	R	0.5	S	0.25
1	S	16	R	16	R	0.5	S	1
>128	R	256	R	16	R	0.5	S	1
16	S	2	S	4	R	0.5	S	1
>128	R	128	R	128	R	0.5	S	1
1	S	0.5	S	128	R	0.5	S	1
8	S	1	S	128	R	0.5	S	1
>128	R	256	R	16	R	0.5	S	1
2	S	1	S	>128	R	0.5	S	1
2	S	16	R	>128	R	0.5	S	1
1	S	64	R	>128	R	0.5	S	1
>128	R	>256	R	32	R	0.5	S	1
>128	R	>256	R	128	R	0.5	S	1
1	S	16	R	64	R	0.5	S	1
1	S	0.25	S	2	I	0.5	S	0.5
>128	R	>256	R	128	R	0.5	S	0.5

1	S	16	R	128	R	0.5	S	1
1	S	0.25	S	128	R	0.25	S	1
2	S	16	R	>128	R	0.5	S	0.5
1	S	0.19	S	128	R	0.5	S	1
>128	R	16	R	32	R	0.5	S	1
2	S	0.25	S	128	R	0.5	S	1
1	S	0.38	S	128	R	0.5	S	1
>128	R	>256	R	128	R	0.5	S	1
1	S	>256	R	128	R	0.5	S	1
>128	R	>256	R	16	R	0.5	S	1
4	S	64	R	1	S	0.5	S	1
4	S	64	R	1	S	0.5	S	1
8	S	1	S	>128	R	0.5	S	1
1	S	0.75	S	0.25	S	0.5	S	1
2	S	0.75	S	128	R	0.5	S	0.5
>128	R	>256	R	128	R	0.5	S	0.5
1	S	1	S	32	R	0.5	S	0.5
2	S	0.5	S	>128	R	0.5	S	1
1	S	128	R	64	R	0.5	S	1
1	S	4	S	16	R	0.5	S	1
2	S	64	R	128	R	0.5	S	0.5
2	S	64	R	16	R	1	S	4
1	S	32	R	128	R	0.5	S	1
1	S	64	R	128	R	0.5	S	1
64	R	32	R	>128	R	0.5	S	1
4	S	1	S	2	I	0.5	S	1
2	S	1	S	128	R	0.5	S	1
2	S	1	S	0.125	S	>128	R	1
1	S	0.5	S	0.06	S	0.5	S	1
2	S	1	S	128	R	0.5	S	0.5
2	S	128	R	128	R	0.5	S	1
1	S	128	R	128	R	2	S	1
>128	R	256	R	128	R	0.5	S	1
1	S	32	R	128	R	0.5	S	1
>128	R	256	R	128	R	1	S	1
4	S	64	R	16	R	0.5	S	1
>128	R	0.5	S	128	R	1	S	1
2	S	0.5	S	128	R	0.5	S	1
2	S	0.5	S	>128	R	0.5	S	1
2	S	1	S	128	R	0.5	S	1
2	S	256	R	128	R	0.5	S	1
2	S	64	R	1	S	0.5	S	1
>128	R	256	R	64	R	0.5	S	1
8	S	16	R	32	R	>128	R	1
2	S	0.5	S	0.125	S	1	S	1
8	S	1	S	128	R	4	R	1

1	S	0.5	S	0.06	S	0.5	S	0.5
2	S	0.5	S	0.5	S	0.5	S	0.5
>128	R	256	R	64	R	0.5	S	1
2	S	1	S	1	S	0.5	S	1
2	S	0.5	S	128	R	0.5	S	1
4	S	8	I	128	R	0.5	S	1
2	S	0.5	S	0.06	S	0.5	S	0.5
>128	R	256	R	16	R	0.5	S	1
2	S	0.5	S	4	R	0.5	S	1
4	S	256	R	0.5	S	0.5	S	0.5
2	S	0.5	S	<0.03	S	0.5	S	1
1	S	0.5	S	<0.03	S	0.5	S	1
2	S	1	S	1	S	0.5	S	1
1	S	0.5	S	0.06	S	0.5	S	1
1	S	0.5	S	0.125	S	>128	R	1
1	S	0.5	S	0.06	S	0.5	S	1
4	S	2	S	128	R	1	S	1
16	S	1	S	>128	R	0.5	S	1
2	S	1	S	>128	R	0.5	S	0.5
1	S	0.5	S	128	R	0.5	S	1
2	S	64	R	0.125	S	0.5	S	1
16	S	2	S	32	R	>128	R	1
8	S	1	S	0.25	S	>128	R	0.5
>128	R	256	R	128	R	0.5	S	1
1	S	0.5	S	0.125	S	4	R	1
2	S	1	S	0.125	S	>128	R	0.5
2	S	1	S	128	R	0.5	S	0.5
4	S	1	S	128	R	0.5	S	0.5
>128	R	16	R	>128	R	0.5	S	0.5
1	S	1	S	1	S	0.5	S	1
1	S	0.5	S	0.25	S	>128	R	0.5
4	S	0.5	S	0.125	S	0.5	S	1
1	S	0.5	S	0.06	S	>128	R	0.5
2	S	1	S	0.06	S	>128	R	0.5
2	S	0.5	S	<0.03	S	0.5	S	0.5
1	S	0.5	S	0.06	S	1	S	1
1	S	16	R	8	R	0.5	S	0.5
>128	R	16	R	>128	R	0.5	S	0.5
>128	R	16	R	32	R	>128	R	4
>128	R	16	R	>128	R	0.5	S	2
1	S	0.25	S	<0.03	S	1	S	0.5
1	S	1	S	16	R	0.5	S	0.5
1	S	8	I	32	R	0.5	S	0.5
2	S	128	R	0.5	S	0.5	S	0.25
2	S	128	R	4	R	0.5	S	0.5
1	S	0.5	S	0.06	S	0.5	S	0.5

32	I	16	R	0.5	S	0.5	S	1
1	S	0.5	S	16	R	0.5	S	0.5
2	S	0.5	S	0.06	S	0.5	S	0.25
1	S	16	R	0.06	S	0.5	S	1
1	S	1	S	<0.03	S	1	S	1
1	S	0.5	S	128	R	0.5	S	1
16	S	128	R	1	S	0.5	S	1
16	S	256	R	32	R	0.5	S	0.5
1	S	0.25	S	0.125	S	0.5	S	1
1	S	256	R	8	R	0.5	S	1
1	S	0.5	S	128	R	0.5	S	1
16	S	16	R	64	R	0.5	S	1
1	S	0.5	S	64	R	0.5	S	1
2	S	0.5	S	128	R	0.5	S	1
>128	R	128	R	>128	R	0.5	S	8
1	S	0.25	S	0.06	S	0.5	S	1
1	S	0.25	S	0.125	S	0.5	S	1
16	S	64	R	>128	R	0.5	S	1
4	S	64	R	128	R	0.5	S	0.25
1	S	16	R	0.06	S	0.5	S	1
2	S	64	R	8	R	0.5	S	1
2	S	1	S	0.25	S	2	S	1
2	S	16	R	8	R	0.5	S	0.5
2	S	128	R	128	R	0.5	S	0.5
2	S	1	S	32	R	0.5	S	0.5
>128	R	16	R	128	R	0.5	S	0.5
8	S	256	R	64	R	0.5	S	1
>128	R	256	R	128	R	0.5	S	1
4	S	1	S	128	R	0.5	S	0.25
1	S	128	R	128	R	0.5	S	1
1	S	16	R	128	R	0.5	S	0.5
1	S	32	R	0.125	S	0.5	S	0.125
2	S	32	R	0.06	S	0.5	S	0.5
1	S	0.5	S	0.125	S	0.5	S	1
1	S	0.5	S	64	R	0.5	S	1
1	S	16	R	2	I	0.5	S	1
4	S	16	R	2	I	>128	R	1
>128	R	16	R	>128	R	0.5	S	1
1	S	64	R	1	S	0.5	S	1
2	S	0.5	S	0.06	S	0.5	S	0.5
1	S	128	R	0.5	S	0.5	S	0.5
2	S	0.5	S	0.06	S	0.25	S	1
>128	R	256	R	128	R	0.5	S	1
2	S	1	S	16	R	4	R	4
2	S	128	R	64	R	0.5	S	0.5
2	S	128	R	>128	R	0.5	S	1

1	S	128	R	32	R	1	S	0.5
4	S	1	S	32	R	0.5	S	0.25
2	S	16	R	8	R	32	R	2
>128	R	256	R	>128	R	0.5	S	1
>128	R	16	R	128	R	0.5	S	1
>128	R	256	R	64	R	0.5	S	1
1	S	16	R	0.06	S	0.5	S	1
4	S	0.25	S	128	R	0.5	S	1
1	S	0.5	S	128	R	0.5	S	1
2	S	16	R	8	R	0.5	S	1
1	S	0.5	S	128	R	0.5	S	1
2	S	0.25	S	32	R	0.5	S	0.5
2	S	0.5	S	128	R	32	R	1
2	S	0.5	S	32	R	8	R	1
1	S	0.5	S	>128	R	0.5	S	1
1	S	16	R	>128	R	0.5	S	1
2	S	0.5	S	0.125	S	0.5	S	1
2	S	0.25	S	128	R	0.5	S	1
>128	R	16	R	128	R	64	R	1
1	S	0.5	S	64	R	0.5	S	4
1	S	0.5	S	128	R	0.5	S	1
1	S	0.5	S	>128	R	0.5	S	1
2	S	32	R	0.25	S	0.5	S	1
>128	R	1	S	0.125	S	0.5	S	1
>128	R	0.5	S	>128	R	0.5	S	1
2	S	0.5	S	64	R	0.5	S	4
1	S	256	R	128	R	0.5	S	1
1	S	256	R	128	R	0.5	S	1
2	S	1	S	64	R	0.5	S	1
>128	R	1	S	128	R	0.5	S	1
4	S	0.5	S	32	R	0.5	S	0.25
1	S	256	R	2	I	0.5	S	1
1	S	0.5	S	>128	R	0.5	S	1
>128	R	256	R	32	R	0.5	S	1
>128	R	16	R	64	R	0.25	S	1
1	S	0.5	S	128	R	0.5	S	1
1	S	1	S	>128	R	0.5	S	8
1	S	0.5	S	0.06	S	0.5	S	1
1	S	2	S	16	R	0.5	S	1
1	S	0.5	S	<0.03	R	0.5	S	1
1	S	256	R	16	R	0.5	S	4
1	S	16	R	128	R	0.5	S	0.5
>128	R	16	R	>128	R	0.5	S	1
1	S	0.5	S	128	R	0.5	S	1
>128	R	256	R	>128	R	0.5	S	1
128	R	256	R	>128	R	0.25	S	0.25

1	S	2	S	128	R	0.5	S	4
1	S	0.5	S	128	R	0.5	S	1
2	S	2	S	128	R	0.5	S	4
2	S	1	S	128	R	0.25	S	0.25
1	S	0.5	S	128	R	0.5	S	1
2	S	0.5	S	0.06	S	0.25	S	0.25
1	S	1	S	>128	R	0.5	S	1
4	S	1	S	0.5	S	0.25	S	1
1	S	0.25	S	0.06	S	0.5	S	0.5
8	S	32	R	>128	R	0.5	S	0.5
>128	R	256	R	>128	R	0.5	S	1
2	S	128	R	1	S	0.5	S	0.5
16	S	256	R	64	R	0.5	S	1
2	S	256	R	2	I	0.5	S	0.5
>128	R	256	R	>128	R	0.5	S	0.5
1	S	0.25	S	128	R	0.5	S	1
1	S	0.5	S	0.06	S	1	S	0.5
1	S	2	S	8	R	0.5	S	1
1	S	16	R	128	R	0.5	S	0.5
1	S	8	I	>128	R	0.5	S	1
2	S	16	R	32	R	0.5	S	8
1	S	0.5	S	128	R	0.5	S	1
4	S	1	S	1	S	128	R	0.5
>128	R	16	R	>128	R	0.5	S	0.5
4	S	1	S	128	R	0.5	S	1
2	S	0.5	S	8	R	0.5	S	0.06
2	S	0.5	S	0.06	S	1	S	0.5
1	S	1	S	4	R	>128	R	1
8	S	128	R	>128	R	32	R	1
2	S	1	S	64	R	0.5	S	0.25
2	S	64	R	128	R	0.5	S	1
>128	R	64	R	128	R	0.5	S	1
1	S	8	I	>128	R	0.5	S	8
1	S	16	R	1	S	0.5	S	2
2	S	16	R	>128	R	0.5	S	1
>128	R	256	R	128	R	0.5	S	1
>128	R	256	R	128	R	0.5	S	1
1	S	0.5	S	>128	R	0.5	S	1
2	S	1	S	4	R	0.5	S	2
>128	R	256	R	128	R	0.5	S	1
4	S	16	R	16	R	1	S	0.5
>128	R	256	R	128	R	0.5	S	1
>128	R	256	R	128	R	0.5	S	1
1	S	1	S	4	R	0.5	S	8
1	S	0.5	S	<0.03	S	0.25	S	0.5
8	S	0.5	S	<0.03	S	0.5	S	0.5

2	S	1	S	0.06	S	16	R	0.5
1	S	1	S	0.06	S	128	R	0.5
2	S	256	R	1	S	0.5	S	0.5
8	S	2	S	>128	R	0.5	S	0.5
>128	R	256	R	128	R	0.5	S	1
32	I	16	R	128	R	0.5	S	0.5
1	S	1	S	1	S	1	S	2
2	S	1	S	1	S	0.5	S	1
2	S	1	S	>128	R	0.5	S	1
1	S	16	R	1	S	1	S	1
1	S	1	S	>128	R	64	R	16
2	S	1	S	0.125	S	0.5	S	4
2	S	1	S	16	R	0.5	S	8
1	S	1	S	0.06	S	0.5	S	0.25
2	S	256	R	<0.03	S	0.5	S	0.125
1	S	1	S	0.25	S	0.5	S	0.25
1	S	2	S	0.06	S	0.5	S	0.5
16	S	0.5	S	8	R	0.25	S	0.5
4	S	256	R	0.5	S	0.5	S	0.25
8	S	128	R	>128	R	0.5	S	0.5
2	S	16	R	4	R	0.5	S	0.25
1	S	1	S	8	R	0.5	S	1
2	S	1	S	1	S	0.5	S	0.5
1	S	16	R	>128	R	0.5	S	0.5
>128	R	128	R	128	R	0.5	S	1
1	S	1	S	>128	R	0.5	S	0.5
2	S	1	S	16	R	0.25	S	0.25
1	S	0.5	S	0.06	S	0.5	S	0.5
1	S	1	S	0.25	S	0.5	S	4
4	S	256	R	16	R	0.25	S	0.5
1	S	64	R	128	R	0.5	S	1
>128	R	128	R	128	R	0.5	S	1
1	S	32	R	>128	R	0.25	S	1
2	S	128	R	32	R	0.25	S	1
8	S	16	R	>128	R	0.25	S	1
>128	R	16	R	64	R	0.25	S	0.5
1	S	0.5	S	128	R	1	S	1
1	S	1	S	128	R	64	R	1
1	S	32	R	128	R	0.5	S	1
2	S	64	R	1	S	0.25	S	0.5
>128	R	16	R	>128	R	0.25	S	1
2	S	16	R	0.5	S	0.25	S	1
1	S	128	R	<0.03	S	0.25	S	0.125
2	S	1	S	8	R	0.25	S	1
2	S	1	S	<0.03	S	0.5	S	0.5
2	S	64	R	0.125	S	0.5	S	1

1	S	0.5	S	<0.03	S	0.5	S	0.5
1	S	0.5	S	0.06	S	0.5	S	1
1	S	0.5	S	0.125	S	0.5	S	1
0.5	S	0.5	S	0.25	S	0.5	S	1
2	S	2	S	0.125	S	>128	R	1
>128	R	16	R	128	R	0.5	S	1
2	S	0.5	S	>128	R	0.25	S	0.5
2	S	0.5	S	32	R	0.5	S	0.5
16	S	4	S	>128	R	0.5	S	1
>128	R	16	R	128	R	0.5	S	0.5
8	S	128	R	1	S	0.5	S	1
1	S	256	R	>128	R	0.5	S	0.5
2	S	8	I	128	R	0.5	S	1
2	S	16	R	64	R	0.5	S	16
>128	R	0.5	S	128	R	0.5	S	1
>128	R	64	R	128	R	0.5	S	1
2	S	1	S	64	R	0.5	S	0.5
2	S	256	R	64	R	0.5	S	0.5
1	S	256	R	1	S	0.5	S	1
>128	R	128	R	128	R	0.5	S	1
4	S	0.5	S	0.125	S	0.5	S	1
2	S	1	S	16	R	0.5	S	8
1	S	0.25	S	0.06	S	1	S	1
1	S	0.25	S	64	R	0.5	S	0.5
2	S	64	R	128	R	0.5	S	0.25
2	S	4	S	>128	R	0.5	S	0.25
2	S	0.5	S	>128	R	0.5	S	1
2	S	16	R	128	R	0.5	S	1
>128	R	256	R	128	R	0.5	S	1
1	S	0.25	S	128	R	0.5	S	1
16	S	1	S	8	R	0.5	S	1
1	S	1	S	0.5	S	1	S	1
>128	R	256	R	>128	R	0.5	S	1
1	S	16	R	64	R	0.5	S	1
8	S	2	S	0.5	S	>128	R	1
4	S	2	S	0.25	S	>128	R	1
>128	R	256	R	8	R	0.5	S	1
2	S	0.5	S	<0.03	S	0.5	S	1
2	S	0.5	S	0.06	S	0.25	S	1
2	S	0.5	S	0.06	S	>128	R	1
2	S	1	S	1	S	0.25	S	1
2	S	0.5	S	64	R	0.25	S	0.25
4	S	8	I	2	I	0.5	S	0.5
4	S	16	R	64	R	0.5	S	4
>128	R	256	R	128	R	0.5	S	1
1	S	0.25	S	128	R	0.5	S	1

>128	R	256	R	128	R	0.5	S	1
2	S	16	R	>128	R	1	S	1
>128	R	256	R	128	R	0.5	S	1
>128	R	256	R	128	R	0.5	S	1
4	S	1	S	>128	R	0.5	S	2
2	S	1	S	16	R	0.5	S	8
1	S	0.5	S	0.06	S	0.5	S	1
2	S	0.25	S	0.06	S	0.5	S	0.5
2	S	1	S	128	R	0.5	S	0.25
1	S	0.25	S	0.125	S	8	R	1
8	S	128	R	>128	R	0.5	S	1
2	S	16	R	2	I	0.5	S	0.25
4	S	0.5	S	128	R	0.5	S	0.5
128	R	128	R	64	R	0.5	S	1
16	S	128	R	>128	R	0.5	S	1
1	S	0.25	S	32	R	0.5	S	1
32	I	16	R	>128	R	0.5	S	1
>128	R	256	R	128	R	0.5	S	1
>128	R	256	R	128	R	0.5	S	1
>128	R	256	R	>128	R	0.5	S	1
2	S	1	S	>128	R	0.5	S	1
2	S	1	S	4	R	4	R	1
2	S	16	R	1	S	1	S	2
2	S	0.5	S	1	S	1	S	0.5
1	S	1	S	0.125	S	0.5	S	8
4	S	1	S	64	R	0.25	S	0.5
2	S	64	R	>128	R	0.5	S	1
2	S	16	R	>128	R	0.5	S	1
2	S	64	R	>128	R	0.5	S	1
2	S	128	R	1	S	0.5	S	1
2	S	128	R	1	S	0.5	S	1
1	S	1	S	2	I	0.5	S	1

TGC_C	ESBL	AmpC	ESBL_An	E_A_grou	Carbape	Hosp_g	ERT_g	IMP_g
S	N	N		0		0	R	R
S	N	N		0		0	R	R
S	N	N		0	IMP-8	0	R	R
S	N	N		0		0	R	S
S	N	N		0		0	S	R
S	N	N		0		0	R	S
R	SHV	N		1		0	R	R
S	N	N		0		0	R	R
R	N	N		0		0	R	R
S	N	N		0		0	S	R
S	N	N		0		0	S	R
S	N	N		0		0	R	R
S	N	N		0		0	R	S
S	N	N		0		0	R	S
S	N	N		0		0	R	S
R	N	N		0		0	R	S
S	N	MIR		2		0	R	R
R	SHV	ACT	SHV,ACT	3	IMP-8	0	R	R
S	N	N		0		0	R	S
S	N	N		0		0	R	R
S	N	N		0		0	R	R
S	N	N		0		0	S	R
S	N	N		0		0	R	R
R	N	N		0		0	R	S
S	N	N		0		0	R	S
S	N	N		0		0	R	S
S	SHV	N		1	IMP-8	0	R	R
S	N	N		0		0	R	S
S	N	DHA		2		0	R	R
S	CTX-M	N		1		0	R	S
S	N	CMY		2		0	R	S
S	CTX-M	CMY	CTX-M,C	3		0	R	S
S	CTX-M	CMY	CTX-M,C	3		0	R	S
S	N	CMY		2		0	R	R
S	N	CMY		2		0	R	R
S	N	CMY		2		0	R	R
S	N	CMY		2		0	R	R
S	N	CMY		2		0	R	R
S	N	N		0		0	R	S
S	N	CMY		2		0	R	R
S	N	CMY		2		0	R	R
S	CTX-M	N		1		0	R	R
S	CTX-M	DHA	CTX-M,D	3		0	S	R
R	SHV	DHA	SHV,DH	3		0	R	S
S	CTX-M	DHA	CTX-M,D	3		0	R	R

S	CTX-M	DHA	CTX-M,D	3		0	R	R
S	CTX-M	N		1		0	R	S
S	N	DHA		2		0	R	S
S	N	CMY		2		0	R	R
S	N	DHA		2		0	R	R
S	N	N		0		0	R	R
S	CTX-M	DHA	CTX-M,D	3		0	S	R
S	CTX-M	DHA	CTX-M,D	3		0	R	R
S	N	DHA		2		0	R	R
S	N	DHA		2		0	S	R
S	N	CMY		2		0	R	R
R	N	DHA		2		0	R	R
S	CTX-M	DHA	CTX-M,D	3		0	R	S
S	CTX-M	DHA	CTX-M,D	3		0	R	R
S	CTX-M	CMY	CTX-M,C	3	KPC-2	0	R	R
R	N	N		0		0	R	R
S	N	N		0		0	R	R
S	N	N		0		0	R	S
S	N	N		0		0	R	S
S	N	N		0		0	R	S
S	N	MIR		2		0	R	S
S	N	N		0		0	R	S
S	N	N		0		0	R	R
S	N	MIR		2		0	R	S
S	N	N		0		0	R	S
R	N	N		0		0	R	S
R	SHV	N		1		0	R	S
S	N	N		0		0	R	S
S	N	N		0		0	R	S
R	N	N		0		0	R	S
S	CTX-M	DHA	CTX-M,D	3		0	R	S
S	CTX-M	N		1		0	R	S
S	CTX-M	N		1		0	R	S
S	N	DHA		2		0	R	S
S	N	DHA		2		0	R	R
S	CTX-M 8	N		1		0	R	S
S	CTX-M	N		1		0	R	S
S	CTX-M	N		1		0	R	S
S	CTX-M	DHA	CTX-M,D	3		0	R	S
S	SHV	DHA	SHV,DHA	3		0	R	S
S	CTX-M	DHA	CTX-M,D	3		0	R	S
S	N	N		0		0	R	S
S	CTX-M	DHA	CTX-M,D	3		0	R	R
S	CTX-M	DHA	CTX-M,D	3		0	R	S
S	CTX-M	DHA	CTX-M,D	3		0	R	R
R	N	DHA		2		0	R	R

S	N	DHA		2		0	R	S
R	N	N		0		0	R	S
S	N	DHA		2		0	R	S
S	N	CMY		2		0	R	S
S	N	N		0		0	R	S
S	N	N		0		0	R	S
S	SHV	N		1		0	R	S
S	N	N		0		0	R	R
S	CTX-M	CMY	CTX-M,C	3		0	R	S
S	N	CMY		2		0	R	S
S	CTX-M &	N		1	NDM-1	0	R	R
S	CTX-M	DHA	CTX-M,D	3		0	R	S
S	SHV	N		1		0	R	S
S	CTX-M	DHA	CTX-M,D	3		0	R	S
S	N	DHA		2		0	R	R
S	CTX-M	DHA	CTX-M,D	3		0	R	S
S	N	DHA		2		0	R	S
S	N	DHA		2		0	R	S
R	N	DHA		2		0	R	S
S	CTX-M &	DHA	CTX-M&	3		0	R	R
S	CTX-M	DHA	CTX-M,D	3		0	R	S
S	CTX-M	DHA	CTX-M,D	3		0	R	R
S	CTX-M	DHA	CTX-M,D	3		0	R	R
R	SHV	DHA	SHV,DH	3		0	R	S
S	N	CMY		2	IMP-8	0	R	R
S	CTX-M	DHA	CTX-M,D	3		0	R	R
S	N	DHA		2		0	R	S
R	CTX-M	N		1		0	R	R
S	N	MIR		2		0	R	S
R	N	N		0		0	R	S
S	N	N		0		0	R	S
S	CTX-M &	DHA	CTX-M&	3		0	R	S
S	N	DHA		2		0	R	S
S	SHV	DHA	SHV,DH	3		0	R	R
S	CTX-M	DHA	CTX-M,D	3		0	R	S
S	N	N		0		0	R	S
S	N	CMY		2		0	R	S
S	SHV	DHA	SHV,DH	3		0	R	S
S	CTX-M &	N		1		0	R	R
S	N	DHA		2		0	R	S
S	CTX-M	N		1		0	R	S
R	N	N		0		0	R	S
S	N	N		0		0	R	S
S	SHV	N		1		0	R	S
S	CTX-M	DHA	CTX-M,D	3		0	R	R
S	N	ACT		2		0	R	R

S	SHV	DHA	SHV,DH/	3	0	R	S
S	N	CMY		2	0	R	S
S	CTX-M	N		1	0	R	S
S	N	DHA		2	0	R	S
S	CTX-M	DHA	CTX-M,[	3	0	R	S
S	N	N		0	0	R	S
R	N	DHA		2	0	R	S
S	N	DHA		2	0	R	S
S	N	N		0	0	R	S
S	N	N		0	0	R	S
S	N	N		0	0	R	R
S	N	DHA		2	0	R	S
S	N	DHA, CM		2	0	R	S
S	N	MIR		2	0	R	S
S	N	DHA		2	0	R	S
S	N	DHA		2	0	R	S
S	N	N		0	0	R	S
S	CTX-M	N		1	0	R	S
S	SHV	DHA	SHV,DH/	3	0	R	R
S	SHV	N		1	0	R	S
S	N	DHA		2	0	R	S
S	N	N		0	0	R	R
S	CTX-M	N		1	0	R	S
S	N	N		0	0	R	S
S	N	DHA		2	0	R	S
S	N	DHA		2	0	R	R
S	N	N		0	0	R	S
S	N	DHA		2	0	R	S
R	N	N		0	0	R	S
R	N	N		0	0	R	S
S	N	N		0	0	R	R
S	N	CMY		2	0	R	S
S	N	DHA		2	0	R	S
S	N	DHA		2	0	R	R
S	N	DHA		2	0	R	S
S	N	ACT		2	0	R	S
S	N	CMY		2	0	R	S
S	CTX-M	DHA	CTX-M,[	3	0	R	S
S	N	DHA		2	0	R	R
R	SHV	N		1	0	R	S
S	SHV	DHA	SHV,DH/	3	0	R	S
S	N	N		0	0	R	S
S	SHV	DHA	SHV,DH/	3	0	R	R
R	N	N		0	0	R	S
S	N	N		0	0	R	S
S	N	DHA		2	0	R	S

S	CTX-M	CMY	CTX-M,C	3		0	R	S
S	N	DHA		2		0	R	S
S	N	DHA		2		0	R	R
S	SHV	DHA	SHV,DHA	3		0	R	S
S	N	MIR		2		0	R	S
S	SHV	ACT	SHV,ACT	3	IMP-8	0	R	R
S	SHV	DHA	SHV,DHA	3		0	R	S
S	N	N		0		0	R	S
S	N	DHA		2		0	R	S
S	CTX-M	CMY	CTX-M,C	3		0	R	S
S	N	DHA		2		0	R	S
S	N	N		0		0	R	R
S	N	MIR		2		0	R	S
S	N	N		0		0	R	S
S	CTX-M	N		1		0	R	S
S	N	CMY		2		0	R	S
S	N	DHA		2		0	R	S
S	N	DHA		2		0	R	S
S	N	DHA		2		0	S	R
S	SHV	N		1		0	R	R
S	SHV	DHA	SHV,DHA	3		0	R	S
S	N	ACT		2		0	R	S
R	CTX-M	DHA	CTX-M,D	3		0	R	S
R	N	DHA		2		0	R	S
S	CTX-M	CMY	CTX-M,C	3		0	R	S
R	N	MIR		2		0	R	S
S	SHV	DHA	SHV,DHA	3		0	R	S
S	N	DHA		2		0	R	S
S	N	N		0	IMP-8	0	R	R
S	N	N		0		0	R	S
R	N	N		0		0	R	S
R	N	N		0		0	R	R
S	N	CMY		2		0	R	S
R	N	N		0		0	R	S
S	CTX-M	CMY	CTX-M,C	3		0	R	S
R	CTX-M	N		1		0	R	R
S	N	DHA		2		0	R	S
S	N	N		0		0	R	S
R	N	CMY		2		0	R	S
R	SHV	N		1		0	R	S
S	CTX-M	DHA	CTX-M,D	3		0	R	S
R	CTX-M	DHA	CTX-M,D	3		0	R	S
S	N	DHA		2		0	R	S
S	CTX-M	DHA	CTX-M,D	3		0	R	S
S	N	N		0	IMP-8	0	R	S
S	N	N		0		0	R	S

S	CTX-M	N		1		0	R	S
R	N	N		0		0	R	S
S	SHV	CMY	SHV,CMY	3		0	R	S
S	CTX-M &	N		1		0	R	S
S	N	CMY		2		0	R	S
S	CTX-M	N		1		0	R	S
S	N	DHA		2		0	R	R
S	CTX-M	DHA	CTX-M,D	3		0	R	S
S	N	DHA		2		0	R	S
R	N	N		0		0	R	S
S	N	N		0		0	R	S
S	N	N		0		0	R	S
R	N	DHA		2		0	R	S
S	N	N		0		0	R	S
S	N	MIR		2		0	R	S
S	CTX-M	DHA	CTX-M,D	3		0	R	S
S	N	CMY		2		0	R	R
S	N	N		0		0	R	S
S	CTX-M	DHA	CTX-M,D	3		0	R	S
S	N	N		0		0	R	S
S	SHV	N		1		0	R	R
S	N	CMY		2		0	R	S
S	CTX-M	CMY	CTX-M,C	3		0	R	R
S	N	DHA		2		0	R	S
S	CTX-M	DHA	CTX-M,D	3		0	R	R
S	CTX-M &	DHA	CTX-M&	3		0	R	S
S	CTX-M	DHA	CTX-M,D	3		0	R	S
S	CTX-M	DHA	CTX-M,D	3		0	R	S
S	N	N		0		0	R	S
S	N	N		0		0	R	S
R	N	N		0		0	R	R
S	N	DHA		2		0	R	R
S	N	N		0		0	R	S
S	N	N		0		0	R	S
S	N	ACT		2		0	R	R
S	N	N		0		0	R	R
S	N	N		0		0	R	S
S	N	N		0		0	R	S
S	N	N		0		0	R	R
S	N	N		0		0	R	S
S	N	CMY		2		0	R	S
S	CTX-M	CMY	CTX-M,C	3		0	R	S
S	N	CMY		2		0	R	S
S	N	N		0	KPC-2	0	R	R
S	CTX-M &	N		1		0	R	S
S	CTX-M	DHA	CTX-M,D	3		0	R	S

S	CTX-M	DHA	CTX-M,D	3		0	R	S
S	CTX-M	DHA	CTX-M,D	3		0	R	S
S	SHV	DHA	SHV,DH	3		0	R	S
S	N	MIR		2		0	R	S
S	CTX-M &	DHA	CTX-M&	3		0	R	S
S	CTX-M	DHA	CTX-M,D	3		0	R	S
S	N	DHA		2		0	R	S
S	CTX-M	DHA	CTX-M,D	3		0	R	S
S	N	N		0		0	R	S
S	N	N		0		0	R	S
R	N	ACT		2		0	R	S
S	N	N		0		0	R	S
S	N	DHA		2		0	S	R
S	N	ACT		2		0	R	S
S	N	N		0		0	R	S
S	CTX-M	DHA	CTX-M,D	3		0	R	S
S	N	N		0		0	R	S
S	CTX-M	N		1		0	R	S
S	N	MIR		2		0	R	S
S	N	MIR		2		0	R	S
S	SHV	DHA	SHV,DH	3		0	R	R
S	N	N		0		0	R	S
S	N	N		0		0	R	S
S	CTX-M	N		1		0	R	S
S	N	N		0		0	R	R
S	N	N		0		0	R	R
S	N	N		0		0	R	S
R	N	N		0		0	R	R
R	CTX-M	N		1		0	R	R
S	CTX-M &	N		1		0	R	S
S	N	N		0		0	R	R
S	N	N		0		0	R	R
S	CTX-M &	DHA	CTX-M&	3		0	R	R
S	CTX-M	N		1		0	R	S
S	N	DHA		2		0	R	S
S	N	N		0	IMP-8	0	R	R
S	N	DHA		2		0	R	R
S	CTX-M	N		1		0	R	R
S	CTX-M	DHA	CTX-M,D	3		0	R	S
S	N	CMY		2		0	R	S
S	N	N		0		0	R	S
S	CTX-M	DHA	CTX-M,D	3		0	R	R
R	N	N		0		0	R	S
S	N	CMY		2		0	R	S
S	N	N		0		0	R	S
S	N	N		0		0	R	S

S	N	N		0		0	R	S
S	N	MIR		2		0	R	S
S	N	MIR		2		0	R	R
S	N	N		0		0	R	S
R	SHV	N		1		0	R	S
S	CTX-M	N		1		0	R	S
R	N	N		0		0	R	S
S	N	MIR		2		0	R	S
S	SHV	N		1		0	R	S
S	N	N		0	IMP-8	0	R	R
S	N	N		0		0	R	S
S	N	N		0		0	R	S
R	SHV	N		1	IMP-8	0	R	S
S	CTX-M 8	N		1		0	R	S
S	N	ACT		2		0	R	S
S	N	CMY		2		0	R	S
S	CTX-M	N		1		0	R	S
S	N	DHA		2		0	R	S
S	CTX-M	DHA	CTX-M,D	3		0	R	S
R	N	N		0		0	R	S
S	CTX-M	DHA	CTX-M,D	3		0	R	R
S	CTX-M	DHA	CTX-M,D	3		0	R	R
S	N	DHA		2		0	R	R
S	CTX-M	CMY	CTX-M,C	3		0	R	S
R	N	N		0		0	R	R
R	N	N		0		0	R	R
R	SHV	N		1	VIM-1	0	R	R
S	N	CMY		2		0	R	S
S	SHV	N		1	KPC-2	0	R	R
S	N	DHA		2		0	S	R
S	CTX-M	DHA	CTX-M,D	3		0	R	S
S	N	DHA		2		0	R	S
S	SHV	N		1		0	R	S
R	CTX-M	DHA	CTX-M,D	3		0	R	R
S	N	CMY		2		0	R	S
S	N	N		0		0	R	S
S	N	N		0		0	R	R
S	N	CMY		2		0	R	S
S	N	N		0		0	R	R
S	N	MIR		2		0	R	R
S	N	DHA		2		0	R	S
S	SHV	N		1		0	R	R
S	N	DHA		2		0	R	R
S	CTX-M 8	N		1	KPC-2	0	R	R
S	CTX-M 8	N		1	KPC-2	0	R	R
S	CTX-M 8	N		1	KPC-2	0	R	R

S	CTX-M	DHA	CTX-M,D	3		0	R	S
S	N	N		0		0	R	S
S	N	N		0		0	R	S
S	N	N		0		0	R	S
S	CTX-M &	N		1	KPC-2	0	R	R
S	CTX-M &	N		1	KPC-2	0	R	R
S	N	DHA		2		0	R	R
S	CTX-M	DHA	CTX-M,D	3		0	R	R
S	CTX-M	DHA	CTX-M,D	3		0	R	S
S	N	DHA		2		0	R	S
S	N	CMY		2		0	R	S
S	N	CMY		2		0	R	R
S	CTX-M	DHA	CTX-M,D	3		0	R	R
S	CTX-M	DHA	CTX-M,D	3		0	R	R
S	N	MIR		2		0	R	S
S	N	N		0		0	R	S
S	CTX-M	DHA	CTX-M,D	3		0	R	S
S	CTX-M	DHA	CTX-M,D	3		0	R	S
S	CTX-M	DHA	CTX-M,D	3		0	R	S
S	CTX-M	DHA	CTX-M,D	3		0	R	S
S	N	DHA		2		0	R	R
S	N	DHA		2		0	R	S
S	SHV	DHA	SHV,DH	3		0	R	R
S	N	DHA		2		0	R	R
S	N	DHA		2		0	R	S
S	CTX-M	DHA	CTX-M,D	3		1	R	S
S	N	DHA		2		1	R	R
R	N	N		0	IMP-8	1	R	S
S	N	DHA, CM		2		1	R	S
S	N	CMY		2		1	R	S
S	N	N		0	IMP-8	1	R	S
S	CTX-M	DHA	CTX-M,D	3		1	R	S
S	N	N		0		1	R	S
S	N	CMY		2		1	R	S
S	CTX-M	DHA	CTX-M,D	3		1	R	S
S	N	DHA		2		1	R	R
S	N	CMY		2		1	R	S
S	CTX-M	DHA	CTX-M,D	3		1	R	S
S	N	CMY		2		1	R	S
S	N	CMY		2		1	R	S
S	CTX-M	DHA	CTX-M,D	3		1	R	R
S	CTX-M	DHA	CTX-M,D	3		1	R	R
S	N	CMY		2		1	R	S
S	CTX-M	DHA	CTX-M,D	3		1	R	R
S	N	CMY		2		1	R	R
S	CTX-M	DHA	CTX-M,D	3		1	R	S

S	N	N		0		1	R	S
S	CTX-M	DHA	CTX-M,D	3		1	R	S
S	CTX-M	DHA	CTX-M,D	3		1	R	R
S	N	DHA		2		1	R	S
S	N	CMY		2		1	R	S
S	N	DHA, CM		2		1	R	S
S	N	DHA, CM		2		1	R	R
S	CTX-M	DHA	CTX-M,D	3		1	R	R
S	N	DHA		2		1	R	R
S	SHV, OX	N		1	IMP-8	1	R	R
S	CTX-M	DHA	CTX-M,D	3		1	R	S
S	N	CMY		2		1	R	S
S	CTX-M	DHA	CTX-M,D	3		1	R	S
S	CTX-M	N		1		1	R	R
S	CTX-M &	DHA	CTX-M&	3		1	R	R
S	CTX-M	DHA	CTX-M,D	3		1	R	R
S	CTX-M	CMY	CTX-M,C	3		1	R	S
S	CTX-M	DHA	CTX-M,D	3		1	R	S
S	N	N		0		1	R	S
S	N	DHA		2		1	R	S
R	N	DHA, CM		2		1	R	R
S	N	CMY		2		1	R	S
S	CTX-M	DHA	CTX-M,D	3		1	R	S
S	CTX-M	DHA	CTX-M,D	3		1	R	S
S	N	DHA		2		1	R	R
S	N	DHA		2		1	R	S
S	CTX-M	DHA	CTX-M,D	3		1	R	R
S	CTX-M	DHA	CTX-M,D	3		1	R	S
S	CTX-M	DHA	CTX-M,D	3		1	R	S
S	CTX-M	DHA	CTX-M,D	3		1	R	R
S	CTX-M	DHA	CTX-M,D	3		1	R	S
S	SHV	DHA	SHV,DHA	3		1	R	S
S	CTX-M	DHA	CTX-M,D	3		1	R	R
S	CTX-M	CMY	CTX-M,C	3		1	R	S
S	N	N		0		1	R	S
S	CTX-M	DHA	CTX-M,D	3		1	R	S
S	CTX-M	DHA	CTX-M,D	3		1	R	S
S	N	MIR		2		1	R	S
R	SHV	N		1		1	R	S
R	SHV	N		1		1	R	R
S	CTX-M	DHA	CTX-M,D	3		1	R	S
S	N	N		0		1	R	R
S	N	N		0		1	R	S
S	CTX-M	N		1		1	R	R
S	N	N		0		1	R	R
S	N	DHA		2		1	R	S

S	CTX-M	DHA	CTX-M,D	3		1	R	S
R	N	N		0		0	R	S
S	CTX-M	DHA	CTX-M,D	3		0	R	S
S	CTX-M	DHA	CTX-M,D	3		0	R	S
S	N	CMY		2		0	R	R
S	N	N		0		0	R	S
S	N	N		0		0	R	R
R	CTX-M	DHA	CTX-M,D	3		0	R	S
R	CTX-M	N		1		0	R	S
S	CTX-M	DHA	CTX-M,D	3		0	R	R
S	N	N		0		0	R	S
S	CTX-M	DHA	CTX-M,D	3		0	R	S
S	SHV	N		1	KPC-2	0	R	R
S	CTX-M	CMY	CTX-M,C	3		0	R	S
S	N	N		0		0	R	S
S	N	ACT		2		0	R	S
S	N	N		0		0	R	S
R	CTX-M	DHA	CTX-M,D	3		0	R	R
R	N	N		0		0	R	S
S	CTX-M	CMY	CTX-M,C	3		0	R	R
S	CTX-M	DHA	CTX-M,D	3		0	R	S
S	CTX-M	DHA	CTX-M,D	3		0	R	R
R	SHV	N		1		0	R	S
S	N	DHA, MI		2		0	R	R
S	N	N		0	IMP-8	0	R	R
S	N	DHA		2		0	R	R
R	N	DHA		2		0	R	S
S	N	N		0		0	R	R
S	CTX-M	DHA	CTX-M,D	3		0	R	S
S	N	N		0		0	S	R
S	CTX-M	CMY	CTX-M,C	3		0	R	S
S	CTX-M &	N		1	KPC-2	0	R	R
S	N	CMY		2		0	R	R
S	CTX-M	DHA	CTX-M,D	3		0	R	S
S	CTX-M	DHA	CTX-M,D	3		0	R	R
S	CTX-M &	N		1		0	R	S
S	N	N		0		0	R	S
S	CTX-M	N		1		0	R	R
S	N	CMY		2		0	R	S
S	N	DHA		2		0	R	S
R	CTX-M	N		1		0	R	S
S	N	N		0		0	R	S
S	SHV	N		1		0	R	S
S	SHV	N		1	IMP-8	0	R	R
S	N	CMY		2		0	R	R
S	CTX-M	DHA	CTX-M,D	3		1	R	S

S	N	N		0	1	R	S
S	N	DHA		2	1	R	S
S	N	DHA		2	1	R	S
S	CTX-M	DHA	CTX-M,D	3	1	R	R
S	N	DHA		2	1	R	S
S	N	DHA, CM		2	1	R	S
S	N	DHA, CM		2	1	R	S
S	N	DHA		2	1	R	S
S	N	CMY		2	1	R	S
S	N	DHA		2	1	R	S
S	CTX-M	DHA	CTX-M,D	3	1	R	R
S	N	N		0	0	R	R
S	N	CMY		2	0	R	S
S	CTX-M	N		1	0	R	R
S	N	DHA		2	0	R	R
S	CTX-M	N		1	0	R	S
S	CTX-M	N		1	0	R	R
S	SHV	ACT	SHV,ACT	3	0	R	R
S	N	N		0	0	R	S
S	N	N		0	0	R	S
S	SHV	N		1	0	R	R
S	N	N		0	0	R	R
S	CTX-M	CMY	CTX-M,C	3	0	R	R
S	CTX-M	CMY	CTX-M,C	3	0	R	R
S	SHV	N		1	0	R	S
S	N	DHA		2	0	R	S
S	N	DHA		2	0	R	R
S	CTX-M	DHA	CTX-M,D	3	0	R	S
S	CTX-M	DHA	CTX-M,D	3	0	R	S
S	CTX-M	DHA	CTX-M,D	3	0	R	S
S	CTX-M	DHA	CTX-M,D	3	0	R	S
S	CTX-M	DHA	CTX-M,D	3	0	R	S
S	CTX-M	DHA	CTX-M,D	3	0	R	S
S	CTX-M	DHA	CTX-M,D	3	0	R	S
S	CTX-M	N		1	0	R	S
S	N	DHA		2	0	R	R
S	N	CMY		2	0	R	S
S	SHV	N		1	0	R	S
S	N	DHA		2	0	R	R
R	N	N		0	0	R	S
S	CTX-M	DHA	CTX-M,D	3	0	R	S
S	CTX-M	DHA	CTX-M,D	3	0	R	S
S	N	CMY		2	0	R	S
S	CTX-M	N		1	0	R	S
R	SHV	N		1	0	R	S
S	N	N		0	0	R	S

S	CTX-M	N		1		0	R	R
S	CTX-M	CMY	CTX-M,C	3		0	R	S
S	CTX-M	DHA	CTX-M,D	3		0	R	S
S	N	MIR		2		0	R	R
S	N	DHA		2		0	S	R
S	CTX-M	DHA	CTX-M,D	3		0	R	S
S	CTX-M	DHA	CTX-M,D	3		0	R	R
S	CTX-M	N		1		0	R	R
S	N	N		0	IMP-8	0	S	R
S	N	DHA		2		0	R	S
S	CTX-M	DHA	CTX-M,D	3		0	R	R
R	N	N		0		0	R	S
S	N	CMY		2		0	R	R
S	CTX-M	DHA	CTX-M,D	3		0	R	S
S	N	DHA		2		0	R	R
S	N	N		0		0	R	S
S	CTX-M	DHA	CTX-M,D	3		0	R	R
S	CTX-M	DHA	CTX-M,D	3		0	R	R
S	CTX-M	DHA	CTX-M,D	3		0	R	S
S	N	N		0		0	R	S
S	CTX-M	DHA	CTX-M,D	3		0	R	R
S	SHV	DHA	SHV,DH/	3		0	R	S
S	N	CMY		2		0	R	S
S	N	N		0		0	R	R
S	N	N		0		0	R	R
S	N	MIR		2		0	R	S
S	N	N		0		0	R	S
R	N	N		0		0	R	S
S	CTX-M	DHA	CTX-M,D	3		0	R	R
S	SHV	DHA	SHV,DH/	3		0	R	S
S	CTX-M	DHA	CTX-M,D	3		0	R	S
S	CTX-M	DHA	CTX-M,D	3		0	R	S
R	N	N		0		0	R	S
S	N	DHA		2		0	R	R
S	CTX-M	DHA	CTX-M,D	3		0	R	S
S	N	N		0	KPC-2	0	R	R
S	N	N		0		0	R	S
S	N	MIR		2		0	R	S
S	CTX-M 8	DHA	CTX-M&	3		0	R	S
S	N	MIR		2		0	R	S
S	N	MIR		2		0	R	S
S	N	N		0		0	R	S
S	N	N		0		0	R	S
S	N	MIR		2		0	R	S
S	N	N		0		0	R	S
S	N	N		0		0	R	S

S	N	DHA		2	0	R	S
S	CTX-M	CMY	CTX-M,C	3	0	R	S
S	N	N		0	0	R	S
S	CTX-M	DHA	CTX-M,D	3	0	R	S
S	CTX-M	N		1	0	R	S
S	SHV	DHA	SHV,DH/	3	0	R	S
S	N	CMY		2	0	R	S
S	N	DHA		2	0	R	S
S	SHV	DHA	SHV,DH/	3	0	R	S
S	N	N		0	0	R	S
S	N	N		0	0	R	R
S	N	N		0	0	R	S
S	N	N		0	0	R	S
S	N	N		0	0	R	S
S	N	N		0	0	R	S
S	N	N		0	0	R	S
S	N	N		0	0	R	S
S	N	N		0	0	R	S
R	N	N		0	0	R	S
S	N	CMY		2	0	R	S
S	CTX-M	CMY	CTX-M,C	3	0	R	S
S	SHV	N		1	0	R	S
S	CTX-M	N		1	0	R	S
S	CTX-M	DHA	CTX-M,D	3	0	R	S
S	CTX-M 8	DHA	CTX-M&	3	0	R	S
R	N	N		0	0	R	R
S	N	DHA		2	0	R	R
S	N	DHA		2	0	R	S
S	N	DHA		2	0	R	S
S	CTX-M	DHA	CTX-M,D	3	0	R	S
S	N	DHA		2	0	R	S
S	CTX-M	DHA	CTX-M,D	3	0	R	R
S	N	CMY		2	0	R	S
R	N	N		0	0	R	R
S	N	N		0	0	R	S
S	N	N		0	0	R	S
R	SHV	N		1	0	R	S
S	CTX-M	CMY	CTX-M,C	3	0	R	S
S	N	DHA		2	0	R	S
S	N	CMY		2	0	R	R
S	CTX-M	N		1	0	R	S
S	SHV	N		1	0	R	S
S	CTX-M	N		1	0	R	S
S	CTX-M	N		1	0	R	S
S	CTX-M	DHA	CTX-M,D	3	0	R	S
S	SHV	DHA	SHV,DH/	3	0	R	S

S	CTX-M	DHA	CTX-M,D	3		0	R	S
S	N	CMY		2		0	R	R
S	N	DHA		2		0	R	R
S	N	CMY		2		0	R	R
S	N	CMY		2		0	R	R
S	N	N		0		0	R	S
S	N	N		0		0	R	S
S	N	N		0		0	R	S
S	N	N		0		0	R	S
S	N	MIR		2		0	R	S
S	N	N		0		0	R	S
S	N	MIR		2		0	R	R
S	N	N		0		0	R	S
S	N	N		0		0	R	S
S	N	N		0		0	R	R
R	N	N		0		0	R	S
S	CTX-M	N		1		0	R	S
S	CTX-M	CMY	CTX-M,C	3		0	R	S
S	SHV	DHA	SHV,DH	3		0	R	S
S	CTX-M	DHA	CTX-M,D	3		0	R	S
S	CTX-M &	DHA	CTX-M&	3		0	R	S
S	CTX-M	DHA	CTX-M,D	3		0	R	S
S	CTX-M	DHA	CTX-M,D	3		0	R	R
S	CTX-M	DHA	CTX-M,D	3		0	R	S
S	N	DHA		2		0	R	R
S	N	DHA		2		0	R	S
S	N	DHA		2		0	R	S
S	CTX-M	DHA	CTX-M,D	3		0	R	S
S	CTX-M &	N		1	VIM-1	0	R	R
S	N	DHA		2		0	R	S
S	N	DHA, CM		2		0	R	S
S	N	N		0		0	R	S
S	N	N		0		0	R	S
S	N	ACT		2		0	R	S
R	N	N		0		0	R	S
S	N	N		0		0	R	S
S	CTX-M	N		1		0	R	S
S	CTX-M	CMY	CTX-M,C	3		0	R	S
S	N	N		0		0	R	S
S	N	DHA		2		0	R	S
S	N	DHA		2		0	R	S
S	N	DHA		2		0	R	S
S	N	DHA		2		0	R	S
S	N	CMY		2		0	R	S
S	CTX-M	DHA	CTX-M,D	3		0	R	R
S	N	N		0		0	R	S

S	N	N		0		0	R	S
S	N	N		0		0	R	S
S	N	N		0		0	R	R
S	N	N		0		0	R	S
S	N	ACT		2		0	R	S
S	N	CMY		2		0	R	S
S	CTX-M	DHA	CTX-M,D	3		0	R	R
S	N	MIR		2		0	R	R
S	N	N		0		0	R	R
S	CTX-M	DHA	CTX-M,D	3		0	R	S
S	CTX-M	DHA	CTX-M,D	3		0	R	S
S	CTX-M	DHA	CTX-M,D	3		0	R	S
S	CTX-M	DHA	CTX-M,D	3		0	R	S
S	N	MIR		2		0	R	S
S	N	N		0		0	R	S
S	CTX-M	N		1		0	R	S
S	N	N		0		0	R	S
S	N	N		0		0	R	S
S	CTX-M	CMY	CTX-M,C	3		0	R	R
S	N	N		0	IMP-8	0	R	S
S	CTX-M	CMY	CTX-M,C	3	IMP-8	0	R	R
S	SHV	DHA	SHV,DH	3		0	R	R
S	N	N		0		0	R	S
S	N	N		0		0	R	S
S	N	DHA		2		0	S	R
S	N	DHA		2		0	R	S
S	SHV	N		1		0	R	S
S	N	N		0		0	R	S
S	SHV	DHA	SHV,DH	3		0	R	S
S	CTX-M	DHA	CTX-M,D	3		0	R	S
S	CTX-M	CMY	CTX-M,C	3		0	R	R
S	N	CMY		2		0	R	S
S	CTX-M	DHA	CTX-M,D	3		0	R	S
S	CTX-M	DHA	CTX-M,D	3		1	R	S
S	CTX-M	DHA	CTX-M,D	3		1	R	S
S	CTX-M	DHA	CTX-M,D	3		1	R	R
S	N	CMY		2		1	R	S
S	CTX-M &	CMY	CTX-M&	3		1	R	S
S	CTX-M	DHA	CTX-M,D	3		1	R	S
S	CTX-M	DHA	CTX-M,D	3		1	R	S
S	N	CMY		2		1	R	S
S	CTX-M	DHA	CTX-M,D	3		1	R	S
S	N	CMY		2		1	R	S
S	N	DHA		2		1	R	S
S	N	DHA		2		1	R	R
S	CTX-M	CMY	CTX-M,C	3		1	R	S

S	N	N		0		1	R	R
S	CTX-M	DHA	CTX-M,[illegible]	3		1	R	S
S	CTX-M	DHA	CTX-M,[illegible]	3		1	R	S
S	N	N		0		1	R	S
S	SHV	ACT	SHV,ACT	3	IMP-8	1	R	R
S	N	CMY		2		1	R	R
R	N	N		0	IMP-8	1	R	R
S	N	CMY		2		1	R	S
S	N	DHA		2		1	R	S
S	CTX-M	DHA	CTX-M,[illegible]	3		1	R	S
S	CTX-M	DHA	CTX-M,[illegible]	3		1	R	S
S	N	DHA		2		1	R	S
S	N	DHA		2		1	R	S
S	CTX-M	DHA	CTX-M,[illegible]	3		1	R	S
S	CTX-M	N		1		1	R	S
S	CTX-M	DHA	CTX-M,[illegible]	3		1	R	S
S	SHV	DHA	SHV,DH[illegible]	3		1	R	S
S	N	DHA		2		1	R	S
S	SHV	DHA	SHV,DH[illegible]	3		1	R	S
S	SHV	DHA	SHV,DH[illegible]	3		1	R	S
S	CTX-M [illegible]	N		1		1	R	S
R	N	DHA		2		1	R	S
S	CTX-M	DHA	CTX-M,[illegible]	3		1	R	S
R	SHV	DHA	SHV,DH[illegible]	3		1	R	S
S	N	DHA		2		1	R	S
S	CTX-M	DHA	CTX-M,[illegible]	3		1	R	S
S	N	DHA		2		1	R	S
S	SHV	DHA	SHV,DH[illegible]	3		1	R	S
S	N	N		0		1	R	S
S	CTX-M	N		1		1	R	S
S	CTX-M	CMY	CTX-M,[illegible]	3		1	R	S
S	SHV	DHA	SHV,DH[illegible]	3		1	R	S
S	SHV	DHA	SHV,DH[illegible]	3		1	R	S
S	N	DHA		2		1	R	R
S	CTX-M	DHA	CTX-M,[illegible]	3		1	R	S
S	CTX-M	DHA	CTX-M,[illegible]	3		1	R	S
S	N	DHA		2		1	R	R
S	N	DHA		2		1	R	S
S	N	DHA		2		1	R	S
S	CTX-M [illegible]	DHA	CTX-M&[illegible]	3		1	R	R
S	CTX-M	DHA	CTX-M,[illegible]	3		1	R	R
S	N	DHA		2		1	R	S
S	CTX-M	DHA	CTX-M,[illegible]	3		1	R	S
S	CTX-M	DHA	CTX-M,[illegible]	3		1	R	S
S	N	DHA		2		1	R	S
S	CTX-M	CMY	CTX-M,[illegible]	3		1	R	S

S	CTX-M	DHA	CTX-M,D	3		1	R	S
S	CTX-M	DHA	CTX-M,D	3		1	R	S
S	CTX-M	CMY	CTX-M,C	3		1	R	S
S	CTX-M	DHA	CTX-M,D	3		1	R	S
S	SHV	DHA	SHV,DHA	3		1	R	S
S	CTX-M	DHA	CTX-M,D	3		1	R	S
S	CTX-M	DHA	CTX-M,D	3		1	R	S
S	CTX-M	DHA	CTX-M,D	3		1	R	S
S	CTX-M	DHA	CTX-M,D	3		1	R	S
S	CTX-M &	DHA	CTX-M&	3		1	R	S
S	N	N		0	IMP-8	1	R	S
S	N	N		0	IMP-8	1	R	R
S	CTX-M	CMY	CTX-M,C	3		1	R	S
S	N	N		0		1	R	S
S	CTX-M	CMY	CTX-M,C	3		1	R	S
S	CTX-M	N		1		1	R	R
S	N	N		0	IMP-8	1	R	S
S	CTX-M	DHA	CTX-M,D	3		1	R	S
S	CTX-M	N		1		1	R	S
S	SHV	N		1	IMP-8	1	R	R
S	CTX-M	CMY	CTX-M,C	3		1	R	S
R	N	N		0	IMP-8	1	R	S
S	CTX-M	DHA	CTX-M,D	3		1	R	S
S	CTX-M	DHA	CTX-M,D	3		1	R	S
S	CTX-M	CMY	CTX-M,C	3		1	R	S
S	N	N		0		1	R	R
S	N	DHA		2		1	R	R
S	N	N		0		0	R	S
S	N	N		0		0	R	S
S	CTX-M	CMY	CTX-M,C	3		0	R	S
S	SHV	DHA	SHV,DHA	3		0	R	S
S	SHV	N		1		0	R	S
S	CTX-M	DHA	CTX-M,D	3		0	R	S
S	CTX-M	DHA	CTX-M,D	3		0	R	S
S	SHV	DHA	SHV,DHA	3		0	R	S
S	CTX-M	DHA	CTX-M,D	3		0	R	S
S	CTX-M	N		1		0	R	S
S	N	DHA		2		0	R	S
S	CTX-M	N		1	KPC-2	0	R	R
S	N	N		0	IMP-8	0	R	S
S	N	DHA		2		0	R	R
S	N	DHA		2		0	R	S
S	CTX-M	N		1		0	R	S
S	N	N		0		0	R	R
S	N	N		0		0	R	S
S	N	N		0		0	R	S

S	N	N		0		0	R	S
S	N	N		0		0	R	S
S	CTX-M	DHA	CTX-M,D	3		0	R	S
S	N	DHA		2		0	R	S
S	CTX-M 8	N		1	KPC-2	0	R	R
S	SHV	DHA	SHV,DHA	3		0	R	S
S	CTX-M	CMY	CTX-M,C	3		0	R	R
S	N	DHA		2		0	R	S
S	N	DHA		2		0	R	S
S	N	DHA		2		0	R	R
S	N	MIR		2		0	R	R
S	N	N		0		0	R	S
S	N	N		0		0	R	R
S	N	N		0		0	R	S
S	N	N		0		0	R	S
S	N	N		0		0	R	S
S	N	CMY		2		0	R	S
S	N	CMY		2		0	R	S
S	CTX-M	DHA	CTX-M,D	3		0	R	S
S	CTX-M	DHA	CTX-M,D	3		0	R	S
S	N	DHA		2		0	R	S
S	N	N		0		0	R	S
S	N	N		0		0	R	S
S	CTX-M	N		1		0	R	S
S	CTX-M	CMY	CTX-M,C	3		0	R	S
S	N	N		0		0	R	S
S	N	DHA		2		0	R	R
S	CTX-M	N		1		0	R	S
S	CTX-M	DHA	CTX-M,D	3		0	R	S
S	N	N		0		0	R	R
S	N	MIR		2		0	R	S
S	N	MIR		2		0	R	S
S	N	N		0		0	R	S
S	N	N		0		0	R	S
S	N	N		0		0	R	S
S	N	N		0		0	R	S
S	N	DHA		2		1	R	S
S	CTX-M	DHA	CTX-M,D	3		1	R	R
R	N	N		0		1	S	R
R	CTX-M	DHA	CTX-M,D	3		1	R	S
S	N	N		0		1	R	S
S	N	DHA		2		1	R	R
S	N	DHA		2		1	R	S
S	N	CMY		2		1	R	S
S	SHV	CMY	SHV,CMY	3	IMP-8	1	R	R
S	N	N		0		1	R	S

S	SHV	N		1		0	R	S
S	N	N		0		0	R	S
S	CTX-M	CMY	CTX-M,C	3		0	R	S
S	SHV	N		1		0	R	R
S	SHV	N		1		0	R	S
S	CTX-M	DHA	CTX-M,D	3		0	R	S
S	SHV	N		1		0	R	S
S	N	DHA		2		0	R	S
S	N	N		0		0	R	S
S	SHV	N		1		0	R	S
S	CTX-M	DHA	CTX-M,D	3		0	R	S
S	SHV	N		1		0	R	S
S	CTX-M 8	N		1	KPC-2	0	R	R
S	CTX-M	DHA	CTX-M,D	3		0	R	S
R	CTX-M	DHA	CTX-M,D	3	KPC-2	0	R	R
S	N	N		0		0	R	S
S	N	N		0		0	R	S
S	CTX-M 8	DHA	CTX-M&	3		0	R	S
S	CTX-M	N		1		0	R	R
S	SHV	N		1		0	R	S
S	CTX-M	DHA	CTX-M,D	3		0	R	S
S	N	N		0		0	R	R
S	N	DHA		2		0	R	R
S	CTX-M	CMY	CTX-M,C	3		0	R	S
S	N	DHA		2		0	R	S
S	CTX-M	DHA	CTX-M,D	3		0	R	S
S	SHV	CMY	SHV,CMY	3		0	R	R
S	CTX-M	DHA	CTX-M,D	3		0	R	S
S	CTX-M	DHA	CTX-M,D	3		0	R	S
S	CTX-M	DHA	CTX-M,D	3		0	R	S
S	CTX-M	DHA	CTX-M,D	3		0	R	R
S	CTX-M	DHA	CTX-M,D	3		0	R	R
S	N	ACT		2		0	R	S
S	N	ACT		2		0	R	S
S	CTX-M 8	N		1	KPC-2	0	R	R
S	SHV	N		1		0	R	S
S	N	DHA		2		0	R	R
S	CTX-M	DHA	CTX-M,D	3		0	R	S
S	CTX-M	DHA	CTX-M,D	3		0	R	S
S	N	N		0		0	R	R
S	SHV	ACT	SHV,ACT	3		0	R	S
S	N	N		0		0	R	S
S	CTX-M	DHA	CTX-M,D	3		0	R	S
R	N	N		0		0	R	R
S	CTX-M	N		1		0	R	S
S	CTX-M	CMY	CTX-M,C	3		0	R	S

S	SHV	N		1	IMP-8	0	S	R
S	N	CMY		2		0	R	S
R	N	DHA		2		0	R	R
S	CTX-M 8	DHA	CTX-M&	3		0	R	S
S	CTX-M	DHA	CTX-M,D	3		0	R	S
S	CTX-M	DHA	CTX-M,D	3		0	R	S
S	SHV	N		1		0	R	S
S	CTX-M	DHA	CTX-M,D	3		0	R	S
S	CTX-M	DHA	CTX-M,D	3		0	R	S
S	SHV	N		1		0	R	R
S	CTX-M	DHA	CTX-M,D	3		0	R	S
S	N	CMY		2		0	R	S
S	CTX-M	DHA	CTX-M,D	3		0	R	R
S	N	N		0		0	R	S
S	N	DHA		2		1	R	S
S	CTX-M 8	DHA	CTX-M&	3		1	R	S
S	CTX-M	N		1		1	R	S
S	CTX-M	DHA	CTX-M,D	3		1	R	S
S	SHV	DHA	SHV,DH/	3		1	R	S
R	N	N		0	IMP-8	1	R	S
S	CTX-M 8	DHA	CTX-M&	3		1	R	S
S	CTX-M	DHA	CTX-M,D	3		1	R	S
S	N	N		0		1	R	S
S	CTX-M	CMY	CTX-M,C	3	IMP-8	1	R	R
S	CTX-M	DHA	CTX-M,D	3		1	R	S
R	N	ACT		2	IMP-8	1	R	S
S	CTX-M	DHA	CTX-M,D	3		1	R	S
S	CTX-M	DHA	CTX-M,D	3		1	R	S
S	N	DHA		2		1	R	R
S	CTX-M	DHA	CTX-M,D	3		1	R	S
S	N	CMY		2		1	R	S
S	SHV	DHA	SHV,DH/	3		1	R	S
S	CTX-M 8	DHA	CTX-M&	3		1	R	S
S	SHV	DHA	SHV,DH/	3		1	R	S
S	SHV	N		1		1	R	R
S	CTX-M	DHA	CTX-M,D	3		1	R	S
R	N	DHA		2		1	R	S
S	N	N		0		1	R	S
S	N	ACT		2	IMP-8	1	R	R
S	N	N		0		1	R	S
R	N	N		0	IMP-8	1	R	S
S	CTX-M	DHA	CTX-M,D	3		1	R	S
S	SHV	DHA	SHV,DH/	3		1	R	S
S	CTX-M	DHA	CTX-M,D	3		1	R	S
S	CTX-M	DHA	CTX-M,D	3		1	R	S
S	N	CMY		2		1	R	R

R	N	ACT		2	IMP-8	1	R	S
S	CTX-M	DHA	CTX-M,D	3		1	R	S
R	N	ACT		2	IMP-8	1	R	S
S	CTX-M	N		1		1	R	S
S	CTX-M &	DHA	CTX-M&	3		1	R	S
S	N	CMY		2		1	R	R
S	CTX-M	DHA	CTX-M,D	3		1	R	S
S	N	N		0		1	R	R
S	N	N		0		1	R	S
S	N	DHA		2		1	R	S
S	CTX-M	DHA	CTX-M,D	3		1	R	S
S	SHV	DHA	SHV,DHA	3		1	R	S
S	SHV	DHA	SHV,DHA	3		1	R	S
S	SHV	DHA	SHV,DHA	3		1	R	R
S	CTX-M	DHA	CTX-M,D	3		1	R	S
S	CTX-M	DHA	CTX-M,D	3		1	R	S
S	N	N		0		1	R	S
S	N	ACT		2	IMP-8	1	R	R
S	CTX-M	DHA	CTX-M,D	3		1	R	S
S	N	DHA		2		1	R	S
R	SHV	ACT	SHV,ACT	3		1	R	S
S	CTX-M	DHA	CTX-M,D	3		1	R	S
S	N	DHA		2		1	R	R
S	CTX-M	DHA	CTX-M,D	3		1	R	S
S	CTX-M	N		1		1	R	S
S	CTX-M	CMY	CTX-M,C	3		1	R	R
S	N	MIR		2		0	R	S
S	SHV	ACT	SHV,ACT	3	IMP-8	0	R	R
S	CTX-M	N		1		0	R	S
S	CTX-M	N		1		0	R	S
S	CTX-M	DHA	CTX-M,D	3		0	R	S
S	CTX-M	DHA	CTX-M,D	3		0	R	S
R	CTX-M	N		1		0	R	S
R	N	N		0		0	R	S
S	SHV	DHA	SHV,DHA	3		0	R	R
S	CTX-M	DHA	CTX-M,D	3		0	R	S
S	CTX-M	DHA	CTX-M,D	3		0	R	S
S	CTX-M	DHA	CTX-M,D	3		0	R	S
R	N	DHA, CM		2		0	R	R
S	CTX-M	DHA	CTX-M,D	3		0	R	S
S	N	N		0		0	R	S
S	CTX-M	DHA	CTX-M,D	3		0	R	S
S	CTX-M	DHA	CTX-M,D	3		0	R	S
R	N	N		0		0	R	S
S	N	N		0		0	R	S
S	N	N		0		0	R	S

S	N	N		0	0	R	S
S	N	N		0	0	R	S
S	CTX-M	CMY	CTX-M,C	3	0	R	S
S	CTX-M	CMY	CTX-M,C	3	0	R	S
S	CTX-M	DHA	CTX-M,D	3	0	R	R
S	SHV	DHA	SHV,DH	3	0	R	R
R	N	DHA		2	0	R	S
S	N	DHA		2	0	R	S
S	CTX-M	DHA	CTX-M,D	3	0	R	R
S	N	DHA		2	0	R	S
R	N	DHA, CM		2	0	R	S
R	N	CMY		2	0	R	S
R	N	N		0	0	R	S
S	N	N		0	0	R	S
S	N	MIR		2	0	R	S
S	N	N		0	0	R	S
S	CTX-M	CMY	CTX-M,C	3	0	R	S
S	N	CMY		2	0	R	S
S	CTX-M &	DHA	CTX-M&	3	0	R	S
S	CTX-M	N		1	0	R	S
S	N	CMY		2	0	R	S
S	N	DHA, CM		2	0	R	S
S	N	DHA		2	0	R	S
S	CTX-M	DHA	CTX-M,D	3	0	R	S
S	N	DHA		2	0	R	S
S	N	N		0	0	R	S
S	N	N		0	0	R	S
S	CTX-M	DHA	CTX-M,D	3	0	R	S
R	N	N		0	0	R	S
S	N	CMY		2	0	R	S
S	CTX-M	N		1	0	R	S
S	CTX-M	DHA	CTX-M,D	3	0	R	S
S	CTX-M	DHA	CTX-M,D	3	0	R	S
S	CTX-M	N		1	0	R	S
S	CTX-M &	DHA	CTX-M&	3	0	R	R
S	N	DHA		2	0	R	S
S	CTX-M	DHA	CTX-M,D	3	0	R	S
S	N	DHA, CM		2	0	R	S
S	CTX-M	DHA	CTX-M,D	3	0	R	S
S	N	DHA, CM		2	0	R	S
S	CTX-M	DHA	CTX-M,D	3	0	R	S
S	N	DHA		2	0	R	S
S	N	N		0	0	R	S
S	N	N		0	0	R	S
S	N	N		0	0	R	R
S	N	N		0	0	R	S

S	N	N		0		0	R	R
S	N	N		0		0	R	S
S	N	N		0		0	R	R
S	N	N		0		0	R	S
S	N	MIR		2		0	R	S
S	CTX-M	N		1		0	R	R
S	N	CMY		2		0	R	R
S	N	CMY		2		0	R	S
S	CTX-M &	DHA	CTX-M&	3		0	R	R
S	CTX-M	DHA	CTX-M,D	3		0	R	S
S	SHV	N		1		0	R	S
S	CTX-M &	N		1	KPC-2	0	R	R
S	N	DHA		2		0	R	S
R	CTX-M	DHA	CTX-M,D	3		0	R	S
S	CTX-M	DHA	CTX-M,D	3		0	R	S
S	CTX-M	DHA	CTX-M,D	3		0	R	S
S	N	N		0		0	R	S
S	CTX-M	DHA	CTX-M,D	3		0	R	R
S	N	DHA		2		0	R	S
S	CTX-M	CMY	CTX-M,C	3		0	R	S
S	N	N		0		0	R	S
R	N	N		0		0	R	R
S	N	N		0		0	R	S
S	N	MIR		2		0	R	S
S	CTX-M	N		1		0	R	S
S	N	CMY		2		0	R	S
S	N	CMY		2		0	R	R
S	CTX-M	DHA	CTX-M,D	3		0	R	S
S	CTX-M	DHA	CTX-M,D	3		0	R	S
S	CTX-M	DHA	CTX-M,D	3		0	R	S
S	N	DHA		2		0	R	S
S	N	CMY		2		0	R	R
S	CTX-M	DHA	CTX-M,D	3		0	R	R
S	N	DHA		2		0	R	S
S	N	N		0		0	R	R
S	N	N		0		0	S	R
S	CTX-M &	CMY	CTX-M&	3		0	R	S
S	N	N		0		0	R	R
S	N	N		0		0	R	S
S	N	N		0		0	R	S
S	N	CMY		2		0	R	S
S	N	CMY		2		0	R	S
S	N	CMY		2		0	R	S
R	CTX-M	DHA	CTX-M,D	3		0	R	S
S	CTX-M	DHA	CTX-M,D	3		0	R	S
S	CTX-M	DHA	CTX-M,D	3		0	R	S

S	CTX-M	DHA	CTX-M,D	3	0	R	S
S	CTX-M	DHA	CTX-M,D	3	0	R	S
S	CTX-M	DHA	CTX-M,D	3	0	R	R
S	CTX-M	DHA	CTX-M,D	3	0	R	S
R	N	DHA		2	0	R	S
R	N	N		0	0	R	S
S	N	N		0	0	R	S
S	N	MIR		2	0	R	S
S	N	N		0	0	R	S
S	N	N		0	0	R	S
S	CTX-M	CMY	CTX-M,C	3	0	R	S
S	N	CMY		2	0	R	R
S	N	CMY		2	0	R	S
S	CTX-M	DHA	CTX-M,D	3	0	R	S
S	CTX-M &	DHA	CTX-M&	3	0	R	S
S	CTX-M	N		1	0	R	S
S	CTX-M &	DHA	CTX-M&	3	0	R	S
S	CTX-M	DHA	CTX-M,D	3	0	R	S
S	CTX-M	DHA	CTX-M,D	3	0	R	S
S	CTX-M	DHA	CTX-M,D	3	0	R	S
S	N	DHA		2	0	R	S
S	N	ACT		2	0	R	R
R	SHV	N		1	0	R	S
S	N	ACT		2	0	R	R
R	N	N		0	0	R	S
S	CTX-M	CMY	CTX-M,C	3	0	R	S
S	CTX-M	DHA	CTX-M,D	3	0	R	S
S	CTX-M	DHA	CTX-M,D	3	0	R	S
S	CTX-M	DHA	CTX-M,D	3	0	R	S
S	N	DHA		2	0	R	S
S	N	DHA		2	0	R	S
S	N	DHA		2	0	R	S

MEM_g	Carbape	MLST	Replicon_typing
S	N		
S	N		
S	IMP-8		A/C
S	N		
S	N		
S	N		
S	N		
S	N		
S	N		
S	N		
S	N		
S	N		
S	N		
S	N		
S	N		
S	N		
S	N		
S	N		
S	IMP-8		negative
S	N		
S	N		
S	N		
S	N		
S	N		
S	N		
S	N		
S	N		
S	IMP-8		negative
S	N		
S	N		
S	N		
S	N		
S	N		
S	N		
S	N		
S	N		
S	N		
S	N		
S	N		
S	N		
S	N		
S	N		
S	N		
S	N		
S	N		
S	N		

S	N		
S	N		
S	N		
S	N		
S	N		
R	N		
S	N		
S	N		
S	N		
S	N		
S	N		
S	N		
S	N		
S	N		
R	KPC-2	ST11	A/C
S	N		
R	N		
S	N		
S	N		
S	N		
S	N		
S	N		
R	N		
S	N		
R	N		
S	N		
S	N		
S	N		
S	N		
S	N		
S	N		
S	N		
S	N		
R	N		
R	N		
S	N		
S	N		
S	N		
S	N		
S	N		
S	N		
R	N		
R	N		
S	N		
S	N		
R	N		

S	N	
S	N	
S	N	
S	N	
S	N	
S	N	
S	N	
R	N	
S	N	
S	N	
R	NDM-1	negative
S	N	
S	N	
S	N	
R	N	
S	N	
S	N	
S	N	
S	N	
R	N	
S	N	
S	N	
S	N	
S	N	
S	IMP-8	A/C
R	N	
S	N	
R	N	
S	N	
S	N	
S	N	
S	N	
S	N	
R	N	
S	N	
S	N	
S	N	
S	N	
R	N	
S	N	
S	N	
S	N	
S	N	
S	N	
R	N	
S	N	

S	N
S	N
S	N
S	N
S	N
S	N
S	N
S	N
S	N
S	N
S	N
S	N
S	N
S	N
S	N
S	N
S	N
S	N
R	N
S	N
S	N
R	N
S	N
S	N
S	N
R	N
S	N
S	N
S	N
S	N
S	N
S	N
S	N
R	N
S	N
S	N
S	N
S	N
S	N
S	N
S	N
S	N
R	N
S	N
S	N
S	N

S	N	
S	N	
R	N	
S	N	
S	N	
R	IMP-8	negative
S	N	
S	N	
S	N	
S	N	
S	N	
S	N	
S	N	
S	N	
S	N	
S	N	
S	N	
S	N	
S	N	
R	N	
S	N	
S	N	
S	N	
S	N	
S	N	
S	N	
S	N	
S	N	
S	IMP-8	negative
S	N	
S	N	
S	N	
S	N	
S	N	
S	N	
R	N	
S	N	
S	N	
S	N	
S	N	
S	N	
S	N	
S	N	
S	N	
S	IMP-8	negative
S	N	

S	N		
S	N		
S	N		
S	N		
S	N		
S	N		
R	N		
S	N		
S	N		
S	N		
S	N		
S	N		
S	N		
S	N		
S	N		
S	N		
R	N		
S	N		
S	N		
S	N		
S	N		
S	N		
S	N		
S	N		
S	N		
S	N		
S	N		
S	N		
S	N		
S	N		
S	N		
S	N		
S	N		
S	N		
S	N		
R	N		
S	N		
S	N		
R	N		
S	N		
S	N		
S	N		
S	N		
R	KPC-2	ST35	N, FIA, FIB, F
S	N		
S	N		

S	N	
S	N	
S	N	
S	N	
S	N	
S	N	
S	N	
S	N	
S	N	
S	N	
S	N	
S	N	
S	N	
S	N	
S	N	
S	N	
S	N	
S	N	
S	N	
S	N	
S	N	
S	N	
S	N	
S	N	
S	N	
S	N	
S	N	
R	N	
R	N	
R	N	
R	N	
R	N	
S	N	
S	N	
S	N	
R	IMP-8	negative
S	N	
R	N	
S	N	
S	N	
S	N	
S	N	
S	N	
S	N	
S	N	
S	N	

S	N		
S	N		
S	N		
S	N		
S	N		
S	N		
S	N		
S	N		
S	N		
R	IMP-8		negative
S	N		
S	N		
S	IMP-8		F
S	N		
S	N		
S	N		
S	N		
S	N		
S	N		
S	N		
R	N		
S	N		
S	N		
S	N		
R	N		
S	N		
R	VIM-1		negative
S	N		
R	KPC-2	ST11	FIA, F
S	N		
S	N		
S	N		
S	N		
S	N		
S	N		
S	N		
S	N		
S	N		
S	N		
S	N		
S	N		
S	N		
S	N		
R	KPC-2	ST11	F
R	KPC-2	ST11	F
R	KPC-2	ST11	F

S	N		
S	N		
S	N		
S	N		
R	KPC-2	ST11	A/C, F
R	KPC-2	ST11	FIB, F
S	N		
S	N		
S	N		
S	N		
S	N		
S	N		
S	N		
S	N		
S	N		
S	N		
S	N		
S	N		
S	N		
S	N		
R	N		
S	N		
S	N		
S	N		
S	N		
S	N		
S	N		
S	IMP-8		negative
S	N		
S	N		
S	IMP-8		negative
S	N		
S	N		
S	N		
S	N		
S	N		
S	N		
S	N		
S	N		
S	N		
S	N		
R	N		
S	N		
S	N		
R	N		
S	N		

S	N	
S	N	
S	N	
S	N	
S	N	
S	N	
R	N	
S	N	
S	N	
R	IMP-8	A/C, FIIAs, F
S	N	
S	N	
S	N	
S	N	
S	N	
S	N	
S	N	
S	N	
S	N	
S	N	
S	N	
S	N	
S	N	
S	N	
R	N	
S	N	
S	N	
S	N	
S	N	
S	N	
S	N	
S	N	
R	N	
S	N	
S	N	
S	N	
S	N	
S	N	
S	N	
S	N	
S	N	
S	N	
S	N	
S	N	
S	N	
S	N	

S	N		
S	N		
S	N		
S	N		
S	N		
S	N		
S	N		
S	N		
S	N		
S	N		
S	N		
S	N		
R	KPC-2	ST11	N, F
S	N		
S	N		
S	N		
S	N		
S	N		
S	N		
R	N		
S	N		
S	N		
S	N		
S	N		
S	IMP-8	ST15	A/C
S	N		
S	N		
S	N		
S	N		
S	N		
S	N		
R	KPC-2	ST11	F
S	N		
S	N		
S	N		
S	N		
S	N		
R	N		
S	N		
S	N		
S	N		
S	N		
S	N		
S	IMP-8		negative
R	N		
S	N		

S	N		
S	N		
S	N		
S	N		
S	N		
S	N		
S	N		
S	N		
S	N		
S	N		
R	N		
S	N		
S	N		
S	N		
S	N		
S	N		
R	N		
S	N		
S	N		
S	N		
S	N		
S	N		
S	N		
S	N		
S	N		
S	N		
S	N		
S	N		
S	N		
S	N		
S	N		
S	N		
S	N		
S	N		
S	N		
R	N		
S	N		
S	N		
S	N		
S	N		
S	N		
S	N		
S	N		
S	N	ST43	FIA, FIB, F
S	N		
S	N		

R	N		
S	N		
S	N		
S	N		
S	N		
S	N		
R	N		
R	N		
S	IMP-8		negative
S	N		
S	N		
S	N		
R	N		
S	N		
R	N		
S	N		
S	N		
S	N		
S	N		
S	N		
S	N		
S	N		
S	N		
R	N		
R	N		
S	N		
S	N		
S	N		
R	N		
S	N		
S	N		
S	N		
S	N		
S	N		
S	N		
R	KPC-2	ST11	A/C, F
S	N		
S	N		
S	N		
S	N		
S	N		
S	N		
S	N		
S	N		
S	N		
S	N		

S	N		
S	N		
S	N		
S	N		
S	N		
S	N		
S	N		
S	N		
S	N		
S	N		
R	N		
S	N		
S	N		
S	N		
S	N		
S	N		
S	N		
S	N		
S	N		
S	N		
S	N	ST43	I1-Iγ, FIA, FIB, F
S	N		
S	N		
S	N		
S	N		
R	N		
S	N		
S	N		
S	N		
S	N		
S	N		
R	N		
S	N		
R	N		
S	N		
S	N		
S	N		
S	N		
S	N		
R	N		
S	N		
S	N		
S	N		
S	N		
S	N		
S	N		

S	N		
R	N		
S	N		
S	N		
R	N		
S	N		
S	N		
S	N		
S	N		
S	N		
S	N		
S	N		
S	N		
S	N		
S	N		
S	N		
S	N		
S	N		
S	N		
S	N		
S	N		
S	N		
R	N		
S	N		
S	N		
S	N		
S	N		
S	N		
R	VIM-1	ST?	negative
S	N		
S	N		
S	N		
S	N		
S	N		
S	N		
S	N		
S	N	ST43	I1-Iγ, FIA, FIB, F
S	N		
S	N		
S	N		
S	N		
S	N		
S	N		
S	N		
R	N		
S	N		

S	N		
S	N		
S	N		
S	N		
S	N		
S	N		
R	N		
S	N		
S	N		
S	N		
S	N		
S	N		
S	N		
S	N		
S	N		
S	N		
S	N		
S	N		
S	N		
S	IMP-8		negative
S	IMP-8	ST?	FIA, A/C, F
R	N		
S	N		
S	N		
S	N		
S	N		
S	N		
S	N		
S	N		
S	N		
R	N		
S	N		
S	N		
S	N		
S	N		
S	N		
S	N		
S	N		
S	N		
S	N		
S	N		
S	N		
S	N		
S	N		
R	N		
S	N		

S	N	
S	N	
S	N	
S	N	
S	IMP-8	negative
R	N	
R	IMP-8	negative
S	N	
S	N	
S	N	
S	N	
S	N	
S	N	
S	N	
S	N	
S	N	
S	N	
S	N	
S	N	
S	N	
S	N	
S	N	
S	N	
S	N	
S	N	
S	N	
S	N	
S	N	
S	N	
S	N	
S	N	
S	N	
S	N	
S	N	
S	N	
S	N	
S	N	
S	N	
S	N	
R	N	
S	N	
S	N	
S	N	
S	N	
S	N	
S	N	

S	N		
S	N		
S	N		
S	N		
S	N		
S	N		
S	N		
S	N		
S	N		
S	N		
S	IMP-8		negative
S	IMP-8		negative
S	N		
S	N		
S	N		
R	N		
S	IMP-8		negative
S	N		
S	N		
S	IMP-8		negative
S	N		
S	IMP-8		negative
S	N		
S	N		
S	N		
R	N		
S	N		
S	N		
S	N		
S	N		
S	N		
S	N		
S	N		
S	N		
S	N		
S	N		
S	N		
S	N		
R	KPC-2	ST11	N, F
S	IMP-8		negative
S	N		
S	N		
S	N		
S	N		
S	N		
S	N		

S	N		
S	N		
S	N		
S	N		
R	KPC-2	ST11	F
S	N		
S	N		
S	N		
S	N		
R	N		
S	N		
S	N		
S	N		
S	N		
S	N		
S	N		
S	N		
S	N		
S	N		
S	N		
S	N		
S	N		
S	N		
S	N		
S	N		
S	N		
R	N		
R	N		
S	N		
S	N		
S	N		
S	N		
S	N		
S	N		
S	N		
S	N		
S	N		
R	N		
S	N		
S	N		
S	N		
S	N		
S	N		
S	N		
S	IMP-8		negative
S	N		

S	N		
S	N		
S	N		
S	N		
S	N		
S	N		
S	N		
S	N		
S	N		
S	N		
S	N		
S	N		
R	KPC-2	ST11	F
S	N		
R	KPC-2	ST11	A/C, F
S	N		
S	N		
S	N		
R	N		
S	N		
S	N		
R	N		
R	N		
S	N		
S	N		
S	N		
R	N		
S	N		
S	N		
S	N		
S	N		
R	N		
S	N		
S	N		
R	KPC-2	ST11	F
S	N		
R	N		
S	N		
S	N		
S	N		
S	N		
S	N		
S	N		
S	N		
S	N		
S	N		

S	IMP-8	negative
S	N	
R	N	
S	N	
S	N	
S	N	
S	N	
S	N	
S	N	
S	N	
S	N	
S	N	
S	N	
S	N	
S	N	
S	N	
S	N	
S	N	
S	N	
S	IMP-8	negative
S	N	
S	N	
S	N	
S	IMP-8	negative
S	N	
S	IMP-8	negative
S	N	
S	N	
S	N	
S	N	
S	N	
S	N	
S	N	
S	N	
R	N	
S	N	
S	N	
S	N	
S	IMP-8	negative
S	N	
S	IMP-8	negative
S	N	
S	N	
S	N	
S	N	
R	N	

S	IMP-8	negative
S	N	
S	IMP-8	negative
S	N	
S	N	
S	N	
S	N	
R	N	
S	N	
S	N	
S	N	
S	N	
S	N	
R	N	
S	N	
S	N	
S	N	
S	IMP-8	negative
S	N	
S	N	
S	N	
S	N	
R	N	
S	N	
S	N	
S	N	
S	N	
S	IMP-8	negative
S	N	
S	N	
S	N	
S	N	
S	N	
S	N	
S	N	
S	N	
S	N	
S	N	
S	N	
S	N	
S	N	
S	N	
S	N	
S	N	
S	N	
S	N	

S	N
S	N
S	N
S	N
S	N
R	N
S	N
S	N
R	N
S	N
S	N
S	N
S	N
S	N
S	N
S	N
S	N
S	N
S	N
S	N
S	N
S	N
S	N
S	N
S	N
S	N
S	N
S	N
S	N
S	N
S	N
S	N
S	N
S	N
S	N
S	N
S	N
S	N
S	N
S	N
S	N
S	N
S	N
S	N
S	N
S	N

S	N		
S	N		
S	N		
S	N		
S	N		
R	N		
S	N		
S	N		
S	N		
S	N		
S	N		
R	KPC-2	ST11	N, F
S	N		
S	N		
S	N		
S	N		
S	N		
R	N		
S	N		
S	N		
S	N		
R	N		
S	N		
S	N		
S	N		
S	N		
S	N		
S	N		
S	N		
S	N		
S	N		
R	N		
S	N		
S	N		
S	N		
S	N		
S	N		
S	N		
S	N		
S	N		
S	N		
S	N		
S	N		
S	N		
S	N		
S	N		

S	N
S	N
R	N
S	N
S	N
S	N
S	N
S	N
S	N
S	N
S	N
R	N
S	N
S	N
S	N
S	N
S	N
S	N
S	N
S	N
S	N
S	N
S	N
S	N
S	N
S	N
S	N
S	N
S	N
S	N
S	N
S	N
